# The Architect of Trust: Dr Tom Ferguson’s Legacy

**DOI:** 10.2196/97659

**Published:** 2026-08-07

**Authors:** Amy Price, Dave deBronkart

**Affiliations:** 1 Department of Community and Family Medicine (CFMED), Dartmouth–Hitchcock Medical Center, 100 Hitchcock Way, Manchester, NH, 03104, United States, 1 561 843 7372; 2 Center for Global Health, Colorado School of Public Health, Aurora, CO, United States; 3 JMIR Publications, Toronto, ON, Canada; 4 e-Patient Dave, LLC, Nashua, NH, United States

**Keywords:** participatory medicine, e-patients, Tom Ferguson, preference erasure, health care infrastructure, patient empowerment, coproduction, patient involvement, patients included, everyone included

## Abstract

This article serves as a formal tribute to the legacy of Dr Tom Ferguson and his foundational 2007 white paper on the “e-patient.” Despite its widespread influence, Dr Ferguson’s original vision has often been internalized by the broader medical community without clear historical attribution, leading to a drift in the term’s original meaning. This article restores the intellectual genealogy of the “e-patient” as defined by Dr Ferguson as “equipped, enabled, empowered, and engaged.” We argue for the continued relevance of his “quiet giant” leadership. By formalizing Dr Ferguson’s foundational white paper *e-Patients: How They Can Help Us Heal Healthcare* within this article, we ensure its preservation for future research and provide a stable foundation for the evolving field of participatory medicine.

## Introduction

This article is a tribute to Dr Tom Ferguson (1943-2006), one of the “quiet giants” of health care, who worked beyond the traditional spotlight to build something profoundly human. Dr Ferguson built an architecture of trust with patients and clinicians in the information age, and this vision became the architecture on which the Society of Participatory Medicine was founded.

*Clean, clear knowledge is the equivalent in this century of clean, clear water in the 19th century – it’s a public health service, people need access to it. Knowledge is the enemy of disease*.

—Sir Muir Gray [1]

It is a privilege for the *Journal of Participatory Medicine* to formally share Dr Ferguson’s foundational white paper *e-Patients: How They Can Help Us Heal Healthcare* (accessible via Multimedia Appendix 1) as both a historical record and a blueprint for our future. This future demands an infrastructure where difficult stories and lived histories are integrated from the start. We must actively resist what health economist Professor Jack Dowie coined “preference erasure” [2,3], which is the systemic process whereby individual patient preferences are stripped away when entered into the scientific record, leaving patients with no more “real estate” in the final evidence base than a forgotten grave.

Twenty-five years ago, Dr Ferguson articulated a radical and enduring vision: that patients could be the master architects of their self-care. Tom did not see a hierarchy of credentials; he saw a community of problem-solvers. He coined a term that reshaped our language: the “e-patient” [4]. He said “e-patient” was to “patient” as “email” was to “mail.” He later added more descriptive “e-words”: “equipped, engaged, empowered, and emancipated.”

He spoke from experience. Diagnosed around 1991 with multiple myeloma, he faced a median survival of 2 to 4 years but lived 15, dying in 2006. The visionary friends he’d accumulated, who practiced what he preached, formed the Society for Participatory Medicine.

**Figure 1.**
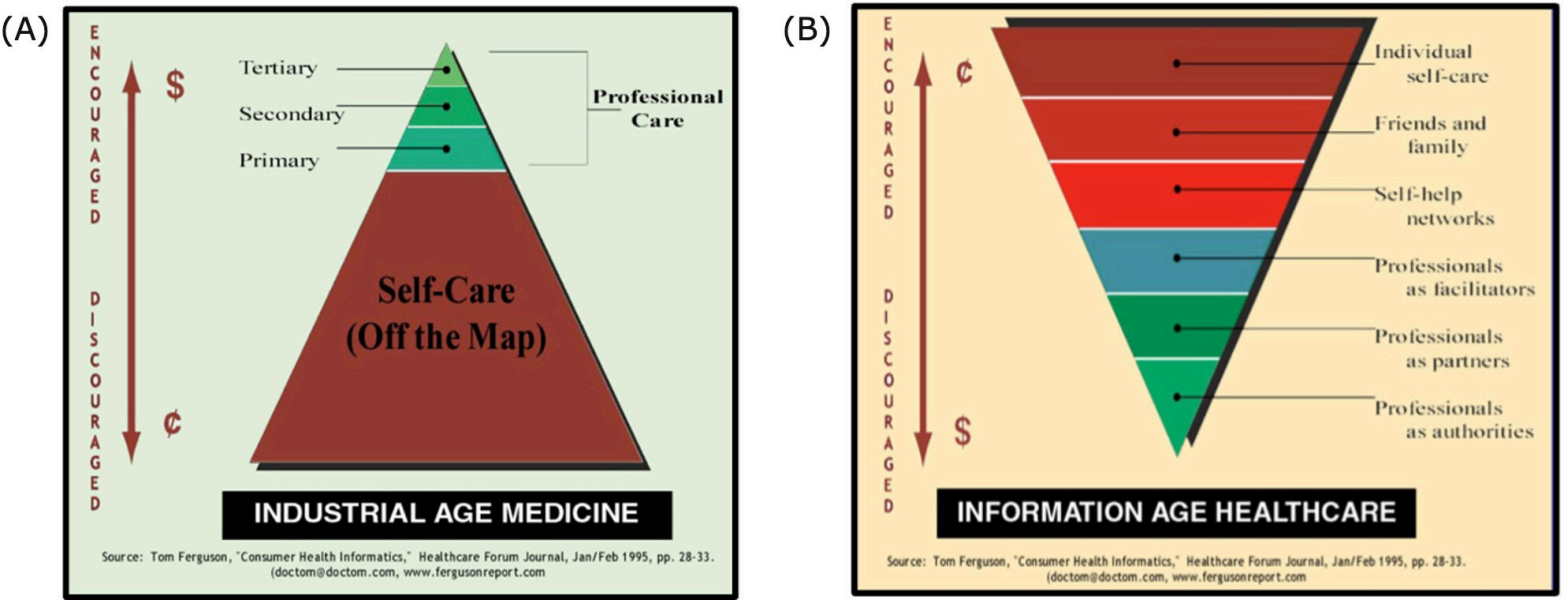
The “flip-the-triangle” paradigm, showing the shift in patient-physician engagement from the (A) Industrial Age to the (B) Information Age. These images were first published in January 1995 [5,6].

Dr Ferguson described “medical self-care” while still in medical school in the 1970s, and when the World Wide Web emerged, he envisioned the profound transformation it would be for patients. In January 1995, within a year of the release of the first popular web browser (Mozilla), he published a pair of “triangle slides” showing the inversion of power as medicine shifted from the Industrial Age to the Information Age (Figure 1).

The Industrial Age model placed physicians at the apex, with authority, knowledge, and power flowing downward to passive patients, whose self-care was not even recognized as existing. The revolution Dr Ferguson foresaw was simple: as information access became democratized, it would flip the triangle, giving power to self-care and self-help networks. In chronic disease, rare disease, and the management of one’s own life, the patient has first-hand expertise. The clinician becomes the consultant. It is noteworthy to view the accuracy of Dr Ferguson’s perceptions of ways the new paradigm could influence costs and social approvals.

“If there’s a ‘George Washington’ of the empowered patient movement, it’s Doctor Tom Ferguson,” wrote Elizabeth Cohen, the senior medical correspondent for CNN in 2008. She noted, “In 1975, at a time when many doctors were still viewed as all-knowing and infallible, Ferguson started writing about patients advocating for their own health care” [7]. A timeline of Dr Ferguson’s advocacy and teaching is shown in Table 1.

**Table 1.** Key milestones in Dr Tom Ferguson’s advocacy and teaching.

Date	Milestone
1975	Begins writing about patient advocacy and self-care while still in medical school
1975	Initiates publication of the *Medical Self-Care* newsletter (through 1989)
1978	Interviewed in *Mother Earth News* and by Dan Rather on *60 Minutes* [8] (Figure 2)
1979	Graduates from Yale Medical School
1980	Publishes book *Medical Self-Care: Access to Health Tools* [9]
1994	Becomes medical editor of the *Whole Earth Catalog, Millennium Edition* (Health Section) [10]
1995	Publishes his “triangle slides” (Figure 1) depicting “Information Age Healthcare” as an inversion of the power structure of “Industrial Age Medicine”
1996	At the dawn of the web, publishes *Health Online: How To Find Health Information, Support Groups, And Self-Help Communities In Cyberspace* [11]
1998	Publishes first *JAMA* editorial: “Digital Doctoring—Opportunities and Challenges in Electronic Patient-Physician Communication” [12]
1999	Begins *The Ferguson Report: The Newsletter of Consumer Health Informatics and Online Health* (through 2003) [6]
2000	Publishes first article in *The BMJ*: “Online patient-helpers and physicians working together,” foretelling participatory medicine [13]
2004	Publishes “The First Generation of e-Patients” in *The BMJ* [4]
2006	Dr Tom Ferguson passes away; the *New York Times* publishes his obituary [14]
2007	*e-Patients: How They Can Help Us Heal Healthcare* completed and published by his colleagues
2009	The Society for Participatory Medicine formally founded
2026	The *Journal of Participatory Medicine* enters the white paper into the record as a multimedia appendix in *The Architect of Trust: Dr Tom Ferguson’s Legacy*

**Figure 2.**
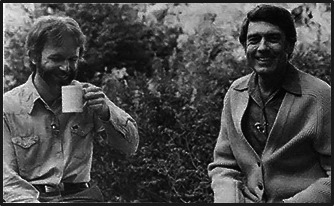
A young Dr Tom Ferguson (left) being interviewed by Dan Rather (right) for *60 Minutes*: Photo used with permission of *Mother Earth News* (Ogden Publishers), 1979.

## Why the Comparison Fits

The CNN article and the *Journal of Participatory Medicine* draw several parallels between Dr Ferguson and the Founding Fathers of the United States:

- Pioneering spirit: Just as George Washington led a new era of self-governance, Tom Ferguson began his work in 1975 by founding *Medical Self-Care* magazine, challenging the “Industrial Age” model where doctors held absolute authority.
- A “manifesto” for freedom: Dr Ferguson was working on his final manifesto, *e-Patients: How They Can Help Us Heal Healthcare*, at the time of his death in 2006. Elliot Stone, one of his colleagues, noted that reading his early drafts felt like “looking over Thomas Paine’s shoulder.”
- The power of the people: his vision centered on the idea that “we the people” (the patients) could create a new reality with a balanced power dynamic [12]. He famously argued that when patients are well-informed, they can be trusted to manage their own health [15], much like Thomas Jefferson’s belief in self-governance and the economist and Nobel laureate Elinor Ostrom’s work *Governing the Commons: The Evolution of Institutions for Collective Action* [16].

We hear of “patient engagement” described as an adjunct to care. Yet the true history of participatory medicine was written by those who did not wait for permission [17]. These are the advocates who conducted interviews from beds in the intensive care unit [18], endured rejection by systems they sought to improve [19], and banded together to provide palliative care when the world looked away [20].

Dr Ferguson’s vision was partnership, not a power hierarchy. It was a recognition that patients are the primary providers of health care whose contributions have historically been limited by their access to knowledge. He worked to erase those limits, as the Society of Participatory Medicine does today. Across conditions, geographies, and decades, e-patients have demonstrated three enduring truths:

- The catalytic force: patients have repeatedly served as the catalyst translating research into services, identifying patterns and outcomes that professionals miss. They define empathy as constructive action rather than emotional obligation [21].
- Bypassing the “lethal lag time”: while institutional research can be slowed by the lethal lag time (ie, the average 17-year lag between new medical practices being reported and adopted), patient-led networks have shared life-saving insights in real time. Dr Ferguson documented this long before the “17-year problem” was first described in 2001 [22].
- The uncredited labor: from the discovery of unexpected drug effects to the mapping of rare genetic disorders [23], e-patients have driven major advances, often without recognition. A mature participatory system must learn not only from this work, but to properly acknowledge its sources [24] so that future research can learn from patient contributions to knowledge and care.

## Growing the Standard of Humanity

The white paper *e-Patients: How They Can Help Us Heal Healthcare* (Multimedia Appendix 1) is more than an archive; it serves as a standard of humanity. We define this standard not merely as a historical record, but as a commitment to humane medical care, a virtue derived from the human condition that actively supports love and compassion. We honor those who managed the estates of destitute colleagues and restored dignity to those the world had cut out. They proved that durable innovation emerges only where relationships are valued and the human element is treated as essential infrastructure.

Today we know that the e-patient is not a niche role or a transitional phase. It is a force of nature shaping destiny. But this was not at all apparent when Dr Ferguson began his work. Over decades, this movement has grown from a fragile seed to a vulnerable shoot, and finally to a strong, fruit-bearing tree. Its viability is no longer threatened by those who once sought to uproot it. It has become a shelter, broad enough to hold even its former skeptics, sustained by the very collaboration it made possible.

E-patients continue to build the infrastructure, share their stories, and bear the burden of crisis together as they pioneer innovation. We are witnessing transformative knowledge for self-care fueled by large language models democratizing and organizing information that was formerly unavailable to patients when it lived within inaccessible institutional paywalls [25,26].

*What makes this moment structurally different is not the AI. It is whether we build the literacy infrastructure alongside the technology or after the harm accumulates. Let’s not repeat the pattern of building patient portals without training patients to use them*.

—Gilles Frydman [27]

At the *Journal of Participatory Medicine*, we are not waiting for institutions to change. Like the e-patients with type 1 diabetes who declared the #WeAreNotWaiting hashtag and created their own treatment [28], we are building the future alongside them, beginning with the recognition of those who have already led the way.

These contributions remind us that lasting progress arises not from abstraction, but from everyday people who dare to rise, work together, and insist that trust itself be treated as essential infrastructure.

*We are all fixing what is broken. It is the task of a lifetime. We'll leave much unfinished for the next generation.*

—Abraham Verghese [29]

This article is intended as a historical document. *e-Patients: How They Can Help Us Heal Healthcare* deserves a permanent home in the scientific archives rather than remaining as an obscure PDF on a society’s back pages. By providing clear history and attribution, we ensure that resources like the James Lind Library can accurately catalog the origins of this movement, giving Dr Ferguson the honor his work deserves.

We recommend readers download and read the 2007 white paper *e-Patients: How They Can Help Us Heal Healthcare* via Multimedia Appendix 1.

### Suggested Citation

Ferguson T, e-Patients Scholars Working Group. e-Patients: How They Can Help Us Heal Healthcare. Dreiss M, Fox S, Frydman G, et al, editors. (originally published in 2007). In: Price A, deBronkart D. The architect of trust: Dr Tom Ferguson’s legacy. J Particip Med 2026;18:e97659.

### Participatory Medicine Declaration

This work and the white paper *e-Patients: How They Can Help Us Heal Healthcare* were co-produced by patients and clinicians.

## Supplementary material

10.2196/97659 Multimedia Appendix 1 e-Patients: How They Can Help Us Heal Healthcare.
