# Supplementary material for: The Architect of Trust: Dr Tom Ferguson’s Legacy
Source: J Particip Med. 2026 Aug 7;18:e97659. doi: 10.2196/97659 (PMC13450881; doi:10.2196/97659)
Supplement: Supplementary file 1 [file jopm-v18-e97659-s001.pdf]

"I felt I was looking over Thomas Paine's shoulder"
– Elliott M. Stone

e-patients

how they can help us heal health care

Tom Ferguson, MD
and the e-Patient Scholars
Working Group

e-patients: how they can help us heal healthcare

Tom Ferguson, MD
with the e-Patients Scholars Working Group

Supported by Robert Wood Johnson Foundation Quality Health Care Grant #043806
© 2007 Tom Ferguson, MD. This work is licensed under the Creative Commons Attribution-Noncommercial 3.0 License. To view a copy of this license, visit http://creativecommons.org/license/results-one?license_code=by-nc; or, (b) send a letter to Creative Commons, 543 Howard Street, 5th Floor, San Francisco, California, 94105, USA. • http://www.e-patients.net.

…[When patients] participate more actively in the process of medical care, we can create a new healthcare system with higher quality services, better outcomes, lower costs, fewer medical mistakes, and happier, healthier patients. We must make this the new gold standard of healthcare quality and the ultimate goal of all our improvement efforts:

Not better hospitals.
Not better physician practices.
Not more sophisticated electronic medical systems.
Happier, healthier patients.

—Charles Safran

Contents

Editors' Note and Dedication
Acknowledgements
Foreword by Lee Rainie and Susannah Fox
Preface
Introduction

1. Hunters and Gatherers of Medical Information
2. Content, Connectivity, and Communityware
3. Patient-Centered Networks: Connected Communities of Care
4. The Surprisingly Complex World of e-Communities
5. e-Patients as Medical Researchers
6. Learning from e-Patients
7. The Autonomous Patient and the Reconfiguration of Medical Knowledge

Afterword--2013 by Charles Smith, Terry Graedon, Joe Graedon, Alan Green, John Grohol, and Daniel Sands, in collaboration with the SPM Founders' group
About the Author
About the e-Patient Scholars Working Group

Editors’ Note and Dedication

We dedicate this work to the memory of our friend and colleague, Tom Ferguson, MD.

Before his untimely death, Tom assembled the group of advisors he dubbed the e-Patient Scholars Working Group. Several times over the years during which he was working on this White Paper, we would gather and participate in informal but intense seminars in which we would share research and ideas on the topics Tom proposed. We would read his latest version of the White Paper and give him feedback. We would exchange exciting ideas with each other and offer suggestions and encouragement for obstacles we faced in our own work. We did not expect, the last time we gathered with Tom at the Cooks’ Branch Conservancy outside of Houston, Texas, in February 2006, that he would not finish the White Paper.

We did not know that Tom’s 15-year battle with multiple myeloma would end just a few months later. When Tom died in April 2006, we grieved and vowed to finish the work to which he had dedicated his last years. We did not want his remarkable work and foresight to be lost. We continued to meet together as a group, dividing responsibility for the various chapters. Each one was assigned an editor and a reviewer. Then each chapter was read and edited a final time for continuity. We have done our best to review, fill in and assemble the pieces so that much of the work Tom did for the White Paper is represented here.

Last year as we all met together after his death, it became clear that beyond the White Paper, Tom was already thinking about some truly revolutionary concepts. The research to support or test his ideas has not yet been done, and he had barely begun to sketch them out on paper. We have only alluded to these more advanced theories in the White Paper and its Afterword. We trust that the topics we have included will offer the reader an excellent overview of the issues involved in exploring online health care in the information age.

Tom coined the term e-patients to describe individuals who are equipped, enabled, empowered and engaged in their health and health care decisions. He envisioned health care as an equal partnership between e-patients and the health professionals and systems that support them. We offer this definition of e-patient from Wikipedia to set the tone for the following work:

"e-Patients represent the new breed of informed health consumers, using the Internet to gather information about a medical condition of particular interest to them. The term encompasses both those who seek online guidance for their own ailments and the friends and family members who go online on their behalf. e-Patients report two effects of their online health research—'better health information and services, and different (but not always better) relationships with their doctors.'"

The e-Patient Scholars Editorial Team
Meredith Dreiss
Susannah Fox
Gilles Frydman
Joe and Terry Graedon
Alan and Cheryl Greene
John Grohol
Dan Hoch
Charlie and Connie Smith
March, 2007

Acknowledgements

Special thanks to my long-time teacher and mentor Lowell S. Levin, Professor Emeritus at the Yale School of Public Health, who suggested that doctors might not have all the answers. Thanks to Stewart Brand, Howard Rheingold, Kevin Kelly and their colleagues at the Whole Earth Catalog who invited me to review medical books and edit the self-care sections for a number of different Whole Earth publications. Thanks to Michael Castleman and Carole Pisarczyk, my partners at Medical Self-Care Magazine. I think we may have started something. And thanks to Ed Madara, director of the American Self-Help Clearinghouse, who invited me to look in on some of the early dial-up self-help bulletin boards nearly twenty years ago.

I owe a great debt of gratitude to my fellow members of the e-Patient Scholars Working Group for their willingness to join me in the difficult process of rethinking some of our most fundamental assumptions about doctors and patients, for walking so fearlessly and brilliantly into the unknown, and for providing a continuing supply of advice, encouragement, and support as this White Paper moved toward its final form. A struggling author could ask for no finer friends than these: Elie Anaissie, Lew Engle, Susannah Fox, Gilles Frydman, Joe Graedon, Teresa Graedon, Alan Greene, Cheryl Greene, Jan Guthrie, Daniel Hoch, John Lester, Ed Madara, Lee Rainie, Richard Rockefeller, Danny Sands, Charlie Smith, and Connie Smith.

And all e-patients—and their professional allies—owe more than we may realize to the creators of the Internet, Vinton Cerf, Leonard Kleinrock, Larry Roberts and others, to Tim Berners-Lee, who not only created the World Wide Web, but left it in the public domain, for all to use freely; to Berners-Lee, Esther Dyson, Pattie Maes, and their colleagues at ICANN and the Electronic Frontier Foundation, and other organizations who work to keep the net free, and the many nameless nerds who labor long and hard to make the Internet a safe and inviting place for those who are trying to do good.

The Robert Wood Johnson Foundation provided generous financial support for this project.

Last but not least, my wife, Meredith Dreiss, has offered me unstinting support and love throughout this long project. She has stood by me through my long illness and provided an opportunity for the “e-patient mafia” to gather regularly at Cook’s Branch Conservancy.

Foreword

By Lee Rainie and Susannah Fox

The Pew Internet & American Life Project

It gives us great pleasure to recommend this white paper—and its author—to everyone interested in understanding how our first generation of e-patients is slowly but surely transforming our healthcare system. For we have learned a great deal about the emerging e-patient revolution from its author, Tom Ferguson, and from his team of expert advisors and reviewers.

We first met Tom in November 2000, shortly after the release of the Pew Internet & American Life Project's first e-patient survey, *The Online Health Care Revolution: How the Web Helps Americans Take Better Care of Themselves*.[i] Tom had addressed many of the same topics in his book, *Health Online: How to Find Health Information, Support Groups, and Self-Help Communities in Cyberspace*,[ii] so he was asked to critique our report—while Lee was invited to defend it—on a live National Public Radio call-in talk show.

But if the producers had expected to trigger a battle between competing author-researchers they were badly disappointed, for Tom was generous and enthusiastic in his praise, calling our report "the most important study of e-patients ever conducted," and insisting that our conclusions were exactly in line with his own observations:

- **93 percent of e-patients said that it was important that the Internet made it possible to get the medical information they needed *when it was most convenient for them*.**
- **92 percent said that the medical information they found was useful.**
- **91 percent of e-patients looked for information on a physical illness.**
- **83 percent of e-patients said that it was important that they could get more health information online then they could get from other sources.**
- **81 percent said that they learned something new.**
- **80 percent of e-patients visited multiple medical sites. A few visited 20 sites or more.**
- **72 percent of e-patients searched for medical information just before or after a doctor's visit.**
- **70 percent said that the information they found influenced their medical decisions.**
- **51 percent said that online information had affected the way they are.**
- **53 percent of e-patients were looking for medical information for a loved one, while 43 percent were looking for themselves.**

When the program ended, they continued their conversation by phone. Tom came to our offices in Washington DC a few days later, and the three of us talked intently for several hours. Before he left, we invited him to join our Pew Internet health research team. So we were not surprised to hear that when the Robert Wood Johnson Foundation decided to explore the experiences of our first generation of e-patients, they turned to Tom for help.

The project design they came up with was most ingenious: Tom wanted to assemble a team of leading researchers, developers, and visionaries in the emerging field of e-patient studies. These advisors would help him frame the questions to be explored. The advisors—and an even larger group of expert reviewers—would then review and critique successive drafts of each chapter. This document thus reflects the perspectives of the many diverse collaborators Tom has brought together, sometimes virtually and sometimes physically, to consider these vital topics.

We have been honored to play some modest part in this process, serving as editors and co-authors of Chapter One, which reviews the results of our first five years of e-patient surveys, and reviewing and commenting on many of the other chapters. And we have been lucky enough to get the chance to meet and work with many of Tom's other collaborators.

In the pages that follow, you will find a uniquely helpful overview of the contemporary e-patient revolution. Tom and his colleagues suggest that this massive, complex, unplanned, unprecedented, and spontaneous medical empowerment of our lay citizens may turn out to be the most important medical transformation of our lifetimes. The world of the e-patient as they portray it is not a dry, impersonal realm of facts and data, but a living, breathing interactive world in which growing numbers of our fellow-citizens—both patients and professionals—interact in highly personal and fully human ways.

Tom and his colleagues have done us all a further service by combining their detailed and compelling portrait of the emerging e-patient phenomenon with an unusually original and creative set of tentative suggestions for leveraging the most promising developments from the e-patient experience in the most suitable, sustainable, and productive ways.

This white paper offers a variety of helpful perspectives on the e-patient experience. There is something for everyone:

Clinicians will be reassured to find that many of our early fears about the potential dangers of patients using the Internet have been proven largely groundless. They will learn a great deal about collaborating with their own e-patients. And those who have [iii]resisted or discouraged

their patients' use of the Internet will find a compelling explanation of the benefits of becoming more "Net-friendly."

Medical executives, policymakers, employers, insurance executives, government officials, and others seeking solutions to our most troublesome and intractable healthcare problems will find an unprecedented array of powerful and promising solutions for sustainable healthcare improvement and reform.

e-Patients and potential e-patients—and this means all of us—will find confirmation of our online experiences, inspiring stories of what other e-patients have accomplished, and suggestions of even more sophisticated ways we might use the Internet to obtain the best possible medical care for ourselves and our loved ones.

Researchers like ourselves will find many fascinating new hypotheses and questions to consider: How is it that the groups of *expert amateurs* Tom and friends describe are able to provide their members with such valuable medical help? To what extent can shared experiences and advice from patient-peers supplement or replace the need for professional advice and care? Now that anyone with a modem can publish information, what new quality practices and resources do e-patients use to avoid problems and stay out of trouble? What will be the consequences of turning the previous century's old *doctor knows best* model of medical information flow upside down? How often and in what cases do *patients* actually know best? Might it eventually be those wise clinicians and patients who collaborate via the new models presented here who know the most of all? And at the pinnacle of this research mountain sits an even more profound question: Are these technologies changing our traditional patterns of social connection so radically that we may need to begin thinking about our first generations of online humans as *homo connectus?*

Tom and his colleagues understand that the conclusions they offer here are not the final word on these topics. As they repeatedly suggest, additional perspectives, more innovative research, and further demonstration projects are badly needed. And they intend to make this a living document, inviting readers with ideas, comments, or additional research agendas to join with the current collaborators in imagining, and in continually updating, a vision of our common healthcare future.

The Robert Wood Johnson Foundation staffers who invited Tom and his team to prepare this document should be proud to have helped create such a valuable resource and roadmap. May the chapters that follow prove as useful to you as they have already been for us. We hope you

will review its conclusions, consider its recommendations, and then join us via the projects' new weblog, www.e-patients.net.

---

[i] Susannah Fox and Lee Rainie, *The Online Health Care Revolution: How the Web helps Americans take better care of themselves*, Pew Internet & American Life Project, Washington DC, Nov. 26, 2000. http://www.pewinternet.org/PPF/r/26/report_display.asp (Accessed Aug. 6, 2004).

[ii] Tom Ferguson, *Health Online: How to Find Health Information, Support Groups, and Self-Help Communities in Cyberspace,* (Reading, MA: Addison-Wesley/Perseus Books, 1995).

The time has come for a major conceptual shift…
From viewing lay people as consumers of health care
to seeing them as they really are: its primary providers.

—Lowell Levin & Ellen Idler

Preface

In his groundbreaking work, Thomas Kuhn identified two contrasting types of scientific work.[1] The first, *normal science,* involves the gradual accumulation of knowledge within a dominant professional paradigm that is still timely and effective.[2] The discovery of the structure of DNA is one example of *normal science*: the investigators knew what they were looking for, knew the methods they would need to use, had faith and trust in their underlying paradigm, and immediately knew it when they found the answer.[3] A healthy and vital professional paradigm can be a sort of unifying cultural DNA, helping members of a profession work together.

But there can be a dark side to professional paradigms as well. Since observations, approaches, and strategies that don't conform to the accepted tenets of the dominant

paradigm are typically ignored, denied, or explained away, an outdated paradigm can insulate a professional community from new developments that are "off the radar screen" of their customary ways of thinking. And the understandable professional impulse to defend and protect a valued paradigm can lead professionals to champion obsolete practices, to stifle needed change, and to make attempts at innovation—within the paradigm—unproductive.

Outdated professional paradigms thus sometimes persist far longer than they should. And so in times of rapid technological or cultural change, when it becomes increasingly clear that the dominant professional paradigm in its traditional form no longer serves the needs of the greater community, a second type of scientific work becomes necessary.

Disruptive science seeks to identify and understand the underlying limitations of the old paradigm, and to facilitate the development of a more suitable and sustainable new scientific worldview. And while the practitioners of *normal science* may keep their methods private and conceal their results until such time as it best serves their purpose, practitioners of *disruptive science* are typically more open and collaborative in their efforts, reaching out to a network of like-minded investigators, seeking their advice and inviting their collaboration. The shift from the Ptolomaic to the Copernican model of the solar system, as described by Kuhn,[4] provides a valuable illustration of disruptive science.

Many of the insights in the chapters that follow came directly from the thousands of e-patients with whom my colleagues and I have had the honor of communicating over the past decade and more. This includes thousands of free-text responses to e-patient surveys conducted through the Pew Internet & American Life Project. Additional e-patient insights were passed on via my extraordinary group of advisors, the e-Patients Scholars Working Group. These invaluable colleagues have collaborated and brainstormed and debated and reviewed many earlier drafts. Together and separately, we have visited e-patient weblogs and home pages, participated in e-patient support groups, subscribed to e-patient mailing lists, and lurked in e-patient chat rooms and Web forums. So in addition to summarizing the opinions of some of the most knowledgeable professionals and researchers, I have tried, whenever possible, to let the reader hear the contemporary e-patient's voice.

As my colleagues and I journeyed through this rapidly evolving and largely uncharted domain, we tried to imagine a future healthcare system in which the many positive trends and developments we have encountered could be accepted, acknowledged, supported, and

integrated with our current patterns of medical practice. I am immensely grateful to all who helped with this project, and I have relied heavily on their wisdom and guidance in preparing the pages that follow. The members of the e-Patient Scholars Working Group are listed separately.

My role in this project has been much like that of a host at a dinner party, inviting a remarkable cast of characters to share their insights and describe their visions. I have listened carefully and have asked innumerable questions. I've done my best to synthesize what I have heard, and what I myself discovered, into an intelligible conclusion:

> **e-Patients are driving a healthcare revolution of major proportions.**
>
> **The old Industrial Age paradigm, in which health professionals were viewed as the exclusive source of medical knowledge and wisdom, is gradually giving way to a new Information Age worldview in which patients, family caregivers, and the systems and networks they create are increasingly seen as important healthcare resources. But the emerging world of the e-patient cannot be fully understood and appreciated in the context of pre-Internet medical constructs.**
>
> **The medical worldview of the 20th century did not recognize the legitimacy of lay medical competence and autonomy. Thus its metrics, research methods, and cultural vocabulary are poorly suited to studying this emerging field. Something akin to a system upgrade in our thinking is needed—a new cultural operating system for healthcare in which e-patients can be recognized as a valuable new type of renewable resource, managing much of their own care, providing care for others, helping professionals improve the quality of their services, and participating in entirely new kinds of clinician-patient collaborations, patient-initiated research, and self-managed care.**
>
> **Developing, refining, and implementing this new open-source cultural operating system will be one of the principal challenges facing healthcare in the early decades of the 21st century. But difficult as this task may prove to be, it will pay remarkable dividends. For given the recognition and support they deserve, these new medical colleagues can help us find sustainable solutions to many of the seemingly intractable problems that now plague all modern healthcare systems.**

We offer this white paper as a series of preliminary notes on this new operating system.

—Tom Ferguson, M.D.
Austin, Texas, January 2006

---

[1] Thomas S. Kuhn, *The Structure of Scientific Revolutions (First ed.)*. Chicago: University of Chicago Press, 1962.

[2] A medical paradigm is a predetermined professional framework that specifies the appropriate role of the members of the profession, the role of those they care for, they type of work to be done, they types of problems to be studied, and the appropriate methods to be used.

[3] Thomas S. Kuhn, *The Road Since Structure.* Chicago: University of Chicago Press, 2000, pp. 165-177.

[4] Ibid., pp. 165-177.

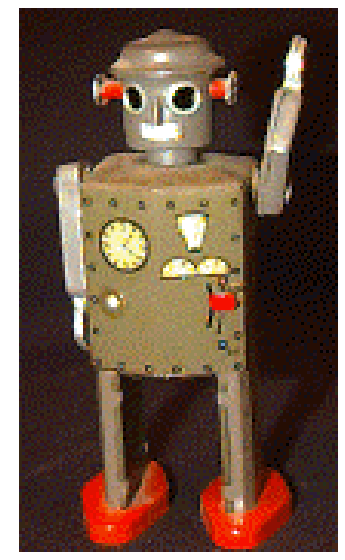

Atomic Robot Man,
5" high, Japan, 1948

The key question we must ask is not
what technology will be like
in the future, but rather,
*what **we** will be like…*
—Sherry Turkle

Introduction

I collect old toy robots. My *Atomic Robot Man* robot (Japan, 1948), shown above, is a personal favorite. For many years I didn't understand the powerful hold these dented little metal men maintained on my imagination. One day I finally got it: They show us how the culture of the 40s and 50s imagined the future. Cast-metal humanoid automatons would do the work previously supplied by human labor.

That wasn't how things turned out, of course. By making more powerful and productive forms of work possible, our changing technologies made older forms of work unnecessary. So instead of millions of humanoid robots laboring in our factories, we have millions of information workers sitting at computers. We didn't just automate our earlier forms of work. It was the underlying nature of work itself that changed.[i]

In much the same way, we've been projecting the implicit assumptions of our familiar 20th century medical model onto our unknown healthcare future, assuming that the healthcare of 2030, 2040, and 2050 will be much the same as that of 1960, 1970, and 1980. But bringing healthcare into the new century will not be merely a matter of automating or upgrading our existing clinical processes. We can't just automate earlier forms of medical practice. The underlying nature of healthcare itself must change.

This is not some technoromantic vision of an impossibly idealist future. It is already happening. The changes are all around us. As we will see, the roles of physicians and patients are already

changing. And our sophisticated new medical technologies are making much of what the physicians of the 1950s, 1960s, and 1970s thought of as *practicing medicine* unnecessary. Financial constraints are making the old-fashioned physician's role unsustainable. And millions of knowledge workers are emerging as unexpected healthcare heroes.

When they, or a loved one, become ill, they turn into e-patients—citizens with health concerns who use the Internet as a health resource, studying up on their own diseases (and those of friends and family members), finding better treatment centers and insisting on better care, providing other patients with invaluable medical assistance and support, and increasingly serving as important collaborators and advisors for their clinicians.

We understand that this document may raise more questions than it answers. And while we are by no means ready to dot all the I's or cross all the T's, we strongly suspect that the principal protagonist of our next-generation healthcare system will not be a computerized doctor, but a well-wired patient. Yet our formal healthcare system has done little to recognize their accomplishments, to take advantage of the new abilities, or to adapt itself to their changing needs.

Turning our attention to this promising and fertile area—which to date has somehow remained *off the radar screens* of most health policymakers, medical professionals, federal and state health officials, and other healthcare stakeholders—may be the most important step we can take toward the widely-shared goal of developing a sustainable healthcare system that meets the needs of all our citizens. But as the battered little robot beside my computer constantly reminds me, we are in the early stages of this process. And our current and future new technologies may change the nature of healthcare in ways we can, as yet, only vaguely imagine.

As MIT's Sherry Turkle has suggested, instead of asking how these new technologies can help us make the familiar processes of medical care more efficient and effective, we should ask ourselves how these new technologies are "...changing the ways we deal with one another, raise our children, and think about ourselves? How are they changing our fundamental notions of who we are and what we need to do and who we should do it for? What new doors are they opening for us?" [ii]

The key question we must ask, Turkle suggests, "…is not what technology will be like in the future, but rather, what will ***we*** be like…" when we have learned to live and work appropriately within the new technocultural environments even now being created by our new technologies.

For the healthcare of the future—if it is to survive—will be as novel and unexpected to those of us trained as clinicians in 20th century medicine as today's computer-toting knowledge workers would have been to the social planners of the 1940s and 50s. We hope that the chapters that follow provide our readers with some interesting and useful perspectives on these questions.

---

[i] The term "knowledge worker" was first introduced by Peter Drucker in his book, *The Effective Executive* (1966). For an excellent overview of the rise of the knowledge worker in western society, see: Peter F. Drucker, "Knowledge Work and Knowledge Society: The Social Transformations of this Century, The 1994 Edwin L. Godkin Lecture, presented May 4, 1994, the John F. Kennedy School of Government, Harvard University.

[ii] Anonymous, "An Interview with: Professor Sherry Turkle," *Open Door: Ideas and Voices from MIT*, MIT Alumni Association, July/August 2003. Available at http://alumweb.mit.edu/opendoor/200307/turkle.shtml. (Accessed on March 8, 2005)

Men are suddenly nomadic gatherers of knowledge... informed as never before... involved in the social process as never before... [as] we extend our central nervous system globally...

—Marshall McLuhan

CHAPTER ONE

Hunters and Gatherers of Medical Information

One morning in 1994, the year Netscape released the first commercial Web browser, the Englewood Hospital library in Englewood, New Jersey, received a most unusual call. The caller identified himself as Dr. Harold Blakely, a local family practitioner. He gave the librarian a bibliographic citation for an article in a medical journal and asked her to make him a copy and to leave it on the table outside the library door, where he could pick it up on his evening rounds.[1] This request was not unusual. The hospital librarians frequently left copies of journal articles that local doctors could pick up after the library had closed.

Later that afternoon, the caller phoned again, checking to be sure that his article was ready. But the library's director, Kathy Lindner, took the call this time. Ms. Lindner knew Dr. Blakely. But she did not recognize the caller's voice. After a brief discussion with a colleague, she phoned Dr. Blakely's office. After several minutes a bewildered Dr. Blakely came to the phone. He assured Ms. Lindner that neither he nor anyone in his office had called the hospital library that day.

Half an hour after the library closed that evening, a nervous, well-dressed man with carefully barbered grey hair entered the hospital through a side entrance. Walking with a cane, he passed the elevator, climbed the stairs with some difficulty, and continued down the second floor hallway toward the medical library. As he picked up the envelope with Dr. Blakely's name on it, a hospital security guard stepped out of the doorway where he had been waiting and asked him to identify himself.

Under the questioning of the hospital's security service, he admitted that he was Edwin Murphy, a 58-year-old insurance agent with a chronic hip problem. Dr. Blakely, his physician, had been urging him to undergo a promising new surgical procedure. Mr. Murphy was intrigued but not convinced. He wanted to know more about the potential risks and benefits of the proposed procedure and had repeatedly asked Dr. Blakely to help him obtain a copy of the definitive review article, which had recently appeared in a major medical journal. In spite of his repeated requests, Dr. Blakely had not done so. Finally, in desperation, Mr. Murphy had

decided that there was only one way to obtain this vital medical information he needed: He would have to impersonate his own physician.

"An Unusual Sloshing Sound Inside her Head"

Fast-forward to five years later. Marian Sandmaier's 16-year-old daughter, Darrah, had been experiencing severe headaches. Her family pediatrician took her vital signs, peered into her eyes, checked her balance and her reflexes, and then shrugged her shoulders, pronouncing the young woman more-or-less healthy. "It may just be sinuses," the doctor observed.

"But what about the pain in her neck? And that odd sound she's been hearing?" Darrah's mother wanted to know. As they had just been explaining, Darrah's headaches had been accompanied by an unusual sloshing sound inside her head. The doctor paused at the exam room door. She shrugged again. "Hard to say," she said evenly. "Call back in two weeks if she's still having problems."

But unlike the unfortunate Mr. Murphy, Marian Sandmaier knew how to use the Internet. And the next day, as she sat down at her computer, she remembered that Darrah had recently started taking an antibiotic for a tenacious skin problem. She logged onto Google and typed in "minocycline" and "side effects." And before she knew it, she was reading the obscure medical details of a rare side effect called *pseudotumor cerebri,* an accumulation of fluid around the brain that can produce severe headaches, neck pain, and vision problems. One of the symptoms of this rare condition, as she soon learned, is a distinctive and unusual whooshing, sloshing, or roaring sound heard inside the head. Reading further, she discovered that pseudotumor cerebri can damage the optic nerve, producing severe vision difficulties and, in some cases, blindness.

When she had recovered from her initial shock, Sandmaier walked slowly and carefully downstairs. She asked Darrah to stop taking the antibiotic. She then told her husband what she'd learned, handing him a thirty-page printout of her online research. He took Darrah back to their dermatologist, who had prescribed the suspect medication, the next day. The dermatologist dismissed Sandmaier's diagnosis, but she switched Darrah to a new antibiotic nonetheless. When they got home, Sandmaier checked its side effects on Google. The list included pseudotumor cerebri.

> **Two doctors had now shrugged their shoulders at my daughter's symptoms. One wrote it off as a garden-variety headache, without ever asking if Darrah was on any medication. The other had dismissed our fears, then prescribed another drug known to cause the very same disorder. And through it all, our daughter's symptoms continued to worsen.**

> **Yet who was I to diagnose a rare disorder—on the Internet, no less? These two physicians had 30 years of clinical experience between them. All I had was a tall stack of Web printouts and a passion for my child's health. And this was a rare disease, I reminded myself. Rare means hardly anybody gets it. Then another voice within me rose up, urgent, unbowed. *But it means some people do.***
>
> **I called Darrah's dermatologist and pediatrician and told them that I had rejected their diagnoses. With the help of the Internet, I had made my own tentative diagnosis and would proceed accordingly. When I said the words *pseudotumor cerebri*, they both became very quiet.[2]**

Sandmaier took Darrah to a top neuro-ophthalmologist at the University of Pennsylvania, saying nothing about her suspicions. After a lengthy battery of neurological tests and exams, the specialist announced her verdict.

"Your daughter is experiencing a rare side effect called pseudotumor cerebri," she explained. She went on to describe how the buildup in cerebral fluid sometimes reverses itself—if the patient stops taking the toxic drug soon enough. This was apparently what had happened in Darrah's case. In the weeks and months that followed, the girl's symptoms slowly subsided and finally disappeared. Her mother's quick action may have saved her sight.

For e-patients like Marian Sandmaier, the traditional role of unknowing, uncomplaining, passive patient has gone the way of the horse and buggy. She and her e-patient colleagues now routinely turn to the Internet when a medical need arises. They use it to prepare for and follow up on doctor visits. They use online health resources to supplement and double-check the information and guidance their clinicians are able to offer within the constraints of the typical time-pressured doctor-patient encounter. They go online to explore treatment options their clinicians did not mention, to double-check their diagnoses, to learn about alternative or complementary treatments, to compare the treatments their doctors suggest with those recommended by other patients at other treatment centers, and to compare their current clinicians with other providers. Marian Sandmaier is an exemplary but not unusual member of our first generation of e-patients. e-Patients have become an important new resource for healthcare quality. And they now routinely communicate with their clinicians from a newfound position of knowledge, competence and power.

Marian Sandmaier is a medical journalist who served as a reviewer for this White Paper. Her story of online medical sleuthing, from which the preceding summary was adapted (with the author's kind permission), appeared in the *Washington Post.*[3] It won the 2004 American Society of Journalists and Authors Award for Essay Writing.[4]

Turning to Dr. Google

As of April 2006, 73% of American adults, or about 147 million people, have access to the Internet—a 63 percent increase from the 90 million American adults who had access in 2000.[5] Eight in ten internet users have searched for information on at least one of sixteen health topics. A 2002 survey by the American Academy of Dermatology found that patients who needed information about a new medical procedure were *twice* as likely to go online as to ask their physician.[6] If we include teens and children—who turn out to be active users of online health resources[7]—the ranks of US e-patients now exceed 122 million.

Much of what we know about e-patients comes from an ongoing series of in-depth e-patient surveys my colleagues and I have conducted at the Pew Internet & American Life Project.[8] The health reports, published over the last seven years, provide a good deal of underlying data to support the observations, ideas, trends, and conclusions offered here.[9]The free online newsletter *Health Care News*, published by Harris Interactive since 2001, has been another valuable resource.[10] Recent studies in the *Journal of Medical Internet Research*, the *Journal of the American Informatics Association*, the *International Journal of Medical Informatics*, and the British journal, *Health Expectations*[11] have provided additional insights. Other key sources are listed in the Author's Notes.[12] But here is a brief summary of what we know so far:

- **When turning to the Internet for health and medical information, most e-patients operate as "hunter-gatherers," using search engines to find the resources they seek—the "Dr. Google" phenomenon. Here are the kinds of information they search for most frequently:[13]**

Health Topics Searched Online

In all, eight out of ten American Internet users have searched for information on at least one major health topic online. Many have searched for several kinds of information.

Health Topic	Internet Users Who Have Searched for Info (%)
Specific disease or medical problem	66%
Certain medical treatment or procedure	51
Diet, nutrition, vitamins, or nutritional supplements	51
Exercise or fitness	42
Prescription or over-the-counter drugs	40
Health insurance	31
Alternative treatments or medicines	30
A particular doctor or hospital	28

Depression, anxiety, stress, or mental health issues	23
Experimental treatments or medicines	23
Environmental health hazards	18
Immunizations or vaccinations	16
Sexual health information	11
Medicare or Medicaid	11
Problems with drugs or alcohol	8
How to quit smoking	7

Source: Pew Internet & American Life Project November 2004 Survey (N=537). Margin of error is plus or minus 4%.

• Age is an important factor. Younger citizens use online health resources more frequently than their older counterparts.[14] Of those aged:

18 to 29	82 percent have used online health resources
30 to 39	68 percent have used online health resources
40 to 49	63 percent have used online health resources
50 to 64	49 percent have used online health resources
65+	26 percent have used online health resources

• Baby Boomers currently aged 50 to 64 are dramatically more active and assertive as e-patients than those currently aged 65 to 82 (the Silent Generation).[15] Thus as tomorrow's seniors develop an increasing number of health concerns, they will take a much more active role in managing their own medical care. Compared to the members of our current generation of seniors, current pre-seniors are:

• Five times as likely to go online to seek consumer information about a doctor, hospital, or nursing home, to search for mental health information, or to visit WebMD.com

• Four times as likely to have searched for information on alternative or experimental treatments

• Three times as likely have searched for information on cancer, diabetes, nutrition, exercise, weight issues, or medical products.

• Three times as likely to say that they get “a lot” of health and medical information from online sources.

• Gender is also a factor. Women are more likely to search for specific diseases and other medical problems, reflecting their traditional role as family health caregivers. Men are more likely to search for information relating to sexual health, drugs, alcohol, and smoking.

• Expertise increases with use. As our first generation of e-patients becomes more experienced with the Internet, they are becoming more proficient and capable power users, more skilled at searching, more likely to use e-mail for serious purposes such as seeking or providing health advice. They are also more likely to post online content for other patients.[16]

Three Categories of e-Patients

In their insightful 2000 report, “Health e-People: The Online Consumer Experience,”[17] Cain, Sarasohn-Kahn, and Wayne identified three types of e-patients—The Well, The Newly Diagnosed, and The Chronically Ill and Their Caregivers. We at the Pew Internet Health team

adapted this model to reflect our observations, combining the newly-diagnosed with those who are dealing with a complication or reversal for an established diagnosis and including caregivers in all three groups. We currently divide the e-patient population as follows: (1) *The Well and their Caregivers* ("The Well"); (2) *Those Facing a New Medical Challenge and their Caregivers* ("The Acutes"); and (3) *Those with Chronic but stable illnesses and their Caregivers* ("The Chronics").

> **The Well** About 60 to 65 percent of e-patients are well. Most members of this group think about their health only occasionally and sporadically.
>
> *How they use the Internet* — While some may "keep an eye out" for particular health-related concerns, e.g., exercise, stress, yoga, nutrition, most, as Cain and colleagues suggest, will occasionally browse for general health and wellness information in much the same way as they might look for news or feature stories or financial information. They may occasionally look up pertinent medical information online before or after a doctor's visit—either their own or that of a loved one. They are the lightest and least frequent users of online health resources.
>
> **The Acutes** About 5 to 6 percent of e-patients are currently facing a new medical concern or challenge. Some have recently developed a relatively minor medical condition, e.g., acne, upper respiratory infection, or bladder infection. Others have received a new diagnosis of a more serious condition, e.g., AIDS/HIV, cancer, or diabetes. Some have had a recurrence of a condition that has not been bothering them for a while. Still others are facing a challenging new development for a previously diagnosed condition, e.g., their previous treatment is no longer working, they are experiencing a troublesome side effect or other treatment complications.
>
> *How they use the Internet* — They search online resources intensively, casting a wide net for online information, recruiting others to help, reaching out to professionals and patients they have found online, participating in online medical discussions and support communities, and asking online consultants, medical librarians, and medical search services to help them find what they are looking for. They are the heaviest users of online health resources.
>
> **The Chronics** About 30 to 35 percent of e-patients have one or more stable chronic illnesses, but are not currently dealing with a pressing short-term medical challenge.
>
> *How they use the Internet* — They use online health resources on a regular basis to manage their illnesses and to help them keep up to date on their conditions. They may look up pertinent information online before or after a doctor's visit. They may communicate with other patients and caretakers concerned with the same disease, either via online support communities or face-to-face local support groups. And they use e-mail to keep in touch with their own private networks of family members and friends. They are moderately heavy users of online health resources.

Many Acutes use the Internet every day. Many Chronics use it several times a month. Most of the Well use it much less frequently, as illustrated by the following answers to a May 2002 Pew Internet & American Life Project report:[18]

How often do you use the Internet to look for advice or information about health or health care?

**Every Day**	**4 percent**
**Several times a week**	**13 percent**
**Several times a month**	**25 percent**
**Every few months**	**43 percent**
**Less often**	**15 percent**

The circle graphs below demonstrate a model for e-patient traffic on the Net, based on discussions with experts in the field. It is clear that not all e-patients are created equal. A relatively small population of Acutes probably accounts for half or more of all e-patient Internet traffic, while the Well, even though they constitute a majority of e-patients, account for a relatively small portion of online health traffic.

The e-Patient Population **Their Total Net Traffic**

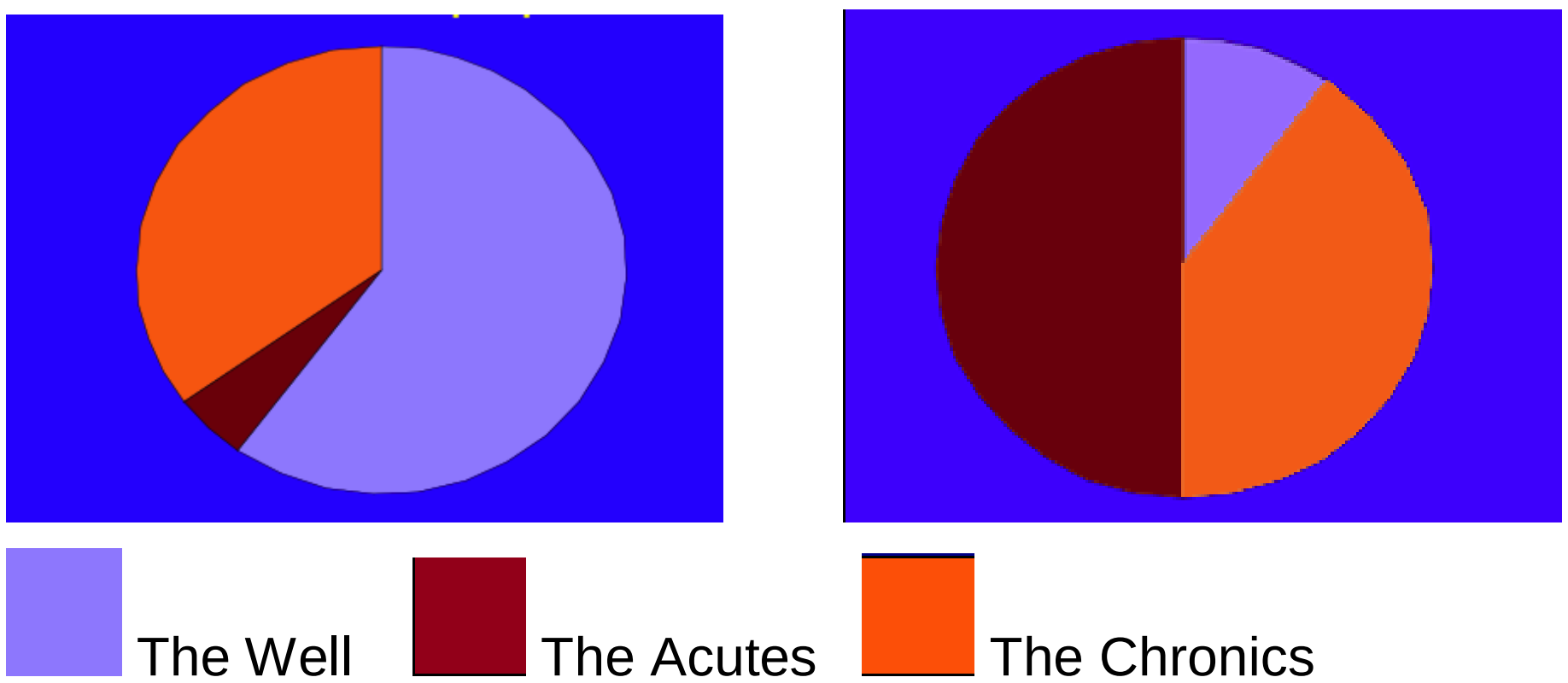

We have found this three-part division particularly helpful when attempting to interpret the results of e-patient surveys, for it can help us understand and appreciate the diversity and complexity of the emerging world of online health resources and the role they play in the lives of contemporary citizens. For example:

- The Well, especially women, often serve as managers for other family members' health concerns. So even though they are only occasional users of online health resources, having access to the Internet makes it possible for them to serve as more effective monitors and caregivers for their family's health concerns. As one e-patient recently noted:

  > **My mother-in-law suddenly began bruising very badly. Medical personnel simply said it was because she was old. I was not satisfied with this answer because the onset was so sudden. Turning to the Internet, I found that it was because her aspirin dosage was too high." After checking with her clinician, this e-patient advised the patient to lower her aspirin dose. Her bruising problems soon disappeared.**[19]

- In addition to managing their own medical conditions, many Chronics spend a great deal of time caring for others. They are often the most active participants in online support communities, where they frequently serve as hosts, advisors, "community elders" and "big brothers/big sisters" to the newly diagnosed. And Chronics are typically the most likely to develop websites for their condition, to post content designed to help others, and to respond to the questions of other patients.

- Since their numbers are so small, Acutes can be easily overlooked in surveys of the general e-patient population. Yet Acutes often play a much more important role in generating total Internet traffic (Web searching plus participation in online communities plus health-related e-mail) than their limited numbers might suggest. A broad-based survey might suggest that the "average" e-patient spends about thirty minutes and

visits only two to five different websites on a typical health search.[20] But personal accounts by Acute e-patients suggest that exhaustive searches extending over dozens or hundreds of hours and many days or weeks are the rule rather than the exception When a 35-year-old e-patient named Jack learned that he had tested positive for AIDS/HIV, he devoted himself to an intense series of "wildly generic searches" in an effort to learn everything he could about the condition.[21] In one of his early sessions, he found several newsgroups where patients discussed his condition:

> **I went through and pulled a list of everything that had to do with HIV… I went through each newsgroup and—this is kind of embarrassing—sat there for three days and read every single post. There were 2,476 of them. I read them all.**

- Many Acutes and Chronics use the Internet in considerably more sophisticated ways than many health professionals—and online health researchers—had realized.[22] They use e-mail, especially the mailing lists, heavily, but they also use bulletin boards, chat rooms, instant messages and access to medical journals. In the course of their online activity, they may also arrange face-to-face meetings with individuals or support groups.
- e-Patients often perform several different health-related activities within the same online session. When White Paper advisors John Lester and Dan Hoch and their colleagues at Massachusetts General Hospital asked members of an online support community for neurological conditions (Braintalk) what they had done in their last health-related online session, they found the following:[23]

**Searched for information only**	**50 percent**
**Searched for and posted information**	**27 percent**
**Searched, posted and interacted with other e-patients**	**13 percent**
**Interacted with an online healthcare provider only**	**4 percent**
**Posted information only**	**4 percent**
**Searched, posted, and interacted with provider**	**2 percent**

The Accepting, the Informed, the Involved, and the In Control

Carina Von Knoop and her colleagues at the Boston Consulting Group have proposed another extremely insightful and useful e-patient typology. According to their model, an e-patient's empowerment level is related to two independent factors—the *severity of their condition* and their *attitude toward their physician*:

[24]

Group I: The Accepting ("doctor-dependent and uninformed") consider their doctors the ultimate medical authorities. They thus rely entirely or almost entirely on their clinicians for medical guidance. They feel that it is their doctor's prerogative to provide any medical information they need and to make all their medical decisions on their behalf. They are comfortable with the traditional paternalistic medical model. Even though they may have Internet access, they rarely go online to seek health information or guidance.

Group II: The Informed ("doctor-dependent but informed") also consider the physician the unquestioned leader of the healthcare team. They rely on clinicians to make many of their medical decisions for them. But they are much more likely to go online to learn more about their condition and its treatments, often doing so just before or just after a doctor's visit. They are occasional users of e-health resources and appreciate the fact that they can find the medical information they seek without "wasting the doctor's time with their questions."

Group III: The Involved ("junior medical partners") regard themselves as respectful junior members of their healthcare team, fairly well informed and somewhat involved. While most would prefer to make their medical decisions jointly, in collaboration with their providers, they are not particularly assertive in standing up to their physicians, and will usually allow the doctor to control the interaction. If they disagree, with their doctor, they may or may not voice their position. And they will usually defer to their physicians' judgment.

Group IV: The In-Control ("autonomous patients") believe in making their own medical choices and will often insist on managing their own medical tests and treatments in the ways they think best, even if their clinicians disagree. They may help (or attempt to help) their clinicians keep up to date on new medical treatments and studies. They may start, manage, or contribute to local support groups, online patient communities, blogs, and other condition-specific websites. They often help other patients, provide feedback (both positive and negative) for their clinicians and treatment centers, and attend meetings and seminars where they can network with the top clinicians and patients from the top treatment centers for their condition.

Von Knoop and her colleagues found that dealing with a continuing or increasingly severe illness tends to drive e-patients from a medically passive to a medically autonomous role. As a person's medical condition becomes more severe, patients and caregivers tend to develop a higher level of medical knowledge, competence and control, adopting a more assertive and autonomous style in their interactions with their clinicians. In fact, patients who must see their physicians a dozen or more times a year are much more likely to be autonomous in their attitudes and online behaviors rather than doctor-dependent. These investigators also reported consistent and predictable variations in proactive vs. passive behavior across different diagnoses: e.g., compared to *allergy* patients, *muscular sclerosis* patients were six times as likely to exhibit behavior patterns characteristic of the In-Control group.

Internet Use by Specific Patient Populations

Recent studies of Internet use by specific clinical populations of patients suggest that many patients who seek professional medical services also make use of online health resources. A 2002 British study by Tuffrey and Finlay found that 22 percent of the parents in the waiting rooms of several local pediatric outpatient clinics had already done an online search for the problem for which their child was being seen that day, and many had gone far beyond the basics. Percentages of patients who had obtained more information on the following topics were as follows:[25]

**Profile of the diagnosed condition**	**89 %**
**Other medical treatments**	**66 %**
**Long term outcomes**	**60 %**
**Alternative/complementary treatments**	**41 %**
**Other possible diagnoses**	**40 %**
**Self-help or patient support groups**	**40 %**
**Other families with a child with a similar problem**	**28 %**
**Other doctors and hospitals specializing in the condition**	**22 %**

Tuffrey and Finlay also found that recommendations by clinic staff could be dramatically effective in increasing patients' use of the Internet: 67 percent of parents whose health professionals suggested they search the Internet for information on their child's condition had done so, versus only 20 percent of those whose clinicians had made no such suggestion.

A survey by James Metz and colleagues at the University of Pennsylvania found that among patients at radiation oncology centers in the Philadelphia area, the percentage of patients who had used the Internet to find cancer-related information ranged from 42 percent of patients at an academic medical center to 25 percent at community clinics to 5 percent at the local VA hospital.[26]

A 2002 study by Catherine Ikemba and colleagues at the Baylor College of Medicine found that among families at a Houston clinic specializing in the surgical repair of congenital heart defects in children, 58 percent had used the Internet to research their child's medical condition. Of those who had done so, 95 percent characterized the information they found as "helpful" or "very helpful" in understanding and caring for their child's problem. Eighty-two percent said that finding the information they needed was "easy," and 6 percent had developed websites specifically related to their child's congenital heart defect.[27]

Studies by Sue Ziebland and colleagues[28] describe how patients with cancer use the Internet "to gain, maintain, and display familiarity with a remarkable body of medical and experiential knowledge about their illness," thus increasing their sense of self-efficacy, developing effective coping skills in the face of serious illness, and presenting themselves as technically proficient and discriminating users of medical information and professional services. The following comment, from a 50-year-old man diagnosed with testicular cancer ten years before the interview, illustrates how dramatically the Internet has changed the experience of receiving a cancer diagnosis:

> **We have one very big advantage [today]… Now I use Yahoo… You type in testicular cancer, and I guarantee you that for the next two weeks you'll be looking at [a remarkable variety of sites with information on your condition, and will discover that] every site… is different. You have people's experiences, you have drug information, and you'll be able to read papers that are published on the Web by some of the most eminent doctors around. There is no excuse these days—if you haven't got a computer go down to an Internet cafe—there is no excuse whatsoever for not finding out about testicular cancer or all the other things... The information technology breakthrough [and] having the Internet available is just unreal. …we're very lucky because we're at the dawn of something that is quite remarkable.**

What e-Patients Like Best—and What They Say Is Lacking

Most e-patients appear to be quite satisfied with most aspects of their experiences using online health resources:

- **82 percent frequently found the information they are looking for online**
- **61 percent said that online information improved the way they take care of themselves[29]**

A significant percentage of e-patients said that they would have liked certain desired services or features they did not find online:[30]

- **Doctor-patient e-mail**
- **How to determine the correct tests and treatments**
- **More in-depth information about the quality of care provided**
- **The ability to schedule doctor appointments online**
- **Direct Internet access to online doctors.**
- **Free access to fee-only online medical journals**
- **More information about drug interactions**
- **Online diagnostic tools or "symptom finders"**
- **Access to my medical records and test results**
- **Better ways to connect with local resources**

By the early years of the 21st century, all practicing US clinicians were seeing patients and caregivers who had searched the Internet for medical information. Even though many doctors still overlook or deny its importance, the Internet has already become an important factor in patient care: One e-patient in seven has made an appointment to see a doctor because of information or advice she found online. One in seven has asked the doctor to prescribe a specific medication he learned about online.[31] Patients who have searched for medical information online are considerably more likely to consult doctors than are non-e-patients.[32] And since their ability to use the Internet makes them feel less dependent, less fearful of the unknown, more capable of asking well-informed questions, and more capable of evaluating the care their medical professionals provide,[33] e-patients come to the clinic much better prepared than those who have not used online health resources. As one e-patient explained:[34]

> **Our first visit to the neurologist, when my son was diagnosed with autism, was not as devastating as it could have been. My husband and I were well informed and had already figured out the diagnosis by the time we saw the doctor. By being better informed, that first visit was very informative and constructive because we knew the background information, weren't in denial, and could discuss therapies and tests in a logical way...**

How Clinicians Have Responded

Back in 1994, Dr. Blakely obviously felt no responsibility for providing Mr. Murphy with the medical information he wanted. This may strike the contemporary reader as callous and insensitive. But it was not at all unusual at the time. For it was only that year that the Medical Library Association's Consumer and Patient Health Information Section first passed a resolution

calling on hospital libraries to admit interested patients. As it happens, Ms. Lindner, the medical librarian who uncovered Mr. Murphy's impersonation of his doctor, was the chair of that section of the MLA. She recounted Mr. Murphy's story when she introduced me as that year's keynote speaker.

Painful as it may be for older readers to remember, and difficult as it may be for younger readers to imagine, Dr. Blakely's attitude was the rule, not the exception: "For most of the 20th century, patients were routinely and systematically denied access to the best available in-depth medical information about their conditions and the medical treatments currently available," says White Paper advisor Alan Greene, a clinical professor of pediatrics at Stanford and an attending pediatrician at Stanford Children's Hospital. "I was trained never to share the deeper levels of my thinking with patients. And when I took the Hippocratic Oath in 1989, I swore to keep my medical knowledge secret. So until quite recently, becoming a knowledgeable, involved, and medically competent participant in one's own medical care was simply not permitted. And there is still a good deal of physician resistance, resentment, and denial of the inescapable fact that the Internet is helping patients become better informed, more responsible, and more medically competent."[35]

The range of clinician response to e-patients' online research efforts was nicely summarized by one participant in a Pew Internet Project e-patient survey who wrote that he had encountered two distinctly different attitudes among his clinicians: One group encouraged his online efforts, supported his desire to become more medically competent, and clearly thought of themselves as "partners in my care." A second group of clinicians was clearly uncomfortable with, and sometimes seemed to feel offended by, his attempts to learn more about his condition via online resources. They clearly preferred, as he put it, the traditional "him chief, me Indian" model of the doctor-patient relationship.[36]

Many other e-patients have also observed these two conflicting attitudes among their clinicians. Here are some verbatim e-patient perspectives from a Pew Internet Project e-patient survey:[37]

Internet Friendly Clinicians

- **My own doctor is extremely supportive of me and he is happy to discuss any Web-based information I take to him. At times he has been pleased to use my contributions to update his own knowledge base.**
- **My doctor is delighted, in fact likes receiving printouts of information from the Web. He encourages such self-help and research completely.**
- **Continuously discussing with our oncologist, both treatment plans, and alternative procedures found or discussed on several cancer sites, as well as data mining of the Web for such things as clinical trials, and information regarding the course of treatments and their side effects.**

> **• Our doctors are VERY supportive of how proactive we are about our health and like the fact that we use the net to gain additional information and use that information to clarify our questions for her. Our doctor has suggested various websites to us as well.**
>
> **• I was referred to [my current support group] by my doctor… I have also started discussing [our support group discussions] with [my other] doctor who is happy that I have found a website to help me answer any questions I have in detail… my doctor [and I discuss] what I have found… [and] talk about the treatment plans that I then feel [most] comfortable with…**

Internet Hostile Clinicians

> **• If I mention… that I read something about [my condition] on the internet, the doctor immediately gets an attitude and tells me not to believe anything I've read on the internet. It doesn't seem to matter that I've gotten this information from (well known) medical journals.**
>
> **• I took an article I had printed out [from the Internet] to my family practice doctor… and had her react with great anger and throw the paper in the trash.**
>
> **• One [of my doctors] commented that online information is like scribble on a bathroom wall… [I guess he meant] trash or untrustworthy. I have found the opposite.**
>
> **• [The] neurologist at my hospital went wild with fury that I would DARE look up my problems on line and try to participate in my own health care…**
>
> **• My children's GI [doctor] refused to even look at the information I brought in… Most roll their eyes…They [just] assume you have bad information... [without] even seeing your sources.**
>
> **• [Most doctors] really do like to think they have ALL the answers. This will never change, I am afraid. Countless days, weeks, months of suffering are happening to so many patients because doctors refuse to learn new things from the Internet.**

Internet-hostile clinicians sometimes try to discourage their patients from going online. But when they do, one of two things happens. Either the patient leaves the practice and finds a new physician, or the patient goes “underground,” continuing Internet research but not sharing it with the Internet-hostile physician.[38]

Regardless of their clinicians’ response, nearly all e-patients feel that Internet health resources are very helpful indeed. Most e-patients report that their use of the Internet makes them feel more autonomous and capable, not only in their interactions in dealing with the health challenges they face, but also in their dealings with their clinicians and provider institutions. As another e-patient from the same survey explained:

> **Knowledge is power. [What I find online] helps me to feel prepared to talk with doctors and nurses. I know the terminology and the options.**

e-Patients who have “studied up” on their diseases online say they have less fear of the unknown and more confidence in their ability to deal with whatever the future may bring—because of what they have learned during their online health research.[39]

From "Patients" to "e-Patients"

The e-patients we've observed have proven themselves so different from earlier generations of patients, so capable of becoming medically competent, and so ready, willing and able to help others—both patients and clinicians—that we've sometimes been forced to ask ourselves whether we can, in good conscience, continue to use the term "patient" in describing them. In contrast to the unfortunate Edwin Murphy, e-patients like Marian Sandmaier are by no means "patients" in the old 20th century sense. When we invited her to summarize what she'd learned about being an e-patient for this chapter she offered the following:

> **For many years, I'd considered our doctors the ultimate authorities on medical matters. By contrast, I had long viewed the Web as suspect territory, where a seeker of health information could easily get lost in the tangled thickets of fallacy, zealotry, and self-promotion. But now I think differently about such things.**
>
> **Even in the midst of my first Web session, long before I knew I had any chance of helping my daughter, I felt a surge of hope. The Internet was far from infallible; some of the sites I visited were confusing, while others contradicted each other on the details. But the Web, at least, was giving me my first vital insights into what Darrah might be up against. And at that point, our doctors, for all their good intentions and years of training and experience, had given us nothing at all.**
>
> **I truly believe that our doctors wanted to do the right thing. But, like most physicians, they are at their best with the common disorders they see every day. And in today's world of growing healthcare complexity and nanosecond doctors' visits, they barely have time to care for the usual and ordinary disorders.**
>
> **This means that we—patients, parents, spouses, and family caregivers—must be prepared to understand and manage our own medical care as never before. Becoming health-savvy is no longer just a smart idea. Managing your family's healthcare has become a medical necessity. And while the Internet must be approached with appropriate caution, it can be a vital, life-changing tool in our efforts to make sure that our families get the best possible care.**

In the chapters that follow we suggest that like Marian Sandmaier, the millions of online medical consumers who make up our first generation of e-patients have already begun to operate in terms of a new healthcare paradigm, a fundamentally different way of thinking about healthcare that turns many of the previous assumptions of the older, 20th century medical model upside down.

We believe that the new healthcare trends, patterns and models that are emerging from their experience will prove extremely important to all of us who are concerned with effective healthcare reform. And we suspect that their new perspectives, experiences, and insights will offer us many useful guidelines for lasting and effective healthcare improvement—if we can only learn to listen. ■

Personal communication, Kathy Lindner, former director (retired), Englewood Hospital Library, Englewood, NJ, March 14, 2002. Names and identifying characteristics have been changed to protect the privacy of those involved.

[2] Personal communication, Marian Sandmaier, May 17, 2004.

[3] Marian Sandmaier, "Listening for Zebras," *The Washington Post*, Jun. 3, 2004.

[4] American Society of Journalists and Authors, "ASJA Presents 2004 Writing Awards," Apr. 28, 2004, < http://www.asja.org/awards/awar2004.php> (Accessed on Jul. 17, 2006)

[5] Pew Internet & American Life Project surveys, February-April 2006 and March 2000. For more information, see "Demographics of Internet Users" (April 26, 2006) available at http://www.pewinternet.org/trends/User_Demo_4.26.06.htm and "Tracking Online Life" (May 10, 2000) available at http://www.pewinternet.org/report_display.asp?r=11 (Accessed on July 22, 2006.)

[6] "New American Academy of Dermatology Survey Finds Patients Do Their Homework," American Academy of Dermatology Survey, October 23, 2002, http://www.aad.org/public/News/NewsReleases/Press+Release+Archives/Other/Homework.htm (Accessed on July 22, 2006.)

[7] "Generation Rx: How Young People Use the Internet for Health Information," Kaiser Family Foundation (December 2001). Available at: http://www.kff.org/entmedia/20011211a-index.cfm (Accessed on July 22, 2006.)

[8] There have been five principal Pew Internet & American Life Project surveys of e-patients to date:

(1) Susannah Fox and Lee Rainie, "The Online Health Care Revolution: How the Web helps Americans take better care of themselves," Pew Internet & American Life Project *(*Nov. 26, 2000). Available at: http://www.pewinternet.org/PPF/r/26/report_display.asp (Accessed on July 22, 2006.)

(2) Susannah Fox and Lee Rainie, "Vital Decisions: How Internet users decide what information to trust when they or their loved ones are sick," Pew Internet & American Life Project (May 22, 2002). Available at: http://www.pewinternet.org/PPF/r/59/report_display.asp (Accessed on July 22, 2006.)

(3) Susannah Fox and Deborah Fallows, "Internet Health Resources: Health searches and email have become more commonplace, but there is room for improvement in searches and overall Internet access," Pew Internet & American Life Project *(*Jul. 16, 2003). Available at: http://www.pewinternet.org/PPF/r/95/report_display.asp (Accessed on July 22, 2006.)

<http://www.pewinternet.org/PPF/r/95/report_display.asp> (Aug. 6, 2004)

(4) Susannah Fox, "Internet Health is Everywhere: A Portrait of America's E-Patients and Caregivers," Presentation to the Quint Conference in Philadelphia (Oct. 27, 2003).

(5) Susannah Fox, "Health Information Online," Pew Internet & American Life Project (May 17, 2005). Available at: http://www.pewinternet.org/PPF/r/156/report_display.asp. (Accessed on July 22, 2006.)

[9] Pew 1, 2, 3, 4

[0] *Harris Interactive Health Care News*, available at: http://www.harrisinteractive.com/news/newsletters_healthcare.asp

[11] *Journal of Medical Internet Research* (available at: http://www.jmir.org/), the *Journal of the American Medical Informatics Association* (available at: http://www.jamia.org/), the *International Journal of Medical*

Informatics (available at: http://intl.elsevierhealth.com//journals/ijmi/Default.cfm), and the British journal, *Health Expectations* (available at: http://www.blackwellpublishing.com/journal.asp?ref=1369-6513&site=1).

[12] For other resources on e-patients, please see *The Ferguson Report.* Available at: http://www.fergusonreport.com/ and http://e-patients.net/. (Accessed on July 22, 2006.)

[13] Pew 5

[14] Humphrey Taylor, “Cyberchondriacs Continue to Grow in America: 110 million people sometimes look for health information online, up from 97 million a year ago,” *Harris Interactive Health Care News*, 2 (May 2002).

[15] John B. Horrigan and Lee Rainie, “Getting Serious Online,” Pew Internet & American Life Project, March 3, 2002. Available at: http://www.pewinternet.org/PPF/r/55/report_display.asp (Accessed on July 22, 2006.)

[16] Susannah Fox, “Today’s E-Patients: Hunters and Gatherers of Health Information Online,” Information Therapy Conference, Park City, Utah, (September 27, 2004). Available at: http://www.informationtherapy.org/rs_conf_matls.html. (Accessed on July 22, 2006.)

[17] Mary M. Cain, Jane Sarasohn-Kahn, and Jennifer C. Wayne, “Health e-People: The Online Consumer Experience,” Institute for the Future, written for the California HealthCare Foundation (August 2000). Available at: http://www.chcf.org/topics/view.cfm?itemID=12540 (accessed on July 22, 2006.)

In their insightful 2000 report, “Health e-People: The Online Consumer Experience,” Cain, Sarasohn-Kahn, and Wayne identified three types of e-patients—The Well, The Newly-Diagnosed, and the Chronically Ill and their Caregivers. —and estimated the relative percentage of each:

The Well (60% of e-patients)

The Newly Diagnosed within Past 12 months (5% of e-patients)

The Chronically Ill and Their Caregivers (35% of e-patients)

[18] Pew 2

[19] Pew 3

[20] Pew 2

[21] Patricia A. Reeves, “How Individuals Coping with HIV/AIDS Use the Internet,” *Health Education Research*, 16 (2001): 709-19. Quote is from page 712. Available at: http://her.oupjournals.org/cgi/content/abstract/16/6/709 [Abstract] (Accessed on July 22, 2006.)

[22] Adapted from Pew 3, with the added suggestions of many advisors and reviewers.

[23] Daniel Hoch and Wambui Wariungi, John Lester, Stephanie Prady and Ellie Vogel, “e-Patient Survey: What Braintalk Members Do Online,” October 1, 2001. Results available at: http://fisher.mgh.harvard.edu/cscw/demo_data.html (Accessed on July 22, 2006.)

[24] Carina von Knoop, Deborah Lovich, Martin B. Silverstein, and Michael Tutty, “Vital Signs: e-Health in the United States,” Boston: Boston Consulting Group, 2003. http://www.bcg.com/publications/files/Vital_Signs_Rpt_Jan03.pdf (Accessed on March 12, 2007)

[25] C. Tuffrey and F. Finlay, “Use of the Internet by Parents of Pediatric Outpatients,” *Archives of Disease in Childhood*, 87 (2002): 534-6. Available at: http://adc.bmjjournals.com/ (Accessed on July 22, 2006).

[26] James M. Metz, Pamela Devine, et al., “A Multi-institutional Study of Internet Utilization by Radiation Oncology Patients,” *International Journal of Radiation Oncology Biology and Physics*, 56 (2003): 1201-5.

[27] Catherine Ikemba, et al., “Internet Use in Families with Children Requiring Cardiac Surgery for Congenital Heart Disease,” *Pediatrics*, 109 (2002): 419-22.

[28] Sue Ziebland, **Alison Chapple, et al.**, "How the internet affects patients' experience of cancer: a qualitative study," *British Medical Journal*, 328 (2004): 564-9. Available at: http://bmj.bmjjournals.com/cgi/content/full/328/7439/564 (Accessed on July 22, 2006).

[29] Pew 2

[30] Pew 3. p. 30, and Christopher N. Sciamanna, Melissa A. Clark, Thomas K. Houston, and Joseph A. Diaz, "Unmet Needs of Primary Care Patients in Using the Internet for Health-Related Activities," *Journal of Medical Internet Research*, 4 (2002). Available at: http://jmir.org/2002/3/e19/index.htm (Accessed on July 22, 2006.)

[31] Humphrey Taylor and Robert Leitman, "Most Cyberchrondriacs Believe Online Information is Trustworthy, Easy to Find and Understand," *Harris Interactive Health Care News*, 2, (June 11, 2002).

[32] Pew 3, p. 15

[33] Pew 3, p. 16

[34] Pew 3

[35] Alan Greene, Personal Communication, Nov. 7, 2003, and Nov. 29, 2003

[36] Pew 3

[37] Pew e-Patients Survey

[38] Fox, "Today's E-Patients."

[39] Pew 3

The difficulty lies, not in the new ideas,
but in escaping from the old ones...
—John Maynard Keynes

CHAPTER TWO
Content, Connectivity, and Communityware

One of the most interesting questions our e-Patient Scholars Working Group came up with in our several years of deliberations was this: "What is it that our professional colleagues don't understand about the importance of e-patients?" Part of the answer comes from the work of John Seely Brown.[1] He notes that when established experts first consider the effects of new information technologies and the cultural transformations they produce, they will typically attempt to do so from within the cultural constraints of their established professional paradigm: their customary point of view from the professional perspective. We were no exception to this rule.

When we first began our explorations of the emerging world of the e-patient, we were all operating from a deeply held set of assumptions about healthcare, many of which were unconscious. But our findings were so unexpected that, time after time, we were forced to consider alternative points of view, including some that we had initially considered unacceptable. This in-depth process of reassessing the medical model that all our professional members had trained in, and had long taken for granted, was among the most difficult, confusing, and contentious part of our deliberations. But as Brown discovered, and as we came to agree: [2]

> **Really substantive innovations—the telephone, the copier, the automobile, the personal computer, or the Internet—are quite disruptive, drastically altering social practices. [So] our established experts may be those *least* capable of helping us find our way safely through the disruptive social innovations these new technologies will require.**

We finally came to this consensus conclusion with respect to e-patients: The e-patient revolution cannot be adequately understood in terms of our older medical constructs, including many still taught in medical school and reaffirmed in continuing medical education. Those who attempt to view the experience of today's e-patients through the cultural lenses of yesterday's professionally centered clinicians are doomed to failure. They will be blinkered if not blinded by what Brown calls their professionally centered tunnel vision.

Our most helpful insights came from a growing awareness that e-patients use the Internet in three fundamentally different ways: to access content, connect with others, and collaborate with others in ways never possible before.

We came to realize that instead of noticing and investigating the vital social cues that might lead to a deeper and more subtle understanding, many clinicians, many of the medical researchers who have attempted to study or explain e-patients, and many of the leading figures in medical quality reform, have overlooked, ignored, downplayed or even actively opposed some of the most innovative and creative developments in modern medicine.

The extraordinary added value brought to health care through networks, online or otherwise, was not initially evident to the traditionally trained members of our group. We started out as prisoners of our deeply ingrained professionally centered paradigm. *If* we emerged from this paradigm, even partially, we have our e-patient fellow members to thank. We are most grateful to them.

The world that has opened to us as the result of information technology is, as Alan Kay suggests, “an entirely new medium—the next 500-year idea,” rendering many of our older practices obsolete. “You'll have to find a way to lock into what’s best for the patient,” Kay told an audience of 3,000 medical informatics professionals at the September 2004 MedInfo conference in San Francisco, “not what’s most comfortable for you.”[3]

In the time-honored spirit of disruptive science, we propose the following principles of patient-driven healthcare as a challenge to our old paradigm. We modestly suggest that the tentative conclusions below are no more “anti-doctor” or “anti-medicine” than the conclusions of Copernicus and Galileo were “anti-astronomer.”

Patient-Driven Healthcare: Seven Preliminary Conclusions

---

1. e-Patients have become valuable healthcare resources and providers should recognize them as such.

The e-patient revolution has already changed the experiences of millions of patients and family caregivers across the globe. But it has had little impact on the workflow at most clinical facilities. Many clinicians have become somewhat more Internet-friendly, though some still stiffen when a patient presents a printout from a health site or journal article. At most clinical facilities, one would struggle to find a single formal change reflecting e-patients’ new abilities.

Few clinicians ask patients and caregivers what they already know about their illness, what other sources they have consulted, and which have been most valuable. Few clinicians ask

their patients, "What do *you* think we should do?" Few clinicians introduce patients with the same disorder to one another. And few clinicians advise their newly diagnosed patients with diabetes, Bell's palsy, or breast cancer that they might find useful support and guidance in a face-to-face or online support community.

Many provider organizations still do not ask patients for their e-mail addresses. Few ask patients to provide in-depth in-their-own-word critiques of the services they receive. Fewer still invite patients to join their clinical work teams, their advisory boards, or their governing councils—even though many would be well qualified.

Thus for our first generation of e-patients, going to the doctor's office is all too frequently a Kafkaesque experience of going back in time to when the Internet did not exist. We must learn to invite our capable fellow-citizens to join us in our common healthcare efforts. And such collaborations are especially important for patients with a chronic disease.

As Bodenheimer and his colleagues have noted, patients with chronic conditions make so many of the key day-to-day decisions about their illnesses that they are already effectively managing most of their own medical care.[4] When clinicians acknowledge and support their patients' essential role in self-management, providing them with the skills, tools, and support they need to manage their care even more effectively and supporting their efforts to provide helpful care for themselves and others, they exhibit fewer symptoms, demonstrate better outcomes, and require less professional care.

2. The art of "empowering" patients is trickier than we had thought.

Some 20th century clinicians and health educators once believed that it might be possible to "empower" patients by providing them with targeted medical information, "prescribing" selected medical content in much the same way that clinicians currently order prescription drugs. We now know that empowering patients requires a change in the patient's level of engagement, both with their illness and with their clinicians, and in the absence of such changes, clinician-provided content has few, if any, positive effects.

Some studies have even suggested that such top-down "targeting" of patients by professionals may be harmful. When Elizabeth Murray and her colleagues at the Cochrane Collaboration reviewed the medical literature for studies in which chronic disease patients had been targeted by professionally-developed interventions via the Internet, they found that even though the targeted patients showed an increased knowledge of their condition and an increased

perception of social support, the interventions had no effect on the patients' sense of self-efficacy or on their health-related behaviors.[5] And these patients had worse outcomes than those who were left alone.

The same effect has been observed in other professional efforts supposedly intended to "empower" patients, (e.g., telemedicine, Information Therapy, patient handouts, etc.). Unless the underlying relationships between clinicians and patients are fundamentally transformed, professional interventions do not empower patients.[6]

For the earliest evidence of this insight, consider the work of White Paper advisor Kate Lorig and her colleagues at Stanford's Patient Education Research Center (http://patienteducation.stanford.edu). Beginning in the early 1980s, as part of their efforts to help patients learn to manage their health more effectively, Lorig and her colleagues discovered that when it came to empowering arthritis patients, "Content did not matter." The success of Lorig's early programs came not from the specific medical content they provided (e.g., "rheumatoid arthritis is a disease of the connective tissues in which inflammation affects the joints and other organs of the body"), but from successfully communicating the idea that patients really can effectively manage their own illnesses. This concept was imparted through "patients-really-can-run-the-show" messages, encouragement, support, training and rehearsing, as well as proactive modeling in the art of devising and carrying out an action plan based on one's own agenda, be it losing weight or confronting an inadequate physician.[7]

Just as Lorig's model would predict, many professionally developed IT systems designed to "empower" patients have just the opposite effect. In one classic analysis of such a system, the late Diana Forsythe, a medical anthropologist at the University of California-San Francisco School of Medicine, described what happened when a distinguished team of medical informatics professionals set out to build a computerized patient-education system for migraine patients. Her paper, "New Bottles, Old Wine: Hidden Cultural Assumptions in a Computerized Explanation System for Migraine Sufferers,"[8] should be inscribed on the cubicle walls of all those who aspire to develop online systems for patients.

The senior developers in Forsythe's study made four assumptions: (1) that the patients knew nothing about migraines, (2) that what they needed to know was what their doctors wished to tell them, (3) that the only reason the clinicians hadn't provided the information patients needed was a lack of time, and (4) that while the patients had much to learn from their

neurologists, the clinicians had nothing at all to learn from their patients. Forsythe concluded that all of these hypotheses were mistaken.

Many of the migraine patients had read extensively in the medical literature and most knew a great deal about their condition, including many things that their physicians did not know, e.g., exactly what happened during a migraine attack and the best practical ways to manage it. The things the patients wanted to know were radically different from the things the neurologists wanted to tell them.

Other patients were perfectly capable of supplying much of the information their fellow-patients needed. A long list of "nonmedical" questions concerned patients greatly but was not taken seriously by the physicians. The neurologists systematically ignored these questions.

When patients attempted to bring up concerns that their neurologists did not see as strictly medical, they often appeared not to 'hear' them, or "attempted to pass the matter off as a joke." Thus the clinicians effectively ignored or disregarded all but a few "approved" types of questions. The neurologists' failure to answer their patients' questions had little to do with time. They offered little explanatory material and dominated the patient interviews so completely that patients had few chances to ask questions.

The system's final version did not allow patients to raise the questions that the clinicians habitually ignored. It did not permit patients to describe their experiences in their own words, but forced them to choose from a limited list of doctor-defined options. It did not allow patients to present the many things that they knew that the clinicians did not. It did not permit patients to inform other patients on topics that they knew more about than the doctors. It did not permit patients to provide the doctors with useful feedback on their services. It also failed to provide a way for patients to give their doctors important feedback and help keep them abreast of the literature.

Like many clinicians today, the developers were unwilling to address the important issues of knowledge, power, and control among doctors, non-physician health professionals, and patients that are vital to building any effective IT system. So in the end, they built a technically impressive system that reflected all the same profound cultural defects of care the patients had experienced repeatedly in the clinic.

Forsythe's untimely death—she died while mountaineering in Alaska—deprived us of one of our most insightful observers in the emerging field of e-patient studies. But we can honor her memory by remembering what she taught us. "In practice," she once told me, late at night after

a long AMIA (American Medical Informatics Association) meeting, “most medicine is ten parts culture to one part real science. From a medical anthropologist’s point of view, a good deal of what happens in healthcare is invisible to most clinicians. Imagine that.”[9]

3. We have underestimated patients’ ability to provide useful online resources.

Several years ago, a visitor to my website posted the following question:[10]

> **Medical websites created by persons who are not physicians are unreliable and should, *de facto*, be considered medically unsound. Don't you agree?**

After some consideration, I discussed the matter with White Paper advisor John Grohol, psychologist, author, and webmaster of the highly respected comprehensive mental health site for patients and professionals Psych Central (http://www.psychcentral.com). Grohol is also the author of the definitive book on the field. As it happened, John had recently selected a short list of the “best of the best” online resources in mental health from his extensive directory of mental health websites. His rating scale took into account four areas in each website: content, presentation, ease of use, and overall “experience.” The quality of the content was weighted more heavily than either presentation or ease of use. We decided to address the question above by determining whether the developers of Grohol’s 16 “Top Sites” had been professionals or patients. Our conclusions: ten (62.5 percent) of the 16 top sites were produced by patients, five (31.25 percent) by professionals, and one (6.25 percent) by a group of artists and researchers at Xerox PARC.[11]

We observed that the patients who produce these sites certainly don't know everything a physician might know, but they don't need to. Good clinicians must have an in-depth working knowledge of the ills they see frequently and must know at least a little about hundreds of conditions they rarely or never see. Online self-helpers, on the other hand, will typically know only about their own disease, but some will have an impressive and up-to-date knowledge of the best sources, centers, treatments, research, and specialists for this condition. Smart, motivated, and experienced self-helpers with hemophilia, narcolepsy, hemochromatosis, or any number of rare genetic conditions may well know more about current research and treatments for their disease than their own primary care practitioner. And when it comes to aspects of illness that some clinicians may consider secondary—e.g., practical coping tips and the psychological and social aspects of living with the condition—some experienced self-helpers can provide other patients with particularly helpful advice. The things clinicians know

and the things self-helpers know can complement each other in some interesting and useful ways.

We concluded by quoting online patient-helper Samantha Scolamiero, founder of the BRAINTMR mailing list:[12] "Group members—lay folk and professionals alike—have moved beyond the old, obsolete mindset that holds that only certain 'qualified' medical professionals may create and disseminate medical information. We lay folk are learning that we are qualified through our experience, our knowledge, and our concern. We now see that we are capable of contributions no professional can make and that by linking our efforts [with those of professionals] in a coordinated team, we can advance the well being of all."

To better understand the power of online help provided by patients, consider the case of Karen Parles. Parles was a research librarian at a major New York art museum until January 1998, when she learned she had advanced lung cancer. "My doctors told me my cancer was incurable, that even with chemotherapy I had only a year or so to live," she recalls. "I'd never smoked, I have two great kids, and I was only 38. So the whole thing came as quite a shock. I was pretty overwhelmed at first. But as soon as I could, I went onto the Internet, looking for information.[13]

"I'm pretty good at finding things online. But even so, I had a hard time locating all the information and people I needed. There was great stuff out there, but it was scattered across dozens of different sites. There was no comprehensive site that provided links to all the best online information for this disease.

"I found a great support group, the ACOR LUNG-ONC mailing list.[14] The patients on the list answered my questions, suggested useful sites, and gave me a lot of invaluable support. I can't overemphasize the importance of their help in those early weeks and months. It gave me instant access to the wisdom and experience of hundreds of other lung cancer patients."

Then a friend told Karen about a surgical team developing a new treatment for her type of cancer at Boston's Massachusetts General Hospital. "I went to Boston to see them and I was pretty impressed," she says. "But having a lung removed by an unproven procedure still seemed frightening, so I shared my fears with my LUNG-ONC friends. I heard right back from eight or ten others who'd had a pneumonectomy. They assured me that I could do it and encouraged me to give it a shot."

"After I recovered from the surgery, I got to thinking," Karen recalls. "I'd found my life-saving treatment by a combination of Internet-smarts, luck, and personal contacts. Others might not be so lucky. So I decided to create an online resource just for lung cancer, a single, centralized site where patients could find links to everything they needed to help them get the best possible medical care, a place where they could learn to manage their disease in the best possible way."

Parles launched her site Lung Cancer Online (http://www.lungcanceronline.org) in 1999. It lists and describes the best sites containing information about lung cancer. She encourages newly diagnosed users to craft their own crash course in their disease. Visitors find guidelines and databases to help them find the top-rated lung cancer specialists and the best medical centers for each type of lung cancer. There is also practical advice with survivors' stories, up-to-date information on the latest clinical trials, and guides to mining the medical literature. The site also refers visitors to a variety of online support groups.

In addition, visitors have had access to Karen herself. "Because I'm an experienced lung cancer survivor, I've found that many patients and family members want to interact with me personally, by phone or e-mail or in person," she explains. "So in addition to keeping my site up-to-date, I spend many hours each week helping other patients, sharing my story, and providing hope and encouragement." Many of the patients who have come to rely on her counsel and website refer to Karen as their mentor, their role model, or their hero.

With characteristic modesty, Parles urged us to emphasize the important roles her support group, her extensive network of other lung cancer patients, and her network of expert medical advisors all play in maintaining and updating her site. "I work collaboratively with the webmasters and members of all the sites and groups I link to. I depend on others for the questions I receive and the resources I hear about. So lungcanceronline.org serves as a conduit for many voices."

4. We have overestimated the hazards of imperfect online health information.

In the early days of the World Wide Web, we all worried about what would happen to patients who found incorrect, outdated, or self-serving medical advice online. We now know that they rarely come to harm. Two comprehensive efforts have researched whether e-patients are being injured as a result of bad online information. In the first, a distinguished international team that included White Paper advisor Alejandro Jadad of the University of Toronto conducted an extensive review of the worldwide medical literature, looking for reported cases

of patients who had died because of bad online information or advice. They found that for the first decade of the Internet's existence, only a single case had been reported and the evidence was by no means conclusive.[15] In the second, White Paper advisor Gunther Eysenbach and his colleagues established the Database of Adverse Events Related to Internet Use (DAERI), appealing to clinicians worldwide to report case studies of harm to patients resulting from bad online information and offering a fifty-euro "bounty" for each report. As of September 2004, Dr. Eysenbach reported that in the first four years of the project, only a single case of a possible fatality was reported.[16] The project has since been discontinued.

By contrast, in the "offline" world of medical care, a survey by the National Patient Safety Foundation found that 42 percent of U.S. adults reported that they had experienced a medical error—either personally or to a relative or friend.[17] The Institute of Medicine has estimated the number of hospital deaths due to medical errors at 44,000 to 98,000 annually.[18] A 2004 HealthGrades, Inc., study puts this number at 195,000.[19,20] A collaborative national surveillance project conducted by the Centers for Disease Control and Prevention, the Food and Drug Administration and the U.S. Consumer Product Safety Commission reported in 2006 that adverse reactions to common medicines sent at least 700,000 Americans to emergency rooms annually.[21] *The experts* who compiled and analyzed the data concluded that this was probably a conservative number because so many severe drug reactions go undiagnosed.

Professor Barbara Starfield of Johns Hopkins University concluded that 225,000 U.S. deaths annually can be attributed to iatrogenic causes—medical mistakes, adverse reactions, hospital-based infections, etc.[22] This number would make healthcare hazards our third leading cause of death, surpassed only by heart disease and cancer. The HealthGrades, Inc., researchers concluded that: "Medical errors seem to be the elephant in the room that no one wants to acknowledge or talk about. The lack of recognition and acknowledgment of the seriousness and urgency of the problem fosters a culture of denial and complacency."[23,24]

We can only conclude, tentatively, that adopting the traditional passive patient role and putting oneself in the hands of a medical professional may be considerably more dangerous than attempting to learn about one's medical condition on the Internet. We suspect that the over-generalized warnings some clinicians have given their patients about the "bad quality of health information on the Internet" will in retrospect be considered counterproductive and ill advised.

Why did many clinicians assume that the Internet would be so much more dangerous for their patients than it actually turned out to be? We don't believe that this can be entirely explained by self-serving motives. Clinicians are most familiar with closed information systems—such as electronic medical records and the systems that report laboratory values. Closed information systems rely on supply-side quality control measures: Only the duly authorized can add or edit information; the accuracy and currency of data is closely monitored; and there is little tolerance for error. Thus the data in closed systems is assumed to be universally accurate.

By contrast, the Internet is an open information system, albeit one that provides access to many closed information systems. Anyone can publish anything they wish. The Internet, therefore, will always contain a good deal of inaccurate, outdated, and self-serving information. But while this would be a major defect in a closed information system, in an open information system like the Internet, it is merely a system characteristic.

Open systems rely on demand-side quality control measures. Thus sophisticated Internet users understand that one does not accept any random bit of online information (e.g., spam) with the same degree of unquestioning acceptance that might be appropriate when checking one's bank balance or looking up a patient lab value. Unlike some patients at their doctor's offices, patients who use the Internet don't leave their critical faculties at the door. And even though the Internet contains imperfect information, it also offers its users many valuable tools that can help them tell the good from the bad.

This added value of connectivity and communities is enormous. As David Reed has put it, "What we see, then, is that there are really at least three categories of value that networks can provide: the linear value of services that are aimed at individual users, the 'square' value from facilitating transactions, and exponential value from facilitating group affiliations. What's important is that the dominant value in a typical network tends to shift from one category to another as the scale of the network increases."[25]

Pattie Maes, one of our most highly regarded IT visionaries, has defined a whole new category of online quality-control systems she calls *communityware*.[26] In addition to helping users find the high-quality online information they seek, communityware systems help loose online networks of people with common interests accomplish tasks that formerly required centralized control and coordination: choosing the best doctor or medical center, for example, deciding on the most appropriate treatment for their disease, or providing advice and referrals for other patients. Communityware systems, Maes notes, are driving a major power shift from

established authorities to online communities, not just in medicine, but in every sector of society.[27]

The software that enables online support communities—listservs™, Web forums, chat rooms, etc.—provide one example of communityware. The proprietary PageRank algorithm used by Google to identify Karen Parles' site as the number one resource for lung cancer is another.[28,29] Online databases that provide user reviews and ratings (for examples, see the seller reputation databases at ebay.com, the product reviews at www.amazon.com, and the patient drug reviews at www.askapatient.com) are another form of communityware. Different forms of electronic communication can also serve as platforms for communityware; think of e-mail networks, blogs, instant messaging, text messaging, moblogging, wikis, P2P file sharing networks, and other collaborative technologies.

5. Whenever possible, healthcare should take place on the patient's "turf."

White Paper advisors John Lester, Dan Hoch, and their colleagues did not begin their groundbreaking e-patient project Braintalk by requiring patients to come to their clinical offices at Massachusetts General Hospital. They went to where the e-patients were and observed what they were doing. And once they had learned what online support communities were and how they operated, they offered to help them do what they were already doing.

Similarly, White Paper advisors Alan and Cheryl Greene, developers of drgreene.com, did not launch their groundbreaking pediatric site by designing a technically sophisticated "consumer health informatics" system they imagined their patients might like. Instead, they asked their patients what they wanted—and gave them what they asked for. It was because they understood that they could accomplish their purposes more effectively by going to the e-patients' familiar "turf," rather than by forcing patients to come to their familiar professional terrain, that these pioneering developers were able to make such important contributions to the online healthcare commons.

In their book *The Support Economy*,[30] Shoshana Zuboff and James Maxmin draw an important distinction between the traditional realm of professional/organization space (e.g., the clinic and the hospital) and the rapidly evolving realm of individual/consumer space (e.g., the e-mail inbox, the search engine, the website, and the virtual community) in the emerging information age economy.

Professional/organization space is the traditional turf of clinicians and researchers. It is completely within the medical professional's realm of control. When operating in professional/organization space, patients must conform to their clinicians' "rules, procedures, standards, requirements, and schedules, not the other way around."[31] And while this may feel perfectly natural to most clinicians, Zuboff and Maxmin warn that the experiences, capabilities, and needs of our first generation of e-patients have changed so dramatically that professionals who still insist on operating exclusively within organizational space simply "do not have the capacity to perceive [these] individuals' [new reality], let alone to respond to their [real] needs… even as a perpetual drumbeat of consumer-oriented rhetoric claims the opposite."[32]

Zuboff and Maxmin define individual/consumer space: "Individual space is always the individual's space, and the choices that exist there are always the individual's choices. Each individual sets the rules of relationship… [and] these relationships cannot be constructed mechanistically. They are necessarily intimate and authentic. They build over time, based on mutual respect and interpersonal trust."[33]

Individual/consumer space is another name for what we've been calling *self-help cyberspace*, the domain within which autonomous e-patients find and use the online health resources of their choosing—friends and family, patient-helpers, online communities, medical websites, and unstructured e-mail with clinicians. Individual/consumer space—or self-help cyberspace—always lies within the e-patient's realm of control.

Most of the most successful online health resources developed to date—that is to say, those that e-patients like best (and we would argue that this is the only appropriate standard)—operate in individual/consumer space. e-Patients, not professionals, are in charge. Most have been developed by patients. And those developed by professionals have almost always relied heavily on patient direction and guidance.

Systems that operate in individual/consumer space liberate patients from the confining, limited, and subservient roles they are typically forced to play when interacting with clinicians within organizational/professional space. For even though such limits are often invisible to health professionals, most patients feel intensely uncomfortable when forced to operate within the constricted roles allowed them in most contemporary healthcare institutions.

Zuboff and Maxmin warn that developers who continue to believe that it may be possible to develop systems that genuinely empower patients while remaining within the comfortable and familiar borders of professional/organization space are only fooling themselves. By way of

explanation, they tell the story of the man who looks for his keys under the streetlight, instead of in the dark alley where he lost them, because that's where the light is. Like the man in the parable, "we professionals like to look for solutions within our accustomed organizational/professional space, even though the real area of concern is not to be found there, but in what seems to us the darkness, but in reality is simply a different world, the world of individual/community space…. [But] consumer solutions developed in organization space merely perpetuate the fundamental problems whose symptoms they seek to address... When we face discontinuity, the answers we seek cannot be found under the light from the lamppost. They are not here, but over there—in the darkness, through the looking glass, on the other side of what [we think we know]." [34]

6. Clinicians can no longer go it alone.

A century ago, a physician's brain could hold all the medical information required to treat most known medical conditions; there was not that much to remember. Over the past century, medical information has increased exponentially. But as White Paper advisor Richard Rockefeller has observed, the capacity of the human brain to store and process it has not.[35] As Donald Lindberg, director of the National Library of Medicine, explains, "If I read and memorized two medical journal articles every night, by the end of a year I'd be 400 years behind."[36]

It is no longer possible for medical professionals to stay completely up-to-date on a long list of medical conditions. It is difficult enough to do so for a single disorder. So unless e-patients are lucky enough to find a clinician who is a top specialist in their disease (and as we will see, a surprising number have done exactly that), they will be able to learn more from the Internet than they can in their doctor's office. And some will end up knowing more than their clinicians about many, if not most, aspects of their conditions. Understanding this development on an intellectual level is one thing. But as many of us can assure you, it is quite another to sit face-to-face with a patient newly diagnosed with a rare condition who has already spent countless hours reading up on it—while you can only dimly remember reading about it in medical school many years before.

We must admit that in the beginning, dealing with patients who knew more than we did was something of a shock. So we can fully understand and sympathize with colleagues who may be tempted to discount their patients' new abilities, to disparage their information sources, and to attempt to reassert their medical authority. We can also assure them that, in the long run,

this will be neither possible nor necessary. For in the end, we're confident that our colleagues will conclude, as we have, that informed, activist e-patients are a blessing, not a curse.

Well-informed patients can help lift the burden of care from the shoulders of overworked clinicians. They can take their own vital signs during a clinical visit. They can do their own lab tests. They can examine their kids' sore ears with a home otoscope, listen to their own hearts to monitor an arrhythmia, administer their own peritoneal dialysis, give themselves injections, and can safely choose and self-administer many drugs currently available only by prescription. And, as we will see in the next chapter, they can help us upgrade our customer services and improve the quality of our clinical care, helping us avoid, detect, and manage medical mistakes. They can, in short, become our colleagues and our partners, not irresponsible children or mindless robots, capable of nothing more than obeying their doctors' orders without question or comprehension. To continue to prohibit patients from making the healthcare contributions they now can make would be an exercise in futility.

7. The most effective way to improve healthcare is to make it more collaborative.

As we have seen, the emerging world of the e-patient cannot be fully understood and appreciated within the context of pre-Internet medical constructs. The 20th century medical worldview did not recognize the legitimacy of lay medical competence and autonomy. That is why its metrics, research methods, and cultural vocabulary are poorly suited to studying this emerging field. Deeply embedded cultural assumptions about the roles of physician and patient will often lead to a premature closure of reality.

The e-patient revolution we have been describing, encouraging and hopeful as it may be, is not a complete solution to our healthcare ills. We cannot simply replace the old physician-centered model with a new patient-centered model. Like software engineers faced with the challenge of creating a new computer operating system, we now find ourselves faced with the task of identifying and upgrading the areas in which our previous "operating system" has proved itself inadequate. We must develop a new collaborative model, akin to open-source software, that draws on the strengths of both the traditional and the emerging healthcare systems. We must find new ways to help 21st-century clinicians and 21st-century patients work together in 21st-century ways. In the chapters that follow, we offer more suggestions on how we might accomplish this. ■

[1] John Seely Brown and Paul Duguid, *The Social Life of Information* (Boston: Harvard Business School Press, 2000).

[2] John Seely Brown, “Innovating Innovation,” in *Open Innovation: The New Imperative for Creating and Profiting from Technology* by Henry William Chesbrough (Boston: Harvard Business School Press, 2003).

[3] Alan Kay, “Keynote Presentation.” Presented at MedInfo 2004, San Francisco, September 8, 2004, Cassette tape available from <https://www.nrstaping.com/amia/amia2004.htm> (Accessed on Oct. 25, 2004).

[4] Thomas Bodenheimer, Kate Lorig, Halsted Holman, Kevin Grumbach, “Patient Self-management of Chronic Disease in Primary Care,” *Journal of the American Medical Association*, 288 (2002): 2469-2475, <http://jama.ama-assn.org/cgi/content/full/288/19/2469> (Accessed on Oct 2, 2006).

[5] Elizabeth Murray, J. Burns, S. See Tai, R. Rai, et al., “Interactive Health Communication Applications: the story of a Cochrane review” [Abstract], *Cochrane Consumers and Communication Review Group* no. 11 (September 2004): 6-7. Newsletter no longer available online. Full text available from elizabeth.murray@pcps.ucl.ac.uk.

[6] P.G. Gibson, J. Caughlan, A.J. Wilson, M.J. Hensley, et al., “Limited (information only) patient education programs for adults with asthma,” *The Cochrane Library* (Chichester, UK: John Wiley & Sons, Ltd., 2004). <http://www.cochrane.org/cochrane/revabstr/AB001005.htm> (Accessed on Oct 2,2006).

[7] H. R. Holman and Kate R. Lorig, “Patient education: Essential to good health care for patients with chronic arthritis,” *Arthritis and Rheumatism* 1997 Aug;40(8):1371-3. Kate Lorig, V. M. Gonzalez, D. D. Laurent, L. Morgan, and B. A. Laris, “Arthritis self-management program variations: Three studies,” Arthritis Care and Research 11(1998): 448-54.

[8] Diana E. Forsythe, “New Bottles, Old Wine: Hidden Cultural Assumptions in a Computerized Explanation System for Migraine Sufferers,” in *Studying Those Who Study Us* by Diana E. Forsythe (Stanford, CA: Stanford University Press, 2001).

[9] Diana Forsythe, Personal Communication, Nov 11, 1994.

[10] Tom Ferguson, “Can Useful and Reliable Online Health Resources be Produced by 'Medically Unqualified' Persons?” *The Ferguson Report*, no. 5, Jul. 1999, <http://www.fergusonreport.com/articles/fr079902.htm> (Oct. 15, 2004).

[11] Tom Ferguson, “Can Useful and Reliable Online Health Resources Be Produced by ‘Medically Unqualified’ Persons?” *The Ferguson Report*, no. 9, Nov. 1999, <http://www.fergusonreport.com/articles/fr079902.htm> (Aug. 5, 2004). For a current listing of Grohol’s top online resources for mental health, see www.psychcentral.com.

[12] Tom Ferguson, *Health Online: How to Find Health Information, Support Groups, and Self-Help Communities in Cyberspace*, (Reading, MA: Addison-Wesley Publishing/Perseus Press, 1996, 170–171).

[13] Karen Parles quotes are drawn from several phone and face-to-face interviews conducted by Tom Ferguson from 1999 to 2002. Two earlier articles based in part on these interviews with Parles have previously appeared: (1) Tom Ferguson, “Patient-Helpers and Physicians Working Together: A New Partnership for High-Quality Health Care,” *The Ferguson Report*, no. 6, Sep./Oct. 1999, <http://www.fergusonreport.com/articles/fr109902.htm> (Accessed on Aug. 5, 2004); (2) Tom Ferguson, “Online Patient-Helpers and Physicians Working Together: A New Partnership for High-Quality Health Care,” *British Medical Journal*, 321 (2000): 1129-32. <http://bmj.bmjjournals.com/cgi/content/full/321/7269/1129> (Accessed on Aug 6, 2004); Additional material from Karen Parles has been adapted from: Richard T. Penson, Renee C. Benson, et al., “Virtual Connections: Internet Health Care,” *The Oncologist*, 7 (2002): 555–68. <http://theoncologist.alphamedpress.org/cgi/content/full/7/6/555> (Accessed on Aug. 6, 2004).

[14] The URL for the Lung-Onc lung cancer mailing list is <http://www.acor.org/lung-onc.html> (Accessed on Aug. 6, 2004).

[15]Anthony George Crocco, Miguel Villasis-Keever, Alejandro R. Jadad, “Much Ado About ‘Nothing’? Looking for Evidence on Harm Resulting From Health Information on the Internet.” Paper presented at the Fourth International Congress on Peer Review in Biomedical Publication, Barcelona, Spain, Sep. 15, 2001, <http://www.ama-ssn.org/public/peer/prc_program2001.htm#ado> (Accessed on Jul. 29, 2004).

Anthony George Crocco, Miguel Villasis-Keever, Alejandro R. Jadad, “Analysis of cases of harm associated with use of health information on the internet,” *Journal of the American Medical Association*, 287 (2002): 2869-71.

Richard Smith, “Almost no evidence exists that the internet harms health,” *British Medical Journal*, 323 (2001): 651.

[16] Gunther Eysenbach, personal communication to author, AMIA Meeting, Sep. 2004.

[17] National Patient Safety Foundation at the AMA: *Public Opinion of Patient Safety Issues*, Louis Harris & Associates, Sep. 1997.

[18] Institute of Medicine (IOM), *To Err Is Human: Building a Safer Health System*, (Washington, DC: National Academies Press, 1999). <http://www.nap.edu/openbook/0309068371/html/> (Accessed on Nov. 1, 2004).

[19] HealthGrades, Inc., “Patient Safety in American Hospitals,” Jul. 2004, <http://www.healthgrades.com/media/english/pdf/HG_Patient_Safety_Study_Final.pdf> (Jul. 15, 2004).

[20] Medscape, “Report Estimates 195,000 Deaths Annually in U.S. Are Due to Hospital Error,” Jul. 28, 2004, <http://www.medscape.com/viewarticle/484387> (Accessed on Aug. 13, 2004).

[21] *Budnitz, Daniel, S., et al.,* “National Surveillance of Emergency Department Visits For Outpatient Adverse Drug Events,” *Journal of the American Medical Association, 296 (2006): 1858-66.*

[22] Barbara Starfield, “Is U.S. Health Really the Best in the World?” *Journal of the American Medical Association*, 284 (2000): 483-5. <http://jama.ama-assn.org/cgi/content/full/284/4/483> (Accessed on Oct. 26, 2004).

[23] HealthGrades, Inc., “Patient Safety in American Hospitals,” Jul. 2004, <http://www.healthgrades.com/media/english/pdf/HG_Patient_Safety_Study_Final.pdf> (Jul. 15, 2004).

[24] Medscape, “Report Estimates 195,000 Deaths Annually in U.S. Are Due to Hospital Error,” Jul. 28, 2004, <http://www.medscape.com/viewarticle/484387> (Accessed on Aug. 13, 2004).

[25] D. P. Reed, “That Sneaky Exponential—Beyond Metcalfe's Law to the Power of Community Building,” *Context*, March 1999. http://www.contextmag.com/archives/199903/digitalstrategyreedslaw.asp, (Accessed on Jan. 24, 2006).

[26]Pattie Maes, “Community Ware.” Slides presented at MIT Media Laboratory, Cambridge, MA, Jan. 29, 2001.

[27] Maes, 2001.

[28] Phil Craven, “Google’s PageRank Explained,” *WebWorkshop.net*, <http://www.webworkshop.net/pagerank.html> (Aug 4, 2004).

[29] Ian Rogers, “The Google Pagerank Algorithm and How It Works,” *IPR Computing Ltd*., May 16, 2002, <http://www.iprcom.com/papers/pagerank/index.html> (Accessed on Aug. 4, 2004).

[30] Shoshana Zuboff and James Maxmin, *The Support Economy: Why Corporations are Failing Individuals and the Next Episode of Capitalism* (New York: Viking Press, 2002).

[31] Zuboff, 2002, 185.

[32] Zuboff, 2002, 10.

[33] Zuboff, 2002, 328–329.

[34] Zuboff, 2002

[35] Richard Rockefeller, "Web-Based Guidance Systems In Health Care." Presented at Intel's Internet Health Day II, New York, Oct. 12, 1999, <http://www.healthcommons.org/vision/web_based_guidance_systems.html> (Accessed on Aug. 1, 2004).

[36] *ibid*.

The Internet is a place, an environment,
made up of people and their myriad interactions.
It is not merely a technology but a new
way of cooperating, sharing, and caring.
—Vinton G. Cerf

CHAPTER THREE

Patient-Centered Networks: Connected Communities of Care

Searching the World Wide Web for medical information represents only one aspect of the e-patient experience. For many e-patients, online interactions with their own personal networks of family members, friends and other contacts may be equally or more important.

To get a sense of your own personal online network, you need look no farther than your own e-mail inbox and outbox. According to a classification system proposed by Haythornthwaite,[1] those with whom you communicate weekly or more often are defined as your closest ties; your weekly to monthly contacts are your significant ties; and those with whom you communicate monthly or less frequently are your extended ties. In addition, you may sometimes correspond on a one-time or short-term basis with friends of friends, or others you have "met" online (a weak tie link.)

An individual's social network—"the web of social relationships that surround an individual and the characteristics of those ties"[2] has long been recognized as an important factor in maintaining health and managing disease more effectively. Online communications within personal networks are now providing several new dimensions of social, informational, and logistical support for those facing health concerns.

Internet Surrogates

In the beginning, like most health professionals and researchers, my Pew colleagues and I assumed that patients would do their searching for themselves. So we were surprised to discover that more e-patients (81 percent) had gone online because a friend or family member had been diagnosed with a new illness than had searched the Net following a new diagnosis of their own (58 percent).[3] So in August 2003, the Pew online health team began asking not only about the searches our respondents did for themselves, but also about their experiences as *Internet surrogates*—e-patients who turn to the Net to help others.

Internet surrogates are typically ordinary people who have stepped in to help friends or family members who are facing a medical challenge. They may be caring for a sick, aging parent or a chronically ill child, supporting a newly diagnosed friend, helping a relative deal with a chronic illness, helping a workmate or a neighbor through a physical or emotional crisis, or making a terminally ill spouse more comfortable.[4] As one patient in an online focus group explained, “I am just a mom with absolutely no special training or education in health care, but because I have been able to access resources for information online, I have been able to truly play a part in our care.”

In the illustration below, reviewer Mark Bard of the Manhattan Research Group has identified several significant “zones of influence,” made up of those who benefit from the efforts of Internet surrogates.[5] Because of their efforts, the online health resources the Internet offers are now available to a significantly larger proportion of our fellow citizens than we had previously realized.

Updating the numbers above with more recent Pew Internet & American Life survey data yields the following:[6]

Active e-Patients	***93.0 million***
Children of e-Patients	***42.5***
Caregiver Impact	***16.7***
Spouse Impact	***14.3***
Total e-Patient Zone of Influence	***166.5 million Americans***

Women search on behalf of others more often than men do. The middle-aged do so more than the elderly. Many Internet surrogates not only search for static medical content on their friend or family member’s behalf, but also exchange e-mail (or other electronic communications) with expert patients and specialist clinicians they find online. The online efforts of surrogates have direct effects on medical care. Nearly half (46 percent) of the surrogates said that their last online search affected their decisions about how to treat an illness. Thirty-nine percent said that the information they found online led them to ask their loved one's doctor different questions, and 34 percent said that online health resources had changed their overall approach to medical care.[7]

Internet surrogates often serve as the key decision makers for our sickest and most vulnerable patients. Many use the information they have found online to help make sure that their loved ones obtain the best possible clinical care, accompanying them to doctor’s appointments, seeking online advice from expert patients with the same diagnosis, and consulting with medical specialists they have found via the Net. As one respondent wrote,

“Being informed makes it easier for me to be of support to my family and friends in a time of need.”[8]

Helping Patients and Families Deal with a New Diagnosis

When a new illness is diagnosed within a wired family, patients and caregivers reach out to their personal networks. When medical tests revealed that Ann Cochran’s 13-year-old son Harris had a life-threatening arteriovenous malformation in his brain,[9] the Washington DC communications consultant immediately “put out e-mail to friends and family. E-mail became an instant support group, family reunion, and advisory board. Friends of friends, relatives of friends, and friends of relatives wrote back: ‘We have friends at Mayo….’ ‘You have to see [Dr.] Cogen at Children’s….’ ‘We’re bringing dinner over on Friday night…. What’s Harry’s favorite dessert?’”

“In the beginning, our online health networks may be subsets of our larger and more extensive personal online networks,” says White Paper advisor Alan Greene, a Stanford pediatrician. “If I e-mail my friend Tricia to tell her that I’ve just been diagnosed with hypothyroidism, she may begin by doing a Google search for me. She may give me an online introduction to the best thyroid doctor in town. Or she may e-mail her friend Suzie who has become something of an expert on this topic after receiving a similar diagnosis last year. Tricia may then report back to me with the advice and resources Suzie recommends. Or she may put me directly in touch with Suzie herself (a new weak tie link).

"Suzie may then refer me to a good online support group for this condition. She may order me a good self-care book on our shared condition from Amazon. She may offer to accompany me to my next doctor’s appointment. Or she may retain the services of an online medical librarian service[10] to prepare a custom-tailored in-depth medical report providing exactly what I need to know about this disease.

"Because we're mutual friends of Tricia’s, and because we are now disease-mates, as well, there's frequently a surprisingly level of altruism in these relationships. So Suzie may take me on as a project, spending several hours and/or a good deal of money helping me understand what I am facing and helping me get the best possible medical care. And other members of my personal network may recommend additional resources. So my personal medical support network may grow and grow.

“People use their personal online health networks in very problem-specific ways,” Greene explains. “If I broke my wrist falling off my bicycle, I’d reach out to an entirely different subset of network members than I would if I developed a nasty case of eczema. And our networks are

always changing. Just yesterday, for instance, I met a pediatric gastroenterologist who would now be my number one choice as a source of online advice for anyone with a pediatric GI problem. And as the result of some negative feedback I recently received, there's another pediatric GI specialist I might have called or e-mailed yesterday who is now off my list."[11]

Patient-Centered Support Networks in Serious Illness

Many of us have been called upon to participate in the personal online health networks of friends and family members facing a serious illness. We all had sick friends in pre-Net days as well, but thanks to e-mail and other online resources, we can now become much more intimately, and helpfully, involved in their medical experiences and concerns than ever before.

Here's an e-mail message I received several years ago from my good friend Lenny Nagle, a Seattle psychotherapist:[12]

> **Dear Friends,**
>
> **Two or three months ago, Judy began to notice some small, painful bumps on her lower right leg. They gradually grew more intensely painful, and some become infected. They were finally diagnosed as Calciphylaxis, a rare condition found only in people with kidney disease.**
>
> **In this disease, the small arteries gradually become blocked by the buildup of calcium. If the blockage is extreme, the skin begins to die, exposing the tissue beneath and leaving it vulnerable to infection. If the infection or the blockage of arteries spreads to the vital organs, this condition can be fatal.**
>
> **Unfortunately, Calciphylaxis is extremely rare and is poorly understood. Commonly used treatments, none of which are clearly effective, include special phosphate binders, dietary management of phosphate and calcium, hyperbaric therapy, and surgical removal of the parathyroid glands.**
>
> **Judy is currently being treated with a drug that helps bind phosphate without the use of calcium (Renagel). She is on a diet low in both calcium and phosphate. She is going to St. Michael's hospital every weekday to spend two hours in a hyperbaric chamber, a room full of pure oxygen at high pressure. This forces oxygen into her affected tissues, hopefully promoting healing. It is a new treatment and has been successful in some cases, but it is still considered experimental. No decision has yet been made about removing Judy's parathyroid gland.**
>
> **We are e-mailing this information because we thought you would like to know—and because it is stressful and upsetting for us to have to explain this frightening disease over and over again to all our caring family members and friends. We will send further e-mail updates to keep you up to date on the latest developments.**
>
> **Love,**
> **—Judy and Lenny Nagle**

This e-mail message was sent to about a dozen close friends and family members in April 2001. Up to this point, Judy, a family therapist, had been dealing with several troubling chronic conditions, but this was the beginning of the end. Over the following sixteen months, Lenny

sent out a total of 128 "Judy Updates" to a list that ultimately grew to include more than 80 names—other family members and friends, new friends, health workers they met and befriended in the course of Judy's care, and a variety of others. As the mailing list grew, its members became the Nagles' *patient-centered support network*—a very different entity than the better-known *disease-specific online patient communities*.

When I asked Lenny what his regular "Judy Updates" and the community that grew up around them had meant to him, and to Judy, he replied as follows:

> **It was wonderful to be able to share our struggles and triumphs with a loving, supportive group of friends who were eager to hear from us. And since our friends knew exactly what we are going through, we knew that we were not alone.**
>
> **We didn't have to spend hours and hours on the phone, bringing everyone up to date. And since some of our medical experiences had been so unpleasant, it would have been upsetting to relive them over and over.**
>
> **Some list members combed the Internet for information that could be helpful in managing Judy's illness, providing information, referrals, and advice. The physicians on the list sent medical information and advice. Others volunteered to drive Judy to appointments, take her out to lunch or shopping, cook her dinner, give her massages, or take me skiing or sailing. Others provided continuing emotional support, while many friends who lived nearby helped me coordinate the day-to-day details of home care, housecleaning, grocery shopping, hospital visitation, and transportation to medical appointments. Others sent flowers, candy, books, cards, and other gifts. The support we received was incredible.**
>
> **During the last year of Judy's illness we weren't able to socialize much. So it was great to know that we hadn't fallen off the radar screens of our many friends with their busy lives.**
>
> **333**

Additional excerpts, completing the story, can be found at the end of this chapter.

Websites that Support Patient-Centered Online Health Networks

A variety of other online systems has evolved to support private online health networks for the seriously ill. Dr. Rochelle Stark was halfway through her general surgery residency at St Francis Medical Center in Trenton, New Jersey, when she was diagnosed with Hodgkin's lymphoma. She now uses the services of Care Pages (http://www.carepages.com), a service provided by her local hospital, to share her journey through the long process of diagnosis and treatment with friends and family. While most Care Pages are private, Dr. Stark has volunteered to make her Care Page public, helping colleagues and other patients appreciate the importance and potentials of patient-centered networks for those facing a serious illness.

The *Patient Update* section of her home page offers regular updates on how she is doing and feeling, while the *Message Board* section contains dozens of publicly accessible messages—from friends, family members, local medical colleagues, hospital and clinic staffers, as well as from many cancer patients who have found her via the Internet. A Photo Pages section is also available.

Why Personal Online Health Networks Have Received So Little Attention

We found many examples of personal networks that had become patient-centered networks. But we found very little scholarly writing on this topic. At one point, we turned to a professional online researcher at Google Answers (http://answers.google.com) for help. You can read our original question and all searches and discussion relating to those searches at: **http://answers.google.com/answers/threadview?id=474017.**

At one point, our Google Answers researcher commented: "I was intrigued by your question because I have personal experience in using [this type of] online resource [myself]. Last year it became clear that my parents needed a great deal of help with some health problems… I set up a Yahoo! Group for my four siblings and me… All five of us were determined to pitch in with getting through the crisis but we all had many other obligations. [With the private group] we could pool all the information we were gathering and make it all available… so we wouldn't have to keep repeating the same information, play telephone tag, or keep track of the huge volume of e-mail we were generating. [With] all of the correspondence in one place, we could easily monitor who was doing what. We had a calendar showing all the medical appointments and posted complete lists of my parents' doctors and medications. When anyone researched a topic, they posted the results and the appropriate links... I don't know how we would have gotten through this crisis if we had to handle all the coordination by phone."

Despite this experience, he was unable to find other examples of similar use of online resources. He admitted the search was extremely frustrating. It seems likely that other families are doing something similar, but "it's just not documented."
One reason for the seeming invisibility of such networks became apparent when I asked our researcher to share some anonymized content from his family's private mailing list with our White Paper team. He responded:

> **As for my family support group—I'm afraid it's much too personal to share even with guarantees of anonymity. The five of us still use it whenever something comes up. We have not opened it to anyone outside**

> **our sibling circle—not even spouses or children.**

Yahoo Groups currently hosts more than 12,000 different online discussion groups for "individual families".[13] These groups are accessible to members only. No studies have been done and no statistics are kept to suggest how many of these are currently being used, either exclusively or nonexclusively, to manage a loved one's illness. We wrote to a dozen groups that appeared, from the brief public group description, to include family caretaking among their functions. But none were willing to share their online caregiving processes with readers of this White Paper.

Providing Continuing Support for the Incurable

Professional medicine is often at its worst in providing continuing comfort and care for patients facing serious illnesses that are beyond the hope of cure. In such cases, the support and care patient-centered networks of family and friends provide can be a lifeline:[14]

> **I had a moderately bad CT scan this week for the first time in a year: One of my old liver tumors is growing again, and a new one has appeared. We're seeing my main oncologist on Friday and will decide then what the new regimen will be, but it's almost certain to be a harsher chemo. All this stuff is cumulative, so I can only say that it pretty much sucks. I'd feel a lot better if my insurance policy (which is otherwise exemplary) covered Ben & Jerry's Karamel Sutra ice cream, my major Food Group these days.**
>
> **But the bigger picture is that this week marks the third anniversary of my diagnosis. I hated the first anniversary, which mostly focused my attention on all the misery involved and the endlessness of this whole slog. Now, of course, I've come around to thinking that endlessness is a pretty fine concept, and I can even celebrate these anniversaries as markers of luck and cunning, or whatever the hell it is that's been sustaining me all this time.**
>
> **But the biggest reminder this anniversary brings is just what good fortune I have in the amazing web of love that has fed us, amused us, driven, listened, embraced, carpooled, and emailed. There's so much mystery in this whole deal, where this cancer comes from and how I've managed to stay alive—to flourish, even—for so long. The one thing I do know is that there's no chance I would have had such a lush life, these last three years, without my friends.**

What We Can Learn from Patient-Centered Networks

In exploring online patient networks even to the limited extent possible in the course of preparing this White Paper, we felt that we were offered a valuable glimpse into Information Age healthcare in action. Patient-centered networks are already providing a great deal of invaluable support and care to millions of e-patients across the globe. Understanding patient networks can help clinicians understand how many contemporary e-patients are now capable

of managing much more of their own medical care, of providing useful support and care for other patients, and of providing useful feedback and guidance for clinicians.

Patient networks are emerging as a new medical domain within which a wide variety of individuals and groups become valuable healthcare resources. Nearly all of those involved with patient-centered networks provide their services for free, and since patient-centered networks operate independently of the formal healthcare system, they are not constrained by that system's built-in limitations, inefficiencies, and defects. They often approach the problems they face with a striking degree of innovation and creativity.

"Our personal online health networks function as a social version of our body's immune system," Alan Greene concludes. "Different parts of it spring into action in response to a variety of different medical challenges. And without it, we would be at much greater risk of needless cost, suffering, morbidity, and mortality."

Although patient-centered networks have not been well studied, they are beginning to show up in popular culture. Cartoon strips such as Doonesbury and Funky Winkerbean have depicted characters using personal groups to inform family and friends of medical occurrences. The importance of this form of communication should not be overlooked, since individual telephone calls and emails to a person's entire network of concerned individuals puts a huge burden on the patient or the caregiver. ■

Lenny Nagle: Additional Excerpts from his "Judy Updates"

Judy seems quite happy most of the time. Considering what she's been through—and what lies ahead—her serenity and grace continues to amaze everyone.

I recently asked her how she keeps her spirits up. She told me that when she finds herself feeling frightened or depressed, she recites the following meditation: "May I be filled with loving kindness. May I be well. May I be peaceful and at ease. May I be happy." And after she focuses on herself for a while, she will focus on me, on our son Will, or on other family members or friends who are having a difficult time. She says this is very helpful, and usually calms her fears and fills her with a deep sense of peace.

*** * ***

We are beginning to prepare for Judy's death. We've both experienced lots of sadness and crying as we prepare for the time when we can no longer be together. But the same time our love seems to have an incredible tenderness and purity. The suffering we are going through is like a crucible burning off all the old power struggles and resentments—all the stuff that most couples get caught in. What's left seems pure, achingly tender, and incredibly comforting.

*** * ***

After conferring with our doctors, we all agreed that Judy had already stayed as long as she could manage and that it was time for her to go. I decided to bring her home to die. Two big burly gruff paramedics brought her home in an ambulance. They treated her with great delicacy and respect. The house was filled with flowers from the gardens of friends. About a dozen of her closest friends where there. Will had put up photos of Judy when she was young, strong, healthy and breathtakingly beautiful.

When we arrived home, Judy was calm, breathing easily, but deeply asleep and unable to respond, except that whenever I would kiss her, she would give me a wonderful sweet kiss back.

*** * ***

A little later her breathing changed into a labored rapid rhythm. It was upsetting to us all because she looked uncomfortable. Then my brother-in-law, Ben, arrived. He's an intensive care doctor who has made dying comfortable for hundreds of patients. He assured us that Judy was not uncomfortable. Her tongue was so relaxed that it was slipping back in her mouth and partially blocking the airway. Following the hospice nurse's instructions, I increased the dose on her morphine pump. Judy became more and more relaxed. She began to breathe more slowly, with a pause between breaths. The pauses grew longer and longer. Finally her breathing stopped. She looked like Michelangelo's Pieta, still as a statue, incredibly peaceful and beautiful. If there is such a thing as a good death, Judy had one.

*** * ***

The supportive and caring functions of the group did not end with Judy's death. The mailing list continued for more than a year thereafter, providing Lenny and the other group members with a way to explore and share their feelings about Judy's death and to provide each other with continuing support and care.

A part of me had been preparing for Judy's death ever since I fell in love with her. She'd had a serious kidney infection as a child, and when I was eighteen, a physician friend told me that her kidneys would probably fail—and she would die—in her early thirties. He was right. But by the time her kidneys failed, dialysis and transplantation were available. Yet, as she came to the brink of death time and time again, I got more and more used to the idea that the day would come when she would die and I would go on without her. But even though I knew, on some level, that my life would continue after Judy died, our hearts and lives were so closely joined, and she was such a central focus of my life, that another part felt that I could not possibly survive her.

Yet now she is gone and I am still here. And as the stress of worrying about her and caring for her illness fades, I can feel that my energy is gradually becoming available for other things. I have been watching what I eat and have losing weight. My enthusiastic nature has not yet fully reasserted itself. But I can feel it beginning to stir.

C. Haythornthwaite, *A social network theory of tie strength and media use: A framework for evaluating multi-level impacts of new media*, (Champaign, IL: University of Illinois, 1999). www.alexia.lis.uiuc.edu/~haythorn/sna_theory.html (Accessed on Jan. 25, 2006)

[2] Lisa F. Berkman, and Thomas Glass, "Social Integration, Social Networks, Social Support, and Health," in *Social Epidemiology*, Lisa F. Berkman, and Ichiro Kawachi, Editors, (New York: Oxford University Press, 2000), p. 145.

[3] Susannah Fox, "Callback survey of 500 previously identified health seekers," *Pew Internet & American Life Project*, Mar. 27, 2004, [Susannah--Is this correct?] personal e-mail to author (Accessed on May 12, 2004).

[4] Susannah Fox, "Callback survey of 500 previously identified health seekers," *Pew Internet & American Life Project*, Mar. 27, 2004, personal e-mail to author (May 12, 2004).

[5] Manhattan Research, "The Future of e-Health: Looking for Signs of Life after the Internet Bubble," 2002. <http://www.manhattanresearch.com/signsoflife.pdf> (Accessed on May 1, 2003).

[6] Updated numbers are based on 2002 US population.

[7] Susannah Fox and Lee Rainie, "Vital Decisions: How Internet users decide what information to trust when they or their loved ones are sick," Pew Internet & American Life Project (May 22, 2002). Available at: http://www.pewinternet.org/PPF/r/59/report_display.asp (Accessed on July 22, 2006.)

[8] This and other e-patient quotes are taken from an unpublished qualitative e-patient survey conducted for the Pew Internet & American Life Project by Tom Ferguson and Susannah Fox, May-August 2002.

[9] Ann Cochran, "I'm Sorry to Tell You This, But..." *The Washingtonian*, April 2003. http://www.washingtonian.com/health/braindisorder.html

[10] For one example of an online medical librarian service, see White Paper advisor Jan Guthrie's The Health Resource (http://www.thehealthresource.com).

[11] Personal communication, Alan Greene, Sept. 14, 2005.

[12] Names and identifying characteristics have been changed to protect both the patient and the caregiver's identities. Lenny Nagle's e-mail messages have been edited for spelling, punctuation, and readability. Used with the kind permission of "Lenny Nagle."

[13] Yahoo Family Groups. http://dir.groups.yahoo.com/dir/Family_Home/Families/Individual_Families (Accessed on Jan 24, 2006)

[14] This e-mail was received on July 15, 2004, by one of our advisors, a member of the mailing list of friends and family members supporting the author through her long illness. It is reprinted here with the kind permission of the author, who has asked to remain anonymous.

Who then can so softly bind up
the wound of another as he who
has felt that same wound himself?
—Thomas Jefferson

CHAPTER FOUR

The Surprisingly Complex World of e-Communities

In addition to searching the Web and e-mailing friends and family, many e-patients, especially the ones suffering from acute and chronic conditions, are using the Internet to communicate with other patients, family caregivers, and patient support groups they've found online. A participant in a Pew online focus group explained that after he was diagnosed with emphysema, his doctors told him there was nothing further they could do. They would make him as comfortable as possible, but he would die very soon. Unwilling to accept such a dire prediction, he searched online for further guidance and was welcomed by the members of an emphysema support group called Efforts.[1] Several group members went out of their way to help him get the information he needed to understand his disease and its treatments. They also encouraged him to become more involved in his care and to take a higher degree of control of his illness. He now spends eight hours a day online, learning everything he can about his condition, reviewing the latest studies and treatments, and offering support and guidance to other patients.[2]

Online Support Communities

A 2001 Pew Internet study on online communities (e-communities)[3] found that 28 percent of U.S. adult Internet users, about 34 million people, had used the Internet to connect with an online support group for a medical condition or a personal problem. The Internet now offers health e-communities for almost every imaginable medical challenge—from AIDS and breast cancer to depression and stress, from weight loss and smoking cessation to parenting issues and elder care.[4] Every day, millions of e-patients from around the world communicate with each other via their health e-communities. A single cancer site, ACOR.org, sends out more than 1.5 million e-mails weekly via its health e-communities.

Health e-communities are a subset in a much larger universe of online groups that are devoted to topics ranging from politics and sports to news, city government, and religion. Eighty-four percent of all U.S. Internet users—about 90 million people—have participated in some type of e-community, and 66 percent (80 million) stay in regular touch with one.[5] As the facilitators of many such groups have learned, people often turn to e-communities during a

time of crisis. In the aftermath of the September 11, 2001, attacks, a third of American Internet users read or posted material in online chat rooms, bulletin boards, or other online forums.[6] The explosion of social networking sites where people can share daily stories, pictures and friends is starting to generate new, specialized health-oriented Web-based e-communities. It thus seems likely that as the Google generation ages, many more e-patients will turn to medical e-communities when faced with a medical crisis.

This is Crazy! This Information Needs to be Saved!

In 1995, Gilles Frydman's wife learned that she had breast cancer. "Our doctor looked at Monica's biopsy and said, 'You have nothing to worry about,'" Frydman recalls. "'Your wife has *ductal carcinoma in situ*, and we are going to take her breast and lymph nodes away. We will give her prophylactic chemotherapy. And just to make sure, we'll do a brain scan, a liver scan and a bone scan. But there's really no reason to be concerned. It's all quite routine.' Our entire conversation lasted about ten minutes." Frydman, who is delightfully and unmistakably French, gives a classic Gallic shrug and rolls his eyes at the ceiling.

"This lady was one of those doctors who gives the medical profession a bad reputation by trying to play God," he explains. "She took it for granted that she knew everything there was to know while we knew nothing. So she would give the orders and we would obey without question. There was no need to discuss alternatives."

Fortunately, Frydman knew his way around the Internet. "As soon as we got home, I went online, found a mailing list for breast cancer, and jumped right into the conversation: 'This is what our doctor recommends. Is this reasonable?' The answers to my questions came flooding back. After four hours, I knew that this was a complex type of cancer, for which an expert reading of the pathology was particularly important. Group members mentioned the two top pathology experts in the country. One of those experts just happened to be in New York where we reside." Frydman arranged to see a breast cancer specialist in his medical institution within a few days.

"The doctor at Sloan-Kettering could not have been more different," Frydman says. "He showed us the test results and explained what they meant. He listened attentively to my wife's questions and worries about the potential impact of treatments on her quality of life. He reviewed our situation in great detail and confirmed what we'd learned from the e-community: The treatments our other doctor had recommended were seriously out of date. There was no need for chemotherapy and no reason whatsoever to do a radical mastectomy. The state-of-the-art treatment for Monica's cancer had been, for at least ten years, lumpectomy, and

radiation. Our new doctor said he was reasonably sure this was the only treatment she would ever need. And so far, that's turned out to be true."

During his wife's treatment, Frydman became an active participant in the BREAST-CANCER e-community. He was impressed by the quality and complexity of the information it provided and by the dedication of the list's organizers and core group of active members, but he was horrified to discover that once messages were sent out to subscribers, they weren't saved and archived.

"All this material, which had been so valuable for us, was simply disappearing," Frydman says. "Once it went out to the group, it was gone forever. I talked to the list owner and discovered that he was hosting the community on a university server. They hadn't the storage space or the manpower to create such an archive. So, I said to Monica, 'This is crazy. This information needs to be saved. I'm going to start a website to make that happen.'"[7]

Once he had the breast cancer list archive up and running, he began contacting the facilitators of other cancer support groups, offering to archive their list messages in searchable form. Within six months the original idea evolved into the establishment of a nonprofit organization, the Association of Cancer Online Resources (ACOR), and a website (http://www.acor.org).

Eleven years later, Monica Frydman is doing well, and the website her husband created has become the world's largest online community for cancer patients. The ACOR site currently hosts more than 150 cancer-related public e-communities; most of them support groups for specific types of cancer. A statement on the home page hints at the site's importance for cancer patients worldwide: "ACOR delivered 1,524,367 individual e-mails across the globe last week." Most of the ACOR e-communities have been in existence for a decade or more and keep on adding new members almost daily, demonstrating the benefits they provide to their members. In the early years, many health professionals considered those e-communities to be potentially dangerous, but recent peer-reviewed studies show how those self-organized groups enable their members to properly assess the quality of the information received and correct potentially harmful information in a matter of hours.[8]

Braintalk: An e-Patient-driven Online Educational and Support Community

The field of neurology rather than oncology provides another interesting example. In 1994, a team of Massachusetts General Hospital (MGH) researchers examined how people with neurological concerns were using the Internet. Even at that early stage of the Internet's

evolution, they found that e-patients with epilepsy, multiple sclerosis, Parkinson's disease, and many other neurological concerns had already created an impressive variety of online health resources.

"We found their online support groups especially intriguing," said White Paper advisor Dan Hoch, MD, a co-director of the project. "They offered their members more convenient, powerful, and complex information and support than any of the face-to-face groups we had seen."

"Those early online groups really impressed us," said White Paper advisor John Lester, the project's other co-director. "But they were scattered across dozens of different servers. There was little communication or coordination among different groups. There were groups for some neurological concerns but none for others, and there was no easy way for patients interested in forming a new group to find one another." So in March 1995, Lester, Hoch, and their colleagues Stephanie Prady and Yolanda Finegan instituted a family of online groups called the BrainTalk Communities (http://brain.hastypastry.net/forums/) to support e-patients with neurological concerns.[9]

Building from the Bottom Up

Many professional efforts to develop resources for e-patients have taken the traditional "doctor knows best" approach, providing professionally-created content in a top-down manner. "In these provider-centered systems, patients and caregivers have little or no input or control," Lester says. "Yet the communities we'd observed—in which patients had complete control—appeared to be doing quite well without professional assistance."

"We decided that we would think of ourselves as architects and building contractors," Hoch recalls, "creating an online system in response to patient requests. Our ultimate goal was neither to direct nor monitor our e-patients' activities. We simply wanted to help them do whatever they wanted to do. So instead of specifying the topic areas and designing the underlying IT structure ourselves, we asked patients what they wanted and designed the system by following their suggestions."

The MGH team launched the project by establishing basic discussion groups for epilepsy (Hoch's specialty) and 34 other health topics, from Alzheimer's disease to Tourette's syndrome. The forums were open to the public and originally were not moderated by the developers. Users were coached in the use of the online systems—and were then encouraged to moderate the discussions and manage the site on their own. Today, the BrainTalk Communities host more than 300 support groups devoted to neurological diseases and

disorders. “BrainTalk is a user-driven or ‘bottom up’ community space,” Lester says. “Patients, not professionals, provide the content and make and administer the rules. They do a very impressive job of maintaining the communities and helping one another. And by outsourcing most of the management, content development, and day-to-day operations of the site to patients, we’ve been able to keep our expenses to a minimum.”

After nearly a decade of e-patient research, the MGH e-patient team has concluded that people’s online behaviors are considerably more complex—and more social—than most health professionals realize. “A typical MS patient might say, ‘Okay, first I’m going to check my e-mail—including my mailing list messages—and respond as needed,’” Lester explains. “‘Then I’ll go see if there are any new messages on my three favorite bulletin boards and maybe post a few comments. Then I’ll check my favorite chat room to see who’s there. Then I’ll check my MS buddy list to see who’s online right now and invite some friends to join me there. After that I'm having lunch with Matt, an MS-er from California. I know him well from the group, but I’ve never met him before face-to-face. And after lunch I need to go online to read the latest issues of the three main medical journals for MS so I can summarize the key articles for my support group.’”

Online Groups Supplement, but Don’t Replace, Doctors

In one of the earliest studies of e-patients who were members of an online support community, developer Bill Kelly and I surveyed 191 active members of a family of online support communities, which have since become a part of WebMD.[10] Most of our respondents were seriously ill patients dealing with conditions like breast cancer, prostate cancer, ovarian cancer, and hepatitis C. We asked them to tell us which of the following three resources—their specialist physicians, their primary care physicians, or their online support groups—they would rate most highly in of the following categories:

Best in-depth information on my condition
Best practical knowledge of my condition
Best technical knowledge of my condition
Best for helping me find other medical resources
Best in helping to diagnose my problem correctly
Best in helping and advising on management after diagnosis
Most compassion and empathy
Best in helping with emotional issues
Best in helping with issues of death and dying
Most convenient
Most cost-effective
Most likely to be there for me in the long run

Online groups ranked significantly higher than either generalists or specialists for convenience, cost-effectiveness, emotional support, compassion/empathy, help in dealing with death and dying, medical referrals, practical coping tips, in-depth information and “most likely to be there for me in the long run.” By a narrow margin, online health communities were also rated as the best source of technical medical knowledge. Specialist physicians were rated highest for help in diagnosing a condition correctly and for help in managing a condition after diagnosis.

To say that these results surprised us would be an understatement. We were stunned, even dumbfounded. Clearly the value of these groups, as perceived by the patients, was substantially greater than most health professionals had realized. Yet it appeared that online groups had relatively little to offer in some areas (e.g., the process of diagnosis). Doctors’ abilities to advise patients on an ongoing treatment plan were also highly valued, as were their technical knowledge and their capacity for support and empathy. In fact, it appeared that doctors were best at what the groups were worst at, and vice versa. We concluded that the combination of a good doctor and a good support group might offer e-patients the best of all possible worlds.

Forgotten Heroes

I'm thinking of a particular type of medical worker. See if you can guess.
Average wage? Zero. Benefits? Zip. Medical insurance? None. Social Security? Not covered. Time off? Hardly any—most offer services 24/7, 365 days a year. Training? Nonexistent. Job candidates must learn everything on their own. Skills required? Healthcare administration, clinical care (specialization required), a full range of nursing skills, and the ability to deal with a patient's complex emotional needs. Duties? From high-level research and complex medical management to bathing, providing personal care, and emptying bedpans. Must be willing to do paperwork while patient sleeps.[11]

Give up? Family caregivers of those with debilitating, chronic illnesses are the most numerous—and overlooked—health workers of all. There are 27 million in the U.S. alone. They outnumber all other types of health workers combined by a factor of four to one, yet they often feel like the odd person out when dealing with hospitals and medical professionals. In the world of Health e-Communities, they are first-class, not second-class, citizens and frequently serve as the group's mainstays, organizers, and hosts. As Karen Parles notes, "Traditional healthcare all too often leaves friends and families out of illness care. But spouses and other caregivers have so much to offer. Online support communities don't make this mistake. We recognize family caregivers as full-fledged senior partners and give them the opportunity to draw on their full range of abilities for the common good."

Evidence of the Effectiveness of Online Groups

A number of studies[12] have described how online support groups operate, how they can be of use, and who can benefit from them. But most have been published in the social sciences literature and are rarely read by clinicians or cited in mainstream medical journals. Some of the medical researchers who have attempted to evaluate these groups have projected their professionally centered perspectives into the evaluation even though they may not apply, co-opting lay self-help models and developing professionally led therapy groups instead. Professionally run groups and patient-run groups exhibit very different dynamics: Professional groups may discourage patient initiative and assertiveness and run the risk of becoming little more than shared "ask the doctor" sessions. Patient-initiated groups that have emerged out of patients' own efforts can be considerably more effective at stimulating patient initiative, empowerment, and autonomy.

The studies done to date, even those considered breakthrough research,[13] have only started to scratch the surface. Much of the most fundamental research in this area remains to be done. No one knows how many health e-communities exist. There are not even any good ballpark estimates. No one knows what might happen if top medical specialists for a specific condition and a robust and active patient-developed online support group for the same condition were encouraged to collaborate in innovative ways.

To help medical professionals gain a better understanding of the benefits online communities can provide, we asked the advisors to identify some of the most important benefits of online support groups. Here's what they came up with.

Putting a Human Face on Medical Information

Online groups, and the materials they provide, often do a better job than medical professionals of providing patients with the information they really want and need. Learning you have a new disease can be an earth-shattering experience. One day you're "normal" and the next you're a "patient," perhaps for life. Connecting via an online support group with dozens or hundreds of others who have the same condition can be immensely comforting to newly diagnosed patients. In many cases, such patients have never encountered anyone else with their condition.

Sharing concerns can go a long way toward demystifying and (as one e-patient wrote) "de-awfulizing" your condition. It can help you reframe the experience, from a unique catastrophe affecting you alone to a widely shared experience with which many others are currently living and coping—and many quite nicely. In the outside world, your diagnosis can sometimes seem a frightening misfortune that sets you apart from others. In your online community, it is a badge of honor that connects everyone together.

Practical Day-to-Day Illness Management Advice

Where online communities perform best is in providing members with answers to their practical day-to-day questions. As Karen Parles explained, "With the nonsmokers in the group, I discussed issues related to having a smoker's disease and what it's like to feel invisible within a stigmatized group. I found young mothers with whom I could share that particular heartache of raising small children while fighting a deadly disease. Group members advised me on a hundred matters, from where to buy a wig after chemotherapy to what type of pillow to use after surgery. And several fellow-patients offered useful advice on how to handle the medical paperwork and insurance issues that are a major source of stress for every cancer patient. This sharing of practical or 'non-clinical' information is typically

seen by both patients and physicians as outside the physician's purview. This comprehensive support for the ongoing day-to-day challenges patients face is something no professional could possibly provide."

e-Groups Are Always There

Many patients can no longer count on seeing the same doctor when they need medical care. Physicians come and go. People switch jobs or lose them altogether. Employers drop or switch their healthcare coverage. Even those who are able to go back to the same facility can't always see the same clinician. It can be comforting indeed for a patient with a serious illness to know that they have 24/7 access to a concerned group of knowledgeable long-term friends who will come to their aid if needed. Online groups that preserve earlier postings as searchable archives can multiply their effectiveness by making the wisdom of their members available to many others, far into the future. Newcomers can search these archives intensively after a new diagnosis, finding much useful information and equipping themselves with an in-depth introduction to the group and its members.

Providing Continuing Support for the Incurable

Professional medicine is often at its worst in providing continuing comfort and care for patients facing serious illnesses that are beyond the hope of cure. In such cases, the support and care provided by patient-centered e-communities provide can be a lifeline.[14] Years of conversations taking place in some of these specialized end-of-life e-communities show their great effectiveness by providing supportive environments where patients learn to deal with the many fears usually associated with death.

Special Benefits for Those with Rare Conditions

"There's no medical condition too rare to benefit from an online support group," Gilles Frydman says. "The rarer the disease, the more valuable and cohesive such groups tend to be. Patients with rare cancers are often the first example of this disease their local oncologist has ever seen. So, most doctors aren't up-to-date on the latest treatments. e-patients can learn about the treatments currently in use at the leading treatment centers from their online communities. And they can then pass this information on to their physicians."

Online groups currently improve the quality of patient care across a wide range of diverse geographic, economic, political, and cultural circumstances. As Karen Parles notes, "Some of the most active members of our Lung-Onc list come from rural areas with limited access to

professional care. Two of our Australian members have a rare form of lung cancer their doctors had never seen and knew little about. Their physicians were very grateful for the treatment information the list supplied. There's so much we can do to help doctors and patients in foreign countries get up-to-speed on the latest treatments available in the US."

A Godsend for Those with Limited Access to Professional Care

As a member of the Brain Talk Communities (http://www.braintalk.org) recently observed, "When I talk to my doctor, I hear myself asking questions that my online 'family' needs to know. It's as if all these other people—the members of my group—are asking questions through me. And whatever answers I hear from my doctor, I know I'll share with them online."[15]

Gilles Frydman concurs. "Some uninsured and offshore members of our lists have told me that their online support group is their only source of medical guidance. Many English members of the breast cancer groups learned about Herceptin years before the anti-cancer agent was approved in the UK. A number of foreign patients suffering from gastrointestinal stromal tumor (GIST) learned about an early-stage European clinical trial long before the trial was listed in the NCI's PDQ database. Such help is increasingly available to the unwired." Karen Parles agrees, "A number of my fellow-members at the Lung-Onc group don't own computers themselves. They link to the Internet via their public libraries."

Keeping Up on the State of the Art for Your Condition

"Health e-communities can provide a comprehensive picture of the range of treatments currently recommended at treatment centers across the country and around the world," Frydman explains. "One of the main benefits for members is the ability to learn about the 'state-of-the-art' treatments available at the top treatment centers. We find that, in many cases, local clinicians have not mentioned these treatment options to their newly diagnosed patients. One would think this would be malpractice, but actually it is quite common.

"When patients in our groups discover that the treatments offered at the top centers are identical to those that have been recommended by their local clinicians, they are always greatly reassured," Frydman says. "When patients find evidence suggesting that they've been receiving sub-optimal care, they will usually share it with their clinicians. In many cases, they are able to convince their doctors to upgrade their services. But if the doctor is unable or unwilling to do so, they will frequently decide to exercise their right of free choice and will go to one of the top specialists or treatment centers that specialize in their type of cancer. When

speaking of rare cancers, there are frequently just a handful of real specialists. These top experts often tell me that e-patients are now coming from across the country and around the world to consult them, with a constantly growing percentage of patients visiting the specialists following an Internet-based self-referral. The experts greatly appreciate the high level of knowledge among our members, for they much prefer to work with well-informed, highly-motivated e-patients who can proactively share the burden of medical decision-making." ■

<http://www.emphysema.net> (Accessed on March 18, 2007)

[2] Susannah Fox and Lee Rainie, "Vital Decisions," *Pew Internet & American Life Project,* May 22, 2002, <http://www.pewinternet.org/reports/toc.asp?Report=59> (Accessed on Aug. 9, 2004).

[3] John B. Horrigan, Lee Rainie, Susannah Fox, "Online Communities: Internetworks that nurture long-distance relationships and local ties." *Pew Internet & American Life Project*, Oct. 31, 2001, <http://www.pewinternet.org/reports/toc.asp?Report=47> (Accessed on May 22, 2004).

[4] For representative listings of online support groups, see the following: http://selfhelpgroups.org (Accessed on Aug. 10, 2004)

http://directory.google.com/Top/Health/Support_Groups/ (Accessed on Aug. 10, 2004)

http://www.geocities.com/HotSprings/1505/medicalchats.html (Accessed on Aug. 10, 2004)

http://www.noah-health.org/english/support.html#Adrenal%20Disease (Accessed on Aug. 10, 2004)

http://www.supportpath.com/ (Accessed on Aug. 10, 2004)

http://psychcentral.com/resources/ (Accessed on Aug. 10. 2004)

http://www.geocities.com/HotSprings/1505/cancerchats.html Accessed on (Aug. 10, 2004)

[5] Horrigan, et al., op. cit.

[6] Ibid.

[7] The account of Frydman's early experiences with a breast cancer mailing list draws on Nancy Keene, "Meet Activist Gilles Frydman," *Patient-Centered Guides,* 2001, <www.onconurse.com/news/activist_gilles.html> (Accessed on Feb. 2, 2004).

[8] A. Esquivel, F. Meric-Bernstam, and E. V. Bernstam, "Accuracy and self correction of information received from an internet breast cancer list: content analysis." *British Medical Journal,* 332 (2006):939-42.

[9] John Lester, Stephanie Prady, Yolanda Finegan, Dan Hoch, "Learning from e-patients at Massachusetts General Hospital," *British Medical Journal*, 328 (May 15, 2004): 1188-90. <http://bmj.bmjjournals.com/cgi/content/full/328/7449/1188> (Dec. 2, 2006).

[10] Tom Ferguson, "E-Patients Prefer eGroups to Doctors for 10 of 12 Aspects of Health Care." *The Ferguson Report*, no. 1, Mar. 1999,

<http://www.fergusonreport.com/articles/fr039905.htm> (Accessed on Aug. 6, 2004).

[11] The classified ad for family caregivers has been adapted from Carol Levine, "Forgotten Heroes: Family Caregivers," *New York Daily News*, Nov. 12, 2002, <http://www.nydailynews.com/news/ideas_opinions/story/34673p-32809c.html> (Accessed on Aug. 10,

2004).

[12] One of the most extensive bibliographies of social sciences literature studies on online support groups is maintained by Azy Barak, a professor of psychology at the University of Haifa (Israel), <http://construct.haifa.ac.il/~azy/refsupp.htm> (Accessed on Aug. 6, 2004).

[13] Barbara Rimer, Elizabeth Lyons, Kurt M Ribisl, J Michael Bowling, Carol E Golin, Michael J Forlenza, and Andrea Meier, "How New Subscribers Use Cancer-Related Online Mailing Lists," *Journal of Medical Internet Research*, 7 (2005): e32.

[14] This e-mail was received on July 15, 2004 by one of our advisors, a member of the mailing list of friends and family members supporting the author through her long illness. It is reprinted here with the kind permission of the author, who has asked to remain anonymous.

[15] Quoted from Tom Ferguson, "Medical Knowledge as a Social Process: An Interview with John Lester," *The Ferguson Report*, no. 9, Sep. 2002, <http://www.fergusonreport.com/articles/fr00902.htm> (Accessed on May 28, 2004).

> Consumers can be central to the [medical] research endeavor. We can be a catalyzing force for translating research into the services we desperately need, such as treatments, technologies to alleviate suffering, and clinical methods of dealing with the conditions.
> —Sharon Terry

> As more and more patients gather, process, and publish their own medical data and coordinate recruitment for clinical studies, the relationships between researchers and patients will change dramatically.
> —Gilles Frydman

CHAPTER FIVE

e-Patients as Medical Researchers

In a research laboratory at Tulane Medical School, Andy Martin studied tissue samples of *sinonasal undifferentiated carcinoma* (SNUC), one of the most malignant and poorly understood cancers. The same type of cancer was growing inside his head.[1] On his website,[2] Martin explained why he decided to forego the usual duties of a third-year medical student to do what he could to further our understanding of the disease.

When Martin first decided that he wanted to do research on sinonasal undifferentiated carcinoma, Dr. Tyler Curiel, Tulane's chief of Hematology/Oncology, hesitated. All previous attempts to grow SNUC cells in the laboratory had failed. And Martin was already critically ill. But Curiel was so impressed by Martin's commitment that he helped raise funds for his research.

In the limited time he's had to spend on the project, Martin has exceeded everyone's expectations. He's figured out a way to grow SNUC cells in the laboratory, something none of the world's thousands of cancer researchers, most with M.D.s, Ph.D.s, or both, had been able to do. And growing cells in culture could be the first step toward finding a cure.[3] When Martin, after a number of failed attempts, finally succeeded in getting the tumor cells to grow, "It was one of the greatest moments of my scientific career," Dr. Curiel recalls. "I just sat right down on the lab floor next to the microscope, speechless."[4]

Martin felt lucky because other SNUC patients don't have the "extraordinary opportunity…to undertake a project that might have lasting benefit."[5] Dr. Curiel is now seeking funding to help other cancer patients to study their own tumors. "It's therapeutic for patients, especially those with hard-to-treat cancers," he says.[6]

From Passive Patients to Active Researchers

Andy Martin is just one of many patients who have made important contributions to medical science. But he's one of the few who have received credit for doing so. Many previously unexplained medical phenomena were first noticed by patients. But in most cases, professionals got credit for the patients' discoveries.

The sexual effects of Viagra (sildenafil) were originally discovered by patients. The British researchers who developed the drug were hoping that it would increase the cardiac blood supply in angina patients. Not until a number of patients took them aside to report the dramatic side effect for which the drug is now well known did the researchers begin to wonder whether the drug might increase another kind of blood flow. Thanks to this patient-provided intelligence, Pfizer soon found itself with a spectacular new pharmaceutical bestseller.[7]

The researchers can hardly be blamed for taking the credit. Before the Internet, the idea that patients might help advance scientific knowledge was almost unthinkable. And their opportunities to contribute were few. Most could play only a passive role, serving as subjects for clinical trials designed and conducted by professionals. Few if any researchers had considered the possibility that patients might be able to do real medical research—conducting experiments, collecting and analyzing data, and reporting significant and valid conclusions. Not until the Internet made it possible for large groups of patients with the same health concern to share their clinical experiences did the potential role of e-patients in medical research become apparent.

The Life Raft Group: A Research-Oriented Online Support Community

The man many consider the George Washington of e-patient-directed medical research is Norman Scherzer, a tall, graying public health professional who, until recently, worked with the Centers for Disease Control and the New York City Department of Health. Working from a tiny office in his New Jersey home, Scherzer now spends 50 hours a week coordinating his online group's research efforts, speaking soothingly yet authoritatively to worried cancer patients, and juggling a constant stream of incoming e-mails from group members, drug company executives, and cancer researchers, while a noisy fax machine clatters in the background.[8]

In 1994, Scherzer's wife, Anita, developed an abdominal tumor. "The first doctor we saw told us she had a rare type of cancer called leiomyosarcoma," Scherzer recalls. "So, I joined an online support group[9] for that disease. I found the mailing list discussions a little unwieldy at first. There were many different conversations going on. It was all so different from the

carefully organized information I knew from the world of public health. There was a lot of information available, but there wasn't much organization or quality control. It was a needle-in-the-haystack situation. But I persevered and soon figured out how to find what I was looking for. In the end, I *was* able to locate the needle we really needed."

Through the list, Scherzer heard that a cancer specialist at New York's Columbia-Presbyterian Medical Center was using a newly developed enzyme test to help diagnose tumors like Anita's. The Scherzers' own doctor knew nothing about it, so Norman took his wife to see this new physician. It was only then that Anita's cancer was correctly diagnosed as gastrointestinal stromal tumor (GIST).

At the time, no effective treatment for this condition existed. A few months later, however, Scherzer heard that a promising new drug called STI-571—which had produced excellent results with other cancers—would be tested on GIST patients. With the help of Anita's new oncologist, Scherzer enrolled her as patient number seven in an early 30-patient clinical trial. After only 30 days of this new therapy, now known as Gleevec, his wife's tumor had shrunk by more than 50 percent.

"After Anita was correctly diagnosed, I got in touch with some other GIST patients who'd started an online mailing list," Scherzer recalls. "There were only five of us at first, so we communicated by e-mail. But the group kept growing, and before we knew it, there were more than 50 members. It started to get a bit awkward, but by this time, I'd gotten to know Gilles Frydman, who runs ACOR (Association of Cancer Online Resources). With his help, we started a new mailing list for GIST patients. Since we would be using the new list to collect information on the drug's effectiveness, we decided to make it a closed group."

No Doctors Allowed

"Most online groups are open to anyone," Scherzer explains, "patients, family members, professionals, researchers, and anyone else who's interested. But our group meets privately and only GIST patients and their family caregivers can apply. We pre-screen them carefully, making sure they actually have this disease and understand that they'll be expected to share their medical experiences. We operate as a highly organized, networked scientific work group.

"The only health professionals admitted to the group are GIST patients and their family caregivers. Not even the top GIST specialists or the researchers who conduct the clinical trials in which many of our members are enrolled are admitted to the group," Scherzer says. "We publish an online newsletter[10] to share the things we're learning with the larger world."

While Scherzer is acknowledged as the leader, he is by no means the only one who makes this complex process work. In 2004 Scherzer explained, "We have our own medical librarian, who collects regular medical updates from each member and stores them on an Excel spreadsheet. We have a membership director who processes new applications and registers new members. We have an editor, a professional newspaperman who puts out a lovely newsletter every month. We have a webmaster, a treasurer, a list manager, and a government relations coordinator. And of course we have our Science Team.

"The Life Raft Group's Science Team is comprised of approximately ten highly committed members who regularly review the medical literature, speak with leading GIST specialists, interact with top medical researchers, keep up-to-date on the latest drug company information, and check in with other support groups in an ongoing attempt to understand the current state-of-the-art GIST therapy."

"It's quite a remarkable crew," Scherzer reflected. "We have a virologist, a microbiologist, a physician-surgeon, and a physicist who works at Los Alamos. One guy is a key player at the Human Genome Project. We operate at a level of technical complexity many professionals would have trouble keeping up with. We provide other members—and our newsletter readers—with an up-to-date, high-level review of the latest developments in GIST treatment, often based on a growing understanding of the molecular and genetic components of the disease." Science Team members communicate via their own private list.

I asked Scherzer how he collected such a capable cast of characters. "People who survive a life-threatening disease—one that should have killed them years ago—are often extraordinary people," he explains. "When someone figures out how to defy the odds and stay alive long

enough to find a promising new clinical trial, either the patient, the caregiver, or both usually turn out to be truly remarkable individuals."

Bypassing the "Lethal Lag Time"

"One of the great benefits of patient-initiated research is its speed," Scherzer said. "We can get lifesaving information out to the people who need it right away, much faster than professional researchers, who must go through many time-consuming steps. First you design your study. Then you arrange for funding. Then you must get everything approved—sometimes by several different committees. Then you recruit your subjects. So at last you can begin. Then you must wait for your results to trickle in. But that's only the beginning.

"Next you must analyze and interpret your data. You must write everything up. After all that, you'll need to find a peer-reviewed journal to publish your work. And if you're lucky enough to find one, you must go through even more long rounds of reviews, revisions, corrections, and proofing—as well as possible editorial or production delays. This can take several years. So professional research has a built-in lethal lag time—a period of delay between the time some people know about an important medical breakthrough and the time everybody knows.

"As a result of this delay, many patients who could have been saved by the latest treatments die unnecessarily. In my experience, this lethal lag time is rarely less than two to three years. And it can sometimes be four to five years, or even more. Physicians are subject to this delay just like everyone else. That's why we're so delighted that our newsletter mailing list is now growing just as fast among the medical community as it is among GIST patients."

Publishing Their Own Research Studies

In June 2001, the group used its newsletter to publish its first formal study of Gleevec's effectiveness for GIST patients In October 2001, the group published its first comprehensive study of Gleevec side effects. In addition to collecting the usual data, the Life Raft Group's study broke new ground in several areas:

- **It provided data on the quality of the clinical care available to study sparticipants at each of the centers conducting clinical trials.**
- **It attempted to evaluate the information sources patients relied upon.**
- **It developed a methodology by which patients could, in effect, serve as their own control group.**
- **It introduced a new scale for rating the severity of side effects from the patient's point of view, as opposed to the clinical trial toxicity standards established by the National Cancer Institute.**

This last point requires some explanation. Scherzer, trained to conduct population studies, looked for the existing instruments used to report the severity of side effects. They were available from the National Cancer Institute, but Scherzer was surprised to find that side effect measurements were focused exclusively upon toxicity as opposed to quality of life. For example, a patient who developed diarrhea several times a day, every day, on an indefinite basis would be rated a one on the NCI Toxicity Scale of one through five, with five being death and one being the mildest rating.As an alternative Scherzer adapted a common pain-management rating scale of one to ten, with one being the mildest and ten the most intolerable and then asked Gleevec patients to rate a number of possible side effects using this scale. A situation like the one described above would rank as an eight or higher using this scale.

To account for the fact that there was no placebo in the Gleevec clinical trial, Scherzer asked the survey participants to rank their side effects in the quarter prior to starting Gleevec in addition to each quarter following it. By doing so, he was able to document that some side effects were in fact pre-existing conditions. In addition, by rating side effects over several contiguous quarters he discovered that actual side effects got better over time. Finally, Scherzer correlated side effects by a number of demographic factors and discovered that they were generally worse in females, particularly those side effects that affected skin, and that the more severe they were at onset the more they improved over time.

When Scherzer presented the Life Raft Group findings on Gleevec side effects to the team that had just reviewed the results from the official clinical trial, the health care professionals could hardly believe what they had just witnessed. The results and the methodology were both extraordinary. This two-hour presentation was clearly a watershed; it resulted in significant funding for continued Life Raft Group patient-centered and patient-directed research.

“The new research model pioneered by the Life Raft Group is making it possible for patients and family members to contribute to clinical research for their diseases in unprecedented ways,” says George Demetri, Medical Director of the Center for Sarcoma and Bone Oncology at Boston’s Dana-Farber Cancer Institute, where he conducts clinical trials with GIST patients. “I predict that we’ll be seeing a lot more of this sort of thing in the years to come.” Dr. Daniel Vasella, CEO of Novartis, describes the research as follows: “The Life Raft Group … has provided various people, patients, doctors, investigators with a unique kind of data bank that cannot be replicated anywhere else, not even in patient trials.”

In 2007, The Life Raft Group website states: “Today, the Life Raft Group conducts its own patient-based research to ensure that it provides the most timely and relevant information that GIST patients need to survive. This internal research is designed to provide all of us with

information that is not currently available from clinical trials, either because we are waiting for the trial to reach its research end point and for the researcher to share the information or because the perspective of the trial is just not geared to what patients need to know in order to survive. We cannot completely depend upon the profit drive of the normal drug development marketplace and traditional research funding to ensure patient survival".[11]

Parent-Initiated Research on Reflux

When Caroline McGraw discovered that she and her husband Tom were going to have their third baby, she began to worry. Her two older children both had severe cases of gastroesophageal reflux disease (GERD), one with severe complications. Her doctor assured her that the condition could not be passed on from parent to child. The odds of her new baby having GERD were, he insisted, "a million to one against it." Eight months later, Caroline gave birth to twins. Both had GERD. And even though there was nothing in the medical literature to suggest that this trait was genetic, Caroline was convinced that it was. So she set out to prove it.

She joined forces with Elizabeth Pulsifer-Anderson, who had recently started PAGER (Pediatric/Adolescent Gastroesophageal Reflux Association), which offers an online support group and website (www.reflux.org) for this condition. Together, the two mothers took their idea of genetically transmitted heartburn to a number of medical research teams.

When the first researchers they met with turned them down, they gathered more evidence and tried again—and again. They finally succeeded with a research team headed by Christopher Post and Garth Ehrlich at Allegheny General Hospital's Center for Genomic Sciences. With the help of group members and an online screening process, the PAGER team found five families with multiple GERD sufferers spanning several generations. The researchers were able to evaluate both affected and unaffected family members, obtaining their detailed medical histories and performing genome-wide DNA scans. Finally, in the July 19, 2000 issue of the *Journal of the American Medical Association*, McGraw and Pulsifer-Anderson published the first study to demonstrate the patterns by which GERD is inherited and to map the responsible gene.[12] Caroline and Elizabeth, along with the key members of the medical research team, were listed as co-authors.

Jannine and Liz Cody

In 1985, Jannine Cody's daughter Liz was born with a cleft palate and her feet turned in. Her pediatrician told Cody that her baby had a rare genetic defect called chromosome 18 deletion

(usually written as 18-). Individuals with the condition are typically hard of hearing and mentally retarded. The doctor told Cody that there were only a few dozen known cases of 18- and that no treatments were available. He then opened a medical textbook, showed her photographs of a typical 18- patient, lying immobile in a froglike position and vegetative state, and told her that she must go home and persevere. Instead, Cody went to the medical library and started reading everything she could find on her daughter's condition.

At age four, Liz experienced a dramatic improvement in hearing. Cody attributed this to the fact that she had arranged for her daughter to begin taking human growth hormone (HGH) the year before. When her doctors told her that no such effects were known, Cody went back to the medical literature. After reviewing hundreds of scientific articles, she came across a study in which researchers had reported that cells from the brains of fetal rats responded to HGH by producing myelin, the protein that insulates the nerves. In people with 18-, this insulating nerve sheath is abnormal. Cody suspected that in addition to improving hearing, HGH might also help prevent the lowered IQ often seen in individuals with 18-.

When Cody told medical researchers at the University of Texas Health Science Center at San Antonio about her theory, they were impressed enough with it to test it. They found that she was right. Not only does HGH improve hearing, it increases IQ as well—by as much as 47 points. An increase of this magnitude means that, with proper treatment, children with 18-, who would otherwise have grown up mentally retarded, can now have normal IQs. The following year, Cody started a support group called the Chromosome 18 Registry & Research Society and began corresponding with affected families around the globe. She also enrolled at the University of Texas, pursuing a doctorate in genetics. Her group now sponsors the definitive website for the parents of children with chromosome 18 abnormalities.

In 1994, while still a graduate student, she developed and began offering the first effective treatment for 18- children. Today, Cody's professional bibliography includes more than three dozen peer-reviewed scientific articles, abstracts, and papers on chromosome 18 abnormalities. In addition to serving as president of the Chromosome 18 Registry & Research Society, Cody also serves as assistant professor of genetics and pediatrics at the University of Texas Health Science Center in San Antonio.

Portia Iversen Tackles Autism

In 1989, Portia Iversen won an Emmy as art director of the *Tracey Ullman Show*. Since 1995, when her firstborn son Dov was diagnosed with autism, she has become a major player in autism advocacy and research.

In the beginning, Iversen spent many long nights online, searching for information about her son's condition. "I'd spend hours and hours working my way through these complicated neuroscience websites," she remembers. "No one knew what caused it [autism]. The only thing they all agreed on was that there was no treatment. There was no cure. There was little ongoing research. And the few active researchers weren't even telling other researchers what they'd found."

At first, Iversen and her husband, Jon Shestack, tried to persuade autism researchers to share their DNA samples with other scientists. But the researchers weren't about to turn their hard-earned results over to their competitors. "They had their own agenda. And it didn't always lead to getting new treatments out to the people who needed them ASAP," Iversen says. After running into a brick wall again and again, Iversen and her husband decided to establish the Autism Genetic Resource Exchange (AGRE), a databank of tissue samples collected from families with two or more autistic children. It is now the world's largest autism gene bank, supplying tissue samples and biomedical records from 500 families to more than 40 medical research teams. Researchers who use the gene bank must agree to share their results openly with other scientists. We felt that this would be a great way to speed up the progress of current autism research and attract prospective researchers to the field," Iversen says. "We hope that something good for Dov will come out of it."

"Portia's gene-bank idea has been extraordinarily effective in stimulating new work in this badly neglected area," says Daniel Geschwind, M.D., Ph.D., director of UCLA's neurogenetics program. "The work her group has done has motivated dozens of new researchers to study the genetics of autism. As recently as three years ago, no researchers were addressing this badly neglected area. Today, there are dozens of research teams. It should be just a matter of time until we're able to identify the key genes responsible—and to come up with effective treatments."

On February 18, 2007, The Autism Genome Project Consortium (AGP), of which AGRE is a primary partner, published its report on a link between autism spectrum disorders and a site on chromosome 11.[13] This project involved researchers from 19 countries examining genes from 1,168 families. The work that Portia Iversen initiated years ago has evolved into a significant research collaboration hailed by NIH Director, Elias Zerhouni, MD: "This is the most ambitious effort yet to find the location of genes that may confer vulnerability to autism. The AGP is revealing clues that will likely influence the direction of autism research for years to come."[14]

Parent Expertise on PXE

In 1994, Sharon Terry noticed that her seven-year-old daughter, Elizabeth, had a bumpy rash on both sides of her neck. When she asked her pediatrician about it, he insisted that it was nothing to worry about. Terry decided to get a second opinion. A dermatologist examined Liz briefly and told Terry that her daughter had *pseudoxanthoma elasticum* (PXE), a rare genetic disorder that affects connective tissues throughout the body. He then glanced down at her son Ian, age five, who'd accompanied his mother and sister to the clinic. "Ah, he has it too," the doctor said.

Terry set out to photocopy and read every article about PXE in the medical library at the University of Massachusetts at Worcester. "Popular medical resources such as the Merck Manual described the condition in dire terms, including the possibility that our kids would die at age thirty," Terry recalls. "What most jarred us was the realization that the research-medical system was not a well-oiled machine. We began to understand that we could not expect accurate information or a course of treatment."[15,16,17] Then she and her husband contacted all major PXE researchers to request information about their work. But the more she learned, the more alarmed she became.

"PXE could cause heart disease, GI bleeding, and severe vision loss," she remembers. "Their arteries could harden. They could bleed internally at any time. They could go blind. And they would almost certainly die prematurely of this disorder. The worst part was that our doctors seemed to know almost nothing about it. "In the beginning, my husband, Patrick, and I didn't know a gene from a hubcap,'" she says. "But we didn't let that stop us. We became obsessed. The little playroom behind our kitchen became our office. We began reading thick medical textbooks and obscure genetics journals and sticking genetics charts up on our walls. We learned as we went along."[18]

"PXE was so rare that there was no central registry. And virtually no research was being done." So, Terry and her husband set out to get medical researchers to pay more attention to this understudied ailment. "But Pat and I had a lot to learn about the politics of medical research," she recalls. "You can't just walk into some researcher's office and demand that they look into PXE."

Like Jannine Cody, the Terrys decided to set up a registry and tissue bank to support genetic research. Within the next few months, the Terrys visited 24 countries, collecting more than a thousand tissue samples from PXE patients. Their playroom soon became the headquarters of PXE International, which now boasts 35 offices worldwide. And they began working side-by-

side with a team of medical researchers, advising on symptoms and consulting on research strategies.

“Finally, in 1999, our efforts paid off,” Terry recalls. “The University of Hawaii researchers we were collaborating with, and two other research groups we’d been supporting, identified the gene that causes PXE.” In August 2004, Sharon Terry did something no parent had ever done: she received a patent on the gene that caused her children’s disorder.[19] “In other cases, when patients have helped researchers like this, the researchers patented the gene and controlled all rights to it,” Terry explains. “But that can impede further research and keep new advances from becoming available and affordable. We wanted to make sure that any genetic tests that result will be inexpensive and widely available. That’s why we applied for the patent.”

“My work with PXE International has taught me that consumers can be central to the research endeavor,” Terry concludes. “We can be a catalyzing force for translating research into the services we desperately need, such as treatments, technologies to alleviate suffering, and clinical methods of dealing with the conditions. I now serve as president of the Genetic Alliance.[20] This role enables me to work with other groups so that we leverage each other’s capacities to make a difference for our loved ones.”[21]

e-Patients’ Role in Future Medical Research

Before the Internet, researchers called the shots, research trials were structured to meet their needs, and the delay between discovery and dissemination was considered an unavoidable part of the process. But as e-patients become more capable researchers—and research partners—a wider range of approaches to medical research are becoming realistic.

Research-oriented online support groups can design and conduct their own studies, collect and analyze their own data, and publish their own results. Online groups can provide researchers with access to perfectly targeted study populations at little or no cost. But whatever role they play, once they begin to wield real power in the research enterprise, patient groups will want to have a voice in shaping the future directions and strategies of medical research studies.

The Life Raft Group’s entire cash budget for its first year of operation was $225.00, yet this group has accomplished extraordinary results. e-Patients are motivated to find answers and they are willing to donate massive amounts of time – something most researchers don’t have. And while such e-patient initiatives may encounter some resistance, in the end, it seems likely

that the financially strapped medical research establishment will decide that such e-patient initiatives represent an offer contemporary healthcare can't afford to refuse.

"It's so exciting to be involved in this new initiative, to try to figure out how online groups can collect and disseminate good information," Scherzer says. "We find it extremely encouraging that clinical researchers at the leading drug companies and treatment centers are now beginning to take us seriously. The fact that we've been able to collect, interpret, and publish medically-valid studies makes all the difference in the world."

"We now have great relationships with all PXE medical researchers," says Sharon Terry. "And while we've learned a lot about biology, chemistry, and molecular genetics, we don't involve ourselves in the competition among professional researchers. We support everyone. This makes us a very powerful ally." A dozen other self-help organizations have asked Terry's group to teach them how to initiate leading-edge research for their own respective conditions.

PAGER's McGraw and Pulsifer-Anderson advised: "There is an important lesson for medical professionals in all this: Families were the ones who first noticed a pattern of inherited reflux, and families were the ones who sought out researchers who were willing to explore this 'wild idea.' We strongly encourage other patient groups to be assertive if they see patterns that do not fit the current medical theories. Medical theories change and you can jump-start that change through hard work, persistence, and a little luck."

"As these examples show, patient-driven research will become more and more important," says ACOR's Frydman. "As more patient groups begin gathering and publishing their own medical data, it will change the relationships between researchers and patients in dramatic ways. Researchers will increasingly depend on patients, not the other way around. These groups have been phenomenally successful in recruiting new patients for needed clinical trials. And I can assure you that e-patients' potential role in medical research is not lost on the drug companies."

In the chapters that follow, we suggest that initiating and conducting medical research is not the only major healthcare contribution our new generation of e-patients is now ready, willing, and able to make—if we will only let them. ■

Author's Note—The original, and still in many ways, definitive article on e-patients as researchers is "The Citizen Scientists," by Reviewer Sara Solovitch, which originally appeared in *Wired* magazine in

Sep. 2001 (issue 9.09), <http://www.wired.com/wired/archive/9.09/disease.html> (Aug. 6, 2004). Solovitch won the 2002 American Society of Journalists and Authors Outstanding Article of the Year Award for Reporting on a Significant Topic for this article. It is also available from Solovitch's website at <http://www.sarasolo.com/wm1.html> (Accessed on Aug. 6, 2004). This chapter has drawn heavily on Solovitch's pioneering reporting.

Parts of this chapter were adapted from Tom Ferguson, "e-Patients as Medical Researchers," *The Ferguson Report*, no. 9, Sep. 2002. <http://www.fergusonreport.com/articles/fr00903.htm> (Accessed on Aug. 6, 2004).

Amy Dockser Marcus, "Medical Student Takes On A Rare Disease–His Own," *The Wall Street Journal Online*, Apr. 1, 2004. <http://online.wsj.com/article/0,,SB108077194020270691-email,00.html> (Accessed on Aug. 6, 2004).

[2] Bounce for Life, <www.bounceforlife.org> (Accessed on May 28, 2004). Not active as of Feb. 18, 2007.

[3] Marcus, op. cit.

[4] Ibid.

[5] Bounce for Life, <www.bounceforlife.org/bfl/andy.html> (Accessed on Aug. 10, 2004).

[6] Marcus, op.cit.

[7] Tom Ferguson, "e-Patients as Medical Researchers," *The Ferguson Report*, 9 (Sep. 2002). <http://www.fergusonreport.com/articles/fr00903.htm> (Accessed on Aug. 6, 2004).

[8] Ferguson, op. cit.

[9] The Leiomyosarcoma Online Support Group, <http://listserv.acor.org/archives/l-m-sarcoma.html> (Accessed on Aug. 6, 2004).

[10] Life Raft Group Newsletter, <http://www.liferaftgroup.org> (Accessed on Aug. 6, 2004).

[11] http://www.liferaftgroup.org/

[12] Fen Ze Hu, et al., "Mapping of a Gene for Severe Pediatric Gastroesophageal Reflux to Chromosome 13q14," *Journal of the American Medical Association* 284 (2000): 325-34.

[13] Peter Szatmari et al., "Mapping Autism Risk Loci Using Genetic Linkage and Chromosomal Rearrangements," *Nature Genetics*, 39 (2007). Published Online, Feb. 18, 2007. doi:10.1038/ng1985.

[14] NIMH Press Release, 18-Feb-2007.

[15] Sharon F. Terry, "Learning Genetics," *Health Affairs*, 22 (2003): 166-171. <http://content.healthaffairs.org/cgi/content/full/22/5/166> (Accessed on Sept 2, 2004)

[16] The Genetic Alliance (http://www.geneticalliance.org) is the world's largest coalition of genetic advocacy organizations, including more than 600 advocacy groups, community groups, health professionals, researchers, hospitals and clinics. Founded in 1986, it is a 501(c)(3) organization that provides technical resources and support to lay advocacy organizations and serves as a collective voice in matters of policy, research, education and advocacy. Inquiries are welcome at 202-966-5557.

[17] Elizabeth Pennisi, "Patients Help Track Down Disease Gene," *Science* 288 (2000): 1565-7. <http://www.sciencemag.org/cgi/content/short/288/5471/1565>(Accessed on Sept 2, 2004)

[18] Terry, op. cit. Also see: Sharon F. Terry et al., "Advocacy Groups as Research Organizations: the PXE International Example," *Nature Reviews. Genetics* 8 (2007): 157-64.

[19] Eliot Marshall, "Patient Advocate Named Co-Inventor On Patent for the PXE Disease Gene," *Science* 305 (2004): 1226. <http://www.sciencemag.org/cgi/content/full/305/5688/1226a> (Accessed on Sept 2, 2004)

[20] <http://www.geneticalliance.org/ws_display.asp?filter=bio.terry> (Accessed on Mar 19, 2007)

[21] Terry, *Health Affairs*, 2003.

Medical knowledge is a social process:
The conversations that occur around
artifactual data are always more
important than the data themselves.
—John Lester

CHAPTER SIX

Learning from e-Patients

e-Patients can take many different routes to empowerment. Some join an online support group or read about their condition through extensive online searches. Others consult resources such as Medline to learn about the latest research and treatments for their health concern. But some e-patients discover that current online resources don't meet their needs. Often working in partnership with innovative health professionals, they strike out on their own looking for a resource, technology or modality of care that meets their particular needs.

Three examples of this type of innovation are discussed in this chapter. We consider the implication of Braintalk, the online patient information and support community for neurological concerns described in Chapter 4. The second example looks at how e-patients drove the creation of online mental health services, or e-therapy. The third example describes the growing practice of a concierge practice model of medicine, where e-patients become true partners in their health care choices with doctors who limit their practice to such patients. These examples show how 21st century medicine can learn new approaches from e-patients.

What Happens When e-Patients Take the Lead?

The resourcefulness and creativity of BrainTalk's members continue to astound its developers. After establishing the online resource, Lester and his colleagues noticed that BrainTalk patients had created a new chat room called Club Avonex. "It was developed by a group of Multiple Sclerosis (MS) patients who were giving themselves home injections of a MS drug called Avonex," Lester recalls. "Most found the self-injection process extremely stressful. So, even though they lived in many different time zones, they all logged on to the Club Avonex chat room at the same time so they could inject themselves simultaneously. This made it possible for participants to offer each other real-time guidance and support—before, during, and after the injection."[1]

But as the community has grown, it has also faced its own share of challenges. "A recent hardware malfunction taught us a number of lessons about resources like Braintalk," says

Hoch. “First, if there were ever any doubts about how important the resource is to its users, shutting it down unexpectedly quickly removes those doubts. E-mails from concerned users about the fate of Braintalk started within minutes of it going offline. Extensive user networks rapidly involved, began talking to each other and pooling resources and contacts, to learn more about what was going on and how they could help. Frankly, such hardware failures are inevitable, but this one taught us how critical it is to immediately create a status page informing visitors about the fate of the site and repairs.”

“The second lesson is that a resource this important to a large community cannot be based on the efforts of a single individual,” Hoch continued. “Unfortunately, in many cases very large communities are indeed dependent on an individual or small group of individuals. I suspect we are in a transitional time for such communities and many of them are moving to commercially hosted solutions where backups, redundancy, and hardware resources are easy to arrange. But there are trade-offs in cost, privacy, and direct control. In our case, the technical management of Braintalk rests completely in one person’s domain. It is a labor of love, and the community appears to want and encourage this kind of arrangement. On the other hand, those same community members can become pretty irate when there is a hardware failure and apparent breach of the implicit trust between them and their community administrator. Clearly, given how critical the community is to its users, we must build backup systems and ways to recover rapidly from hardware failure.”

Having learned about the value and dynamics of online groups through the team’s e-patient research, Hoch, a neurologist specializing in epilepsy, now routinely invites his patients to participate in a private online support community for epilepsy. And while the patients own and run the online community, Hoch often takes an active part in their discussions. As group members get to know one another and become familiar with each group member’s unique neurological conditions, he’s working with them to explore more sophisticated forms of co-care—new ways in which clinicians and patients can collaborate in providing more convenient and sophisticated medical care for group members.

Professionals Offering Online Mental Health Help

By the time she finally went looking for an Internet therapist, Martha Ainsworth, a freelance Web developer and communications consultant, had been leading online support groups for more than a decade. So she knew what valuable help and guidance they can provide.[2] She also knew that such help is not always enough. She had seen a large number of e-group

participants admit online to despair and suicidal feelings that they might be embarrassed to acknowledge face-to-face. She wished that all those who found it easier to reveal their innermost thoughts online could have online access to a professional therapist.

When Ainsworth found herself facing her own dark night of the soul, she looked for just such an online therapist.[3] After several hours of searching, she came upon a Web page that began, "Welcome to the Mental Health Cyber-clinic."

> **The therapist had written several pages describing the e-therapy helping process. His tone was informal, but professional. I could see that he knew how to communicate in writing; his gentle compassion shone through his words. Best of all, he spoke of forming a relationship… He was winning my trust before he ever knew of my existence…**
>
> **Our e-mail relationship developed gradually. He set out the ground rules and offered a few gently probing questions to help me start telling my story. I felt an enormous sense of relief. He was caring and willing to help. The anonymity of cyberspace made me feel free to tell him everything. And the ability to direct my own therapy online made me feel tremendously empowered: I didn't need to wait for an appointment—I could proceed according to my own time schedule… Little by little, as my trust deepened, I shared the deeper issues I'd never been able to discuss with anyone. He responded with caring and warmth. Our relationship became a well of caring that to this day has never run dry.**
>
> **Nine months later, we met in person. But sitting across from him in his therapy office just wasn't the same. He was just as caring, just as warm, and just as insightful. It was I that was different. Because we were face to face, there were some things I simply could not say. I hurried home from that first visit, eager to share all my unspoken thoughts and feelings—by e-mail.**[4]

Ainsworth created a website (http://www.metanoia.org) to help other e-patients find qualified e-therapists and work with them effectively. And even though she stopped updating the site in 2002 (when she became community producer of Beliefnet.com, the Internet's largest multi-faith spirituality website), the metanoia.org site is still one of the best resources on the subject. Articles available there include:

- **Talk to a Therapist Online**
- **How to Choose a Competent Counselor**
- **If you are suicidal, read this first**

Reaching Those Who Wouldn't Ordinarily Consult with a Professional

Although online mutual self-help support groups like BrainTalk have been around since the 1980s,[5] online counseling, or "e-therapy," is an invention of the Internet boom years in the mid-1990s. Driven by the increasing popularity of the Internet, people with mental health

concerns started seeking out mental health professionals for advice, consultations, and yes, even psychotherapy online.

"e-Therapy is not just psychotherapy transplanted to an online medium," says White Paper advisor John Grohol, PhD. "It is something completely new. And for some patients, it appears to be not only more convenient, but also more effective, than conventional psychotherapy." Psychologist Grohol, who founded the pioneering mental health website Psych Central (http://www.psychcentral.com) and wrote *The Insider's Guide to Mental Health Resources Online*, coined the term 'e-therapy' in 1993.

"From a public health point of view, e-therapy offers a way to reach millions of patients with psychological and mental health conditions who would be unlikely to seek face-to-face therapy," Grohol says. "One in five Americans has a diagnosable psychological problem, yet nearly two-thirds of those affected never seek professional help.[6] Some feel they can't afford it. Some find it too embarrassing to discuss sensitive issues face to face. Others come from cultures in which seeking professional help for mental health problems is considered a sign or weakness or instability. But a new generation of mental health professionals is now learning to use the Internet to extend a helping hand to those who would never darken a therapist's door. And as a result, thousands of e-patients are now finding online mental health services more accessible than ever before."

"Look, e-therapy will never replace traditional face-to-face therapy," says Grohol. "But it can provide people with an introduction to the benefits of the therapeutic relationship and process. It can be a stepping stone to regular therapy for those who feel overwhelmed with the idea of talking to a psychotherapist face-to-face."

The International Society for Mental Health Online, co-founded by Grohol, Ainsworth, and others in 1997, published a white paper in 2000 entitled "Suggested Principles for the Online Provision of Mental Health Services," a set of guidelines for e-therapy (available on their website at www.ismho.org). More information on e-therapy can be found at metanoia.org, and in Grohol's series of essays on "Best Practices in e-Therapy" (http://www.psychcentral.com/best/).

Learning what Patients Really Want from Clinicians

For many years, Harvard's Rushika Fernandopulle has been a tireless champion of healthcare reform. Until recently, he led the Harvard Interfaculty Program for Health Systems Improvement, a group of senior Harvard healthcare experts seeking innovative solutions to the

largest, most difficult problems facing healthcare. He and colleague Susan Sered wrote a book in 2005 entitled, *Uninsured: Life and Death in the Land of Opportunity,*[7] which is a scathing exposé of the difficulties and hardships faced by Americans without health insurance. So when Dr. Fernandopulle recently announced that he was leaving his job at Harvard to join two other Boston internists at a retainer-based medical practice in a nearby suburb, some of his medical colleagues didn't know quite what to think.

Retainer-based medicine is a new type of medical practice in which physicians provide a limited number of patients with a specific set of services in exchange for an annual fee. Its detractors sometimes call it "concierge medicine." Critics charge that such practice models are simply a way for greedy doctors to get more pay for less work by offering their services to the rich while ignoring the poor.

Dr. Fernandopulle admits that in a few cases, this may be true. "There are a few retainer-based practices in which physicians limit their practices to 50 wealthy patients that pay $20,000 apiece for the privilege—and spend most of their time playing golf," he says. "But most clinicians who have established retainer-based practices are simply seeking relief from the soul-numbing responsibilities of caring for too many sick patients in too little time. It's so frustrating to be forced to practice medicine in a situation in which people expect so much of us and we have so little time and attention to give them."

But for some reform-minded clinicians, like Fernandopulle and his colleagues, the retainer-based practice model is the most promising new opportunity for clinical innovation in their lifetimes.

"A completely new model of clinical care is needed," Fernandopulle suggests. "Even at a world-class medical center like Boston, it's gotten so bad that most of us take the defects for granted. Patients expect long delays in getting doctor's appointments. They expect to have to wait long hours in our waiting rooms. They expect rushed, time-pressured visits from overworked, distracted clinicians. They expect to be treated rudely by clinic staffers. They expect that it will be difficult or impossible to contact their clinician in a medical emergency.

"In the traditional clinical model, the doctor essentially works for the insurance company, not the patient," Fernandopulle says. "And clinicians can only do what the insurance companies will pay for. If our patients say, 'We want to communicate with you by e-mail,' we doctors have to say, 'Forget it! Your insurance won't pay for that.' If they tell us they want to learn more about their illnesses, it's just too bad. We have 30 more patients waiting. If some innovative

clinicians say, 'We want to make electronic medical records available to patients on the Net,' the insurance companies say, 'We won't pay for that either.'"

"The retainer-based model gives us a chance to say, 'Okay... We're really going to change things. We're going to find some exciting new ways for clinicians and patients to work together. We're going to work with our patients to develop new models of empowerment-based, patient-driven care. And if we succeed, the new practice patterns we create could provide a workable business model for widespread healthcare reform."

At the core of Fernandopulle's vision is a radically different clinician-patient partnership in which patients take on a higher degree of responsibility—for their own care and for running the clinic—while clinicians make themselves available to patients in more convenient and supportive ways. This underlying philosophy can be seen in the invitation he and his colleagues offered to prospective clients on their website when it was first launched:[8]

What if...

- **You could get questions answered, by phone or by e-mail, without necessarily having to go to the doctor's office?**
- **You could always see your doctor right away, whenever you needed to, for as long as you needed to?**
- **You never had to wait in a doctor's waiting room again?**
- **You never had to go to the emergency room again, except for a real emergency?**
- **Your whole experience of dealing with your clinical team was so much better than it used to be that you actually looked forward to the interaction?**

Fernandopulle and his colleague Pranav Kothari developed their new practice model during a two-year research project, sponsored by the Harvard Interfaculty Program for Health Systems Improvement, in which they traveled around the country visiting the most innovative clinics they could find. In the end, they adopted the membership-based model because the insurance companies refused to pay for a number of things they considered vital:

- **Being available to patients by e-mail and cell phone on a 24/7 basis.**
- **Giving patients access to their medical records via the Internet.**
- **Offering newly diagnosed patients crash courses on their disorders.**
- **Training and supporting patients to practice self-managed care.**
- **Checking clinical practices against recommended medical guidelines.**

- **Asking patients to critique their services and to suggest better ways to meet their needs.**
- **Involving patients in the governance of our clinic.**
- **Providing online support communities for patients.**

"In the traditional clinical practice, the insurance company comes first, the provider institution comes second, physicians come third, nonphysician staffers come fourth, and the patient comes last," Fernandopulle says. "How else can you explain the difficult time patients have getting an appointment or getting through to their doctor in an emergency? How else can you explain the excessive waiting times, the inadequate length of clinical visits, clinicians who see 40 or 50 patients in a day, and the many petty humiliations the average patient must endure?

"Why do we treat without teaching, prescribe without explaining, issue 'doctor's orders' without discussing the full range of possible alternatives, and provide care without routinely providing patients with copies of their records? Why do we make patients come to the clinic for information we could give them by phone or e-mail? Why don't we allow patients to provide their own care—or to provide care for others?

"The traditional practice sees patients as the passive recipients of professional services. But our practice sees patients as the central players in the whole healthcare process. They initiate most interactions by e-mail or phone and we help them deal with their medical concerns at their convenience. We'll see you right away, whenever you need us. If you find yourself facing a new medical condition, we'll introduce you to expert patients with the same disorder. We'll invite you to join an online support group where doctors and patients work together to provide care for everyone. And if there's a medical procedure you'll be needing regularly, we'll teach you—or a family member—how to do it.

"Our underlying strategy is to approach the problem from both ends," Fernandopulle says. "We'll do all we can to help members stay healthy. If they become ill, we'll help them become expert patients. And we'll provide them with the training and support they need to provide most of their own medical care themselves. This will free us up to spend more time with the patients who really do need to see us."

Fernandopulle and Kothari aren't practicing in this new way just to reduce healthcare costs, he insists. "We're trying to find a more sustainable and sensible way to practice medicine in the age of e-patients. But we suspect that in the long run, this new empowerment-based, patient-driven model will be less expensive than the old doctor-centered approach. We're hoping to

inspire insurance companies to pay for aspects of this new model that they currently refuse to support." ■

---

[1] Tom Ferguson, "Medical Knowledge as a Social Process: An Interview with John Lester," *The Ferguson Report*, no. 9, Sep. 2002. <http://www.fergusonreport.com/articles/fr00902.htm> (Dec. 2, 2006).

[2] Martha Ainsworth, "An e-Patient's Story." *Metanoia*, 2001, <http://www.metanoia.org/martha/writing/epatient.htm> (Dec. 2, 2006).

[3] Ainsworth, 2001.

[4] John Grohol, *The Insider's Guide to Mental Health Resources Online*, (New York: Guilford Press, 2003).

[5] John Grohol, "Online Counseling: A Historical Perspective," in *Online Counseling: A Handbook for Mental Health Professionals*, Eds., Ron Kraus, Jason S. Zack and George Stricker, (New York: Elsevier, 2004).

[6] *Mental Health: A Report of the Surgeon General,* Washington, D.C.: Department of Health and Human Services, 1999. <http://www.surgeongeneral.gov/library/mentalhealth/home.html> (Dec. 2, 2006).

[7] Susan Sered and Rushika Fernandopulle, *Uninsured: Life and Death in the Land of Opportunity*, (Berkeley: University of California Press, 2005).

[8] Renaissance Health, "What If…", 2004, <www.renhealth.net> (Dec. 2, 2006).

We must redefine the patient's role
to emphasize autonomy, emancipation
and self-reliance instead of passivity
and dependence.
—Angela Coulter

CHAPTER SEVEN

The Autonomous Patient and the Reconfiguration of Medical Knowledge

Lay citizens have always been an important, though often unacknowledged, component of the healthcare workforce. When 518 individuals of all ages were asked to keep a four-week health diary in a 1995 study, only 22 percent of the 500 illness episodes they reported were ever discussed with a clinician.[1] They were managed as follows:

**No treatment needed**	**24 percent**
**Self-care only**	**54 percent**
**Self-care and professional care**	**17 percent**
**Professional care only**	**5 percent**

Other studies have reached the same conclusion: three out of four illness episodes are managed without professional help.[2] And nineteen out of twenty illness episodes are either partly or completely self-managed.

Yet patients of earlier generations could do only so much. While they frequently took care of minor illnesses on their own, when faced with a more serious or troubling medical concern, they had only one option: to put themselves in the hands of a medical professional. The clinician they chose would take a history, perform a physical exam, order any necessary tests, and would then explain what the problem was and what the patient must do about it. The clinician might pause on the way out, one hand on the exam room door, to ask, "Any questions?"

Patients of earlier generations often treated their doctors as if the doctors knew everything but would be upset if the patients dared ask questions. They would never question the advice or treatment that was suggested to them. Armed with information gathered from the Internet, today's e-patients have an opportunity to acquire a more sophisticated and realistic understanding of their illness and thus are much better equipped to question or interact with their clinicians. Today's e-patients can also assess a clinician's abilities and limitations more accurately. They know, for instance, that clinicians cannot always be up-to-date on the latest

treatments for all diseases. They also know that, if necessary, they can get a second opinion from an online patient-helper, an online support group, or a virtual medical professional. By contrast, patients of earlier generations had little choice but to accept whatever advice or care their clinician offered.

Today's e-patients have more choices and are more confident about exercising them. When offered sub-optimal treatments, they may reject them and go elsewhere. Or they may attempt to educate their clinicians about the current state-of-the-art treatments and try to convince them to upgrade their services. In some cases, an e-patient may discover a top specialist for her specific condition, and make periodic trips to consult with him to make crucial treatment decisions, while using her local provider for medical support.

e-Patients have thus become important healthcare resources in their own right, and they have done so despite their lack of formal medical education, training, or support. Legal and cultural barriers make it difficult for them to obtain the drugs, tools, and tests they need. All too frequently, their clinicians' paternalistic or frankly antagonistic attitudes toward e-patient behaviors pose another obstacle. In spite of their increasing abilities and their desire to serve as a healthcare resource, a complex array of medical, cultural, and legal limitations remain in place and serve to discourage today's e-patients from autonomy and independence from their medical professionals.

The Changing Medical Paradigm

One of the earliest and most perceptive professional observers of the e-patient phenomena was Michael Hardey, a medical sociologist at the University of Southampton.[3] Hardey was one of the first health professionals to describe the "rich network of personal home pages developed by patients," which now serve as "a powerful new global Internet referral network."[4] Hardey was also the first professional commentator to identify two revolutionary aspects of the e-patient's emerging role:

- **In the judgment of those who really count—e-patients themselves—the most useful online information and guidance available on a given medical topic often comes from online patients (e.g., patient webmasters and e-patient groups) rather than medical professionals.**

- **It is these e-patients themselves—and not their clinicians—who choose the online resources they prefer. And it is they who decide when and how to use them.**

Hardey goes on to describe the many ways in which e-patients have become significant providers of health information, advice, and care.[5] And he identifies the pivotal underlying trend behind the contemporary e-patient revolution: A fundamental *reconfiguration of medical knowledge and expertise*, brought about by the Internet, is transforming the roles of patients and clinicians alike. Many e-patients, under certain circumstances, may have more knowledge about, or a more sophisticated understanding of, some aspects of their disease than their clinicians:[6]

> **• [My physician] was not aware of Keppra [a recently introduced anti-epileptic drug] until I told him I had heard of it being very helpful while reading online forums. He decided to read up on it and later prescribed it for me.**
>
> **• Doctors do not have time to remain current… about every disorder, so I give [my doctor] the cutting edge information on [Asperger Syndrome and migraines] so that I can benefit from new thoughts and therapies… [My doctor] is glad I do that.**
>
> **• [My physician] was not aware of magnetoencephalography (MEG) being used in cases of epilepsy surgery. I provided the information, and he did contact the researcher and arrange for my child to go to Utah for testing. [This] provided… information no other test had and my son was approved for brain surgery.**
>
> **• I had to ask my doctor if it was possible that I have hemochromatosis. He nearly fell off his chair and said, "YES! I will order tests." My son found the information on the Internet… The doctor called two days later to say, "Yes, you were right…."**

Yet many other e-patients report that their clinicians have been less than enthusiastic about their use of online health resources, their new expectation of increased autonomy, and their desire for collaborative partnerships with their providers.

Who's In Charge Here, Anyway?

Long ago, in the U.S. healthcare system in the middle of the 20th century, medical information was the exclusive intellectual property of clinicians. Mid-century medical professionals thought of this hegemony over medical knowledge as their God-given right and considered it an inherent characteristic of healthcare. But it now appears that this was merely a cultural artifact of the time. Its continuation depended on a number of prevailing conditions:

> **• In-depth medical knowledge would remain available only to professionals and would not be shared with patients.**

- **Medical tests and tools and many medications would continue to be available only to or through professionals.**
- **Patients would not share their clinical knowledge and their medical experiences with each other.**

The medical information of the day was available from only a few tightly controlled sources: professional medical training, medical textbooks and journals, medical libraries, professional meetings, and in-depth consultations with knowledgeable clinicians. And with a few notable exceptions (e.g., the Library of Congress) none of these resources was available to patients.

As a medical student in the mid-1970s, I made copies of key medical journal articles for my patients and encouraged them to learn all they could about their diseases. And on several occasions I invited a few of the best and brightest to accompany me to the Yale Medical School library so that we could research their conditions together. But the medical librarians refused to admit them. When I asked why, I was told that the library's physician advisory board felt that "any patient seeking library access was probably just gathering information for a malpractice suit." It was a chilling reminder of the way I had, while doing civil rights work in the South a few years earlier, been refused admittance to several Florida restaurants because my companions were black.

The Configuration of Medical Knowledge in 20th Century Medicine

With the notable exception of Dr. Benjamin Spock (who was savagely attacked by his medical colleagues when he opened his medical toolbox for lay people, telling the parents who read his mid-century classic, *Baby and Child Care*, "You know more than you think you know"), few mid-century clinicians shared their in-depth knowledge with patients. While they might occasionally provide a "patient handout" containing a list of instructions for care at home, they rarely shared their medical journal articles or the thought process that led to a particular diagnoses or choice of treatments with their patients. And they were almost never willing to share their ignorance or their uncertainty.

In the late 19th century, Oliver Wendell Holmes assured a group of medical students that, "Your patient has no more right to all the truth you know, than he has to all the medicine in your saddle bags . . . he should get only as much as is good for him." [7] My instructors occasionally cited this old chestnut when I was a medical student on the wards of Yale-New Haven Hospital in the mid-70s. And the same distinguished medical educators repeatedly reminded me that I must strive to convince each patient I saw that my colleagues and I knew everything we needed to know about his or her condition and its treatments—even when I felt

this was not the case. Should I fail to do so, I was warned, my patients might “lose confidence” in me.

Many clinicians still see little need to inform or involve the patient. They may focus on treating the disease, rather than the person who has it. One of my distinguished Yale Medical School professors told us that it gave him great comfort to know that in hospitals and clinics all over the world, when confronted with a particular diagnosis, “his boys” would prescribe the treatment he always endorsed. Even though half our class was comprised of women, we all got the message: the individuality, knowledge, or capability of the patient was not essential to the physicians’ treatment decisions. Prescriptions may be written and surgeries performed with little or no consideration as to how the “gold standard” of the day for that condition might apply to this particular individual. Many doctors who had a similar education have carried this attitude into their practice. Patients who fail to follow doctor’s orders may be labeled “uncooperative” or “noncompliant” patients. Doctors frequently feel frustrated if their patients aren’t adequately convinced to follow doctor’s orders; it is, after all, for the patient’s own good.

The Pitfalls of Paternalism

Health professionals historically tended to assume that only medically licensed clinicians could deal responsibly with medical matters, and that only health professionals could provide quality medical information. Patients were considered incapable of understanding and taking charge of medical matters. Given these assumptions, the 20th century medical model was paternalistic in nature. It forced patients into a passive mode, making them bystanders in matters of their own care.

“The model for most health care is still ‘doctor knows best,’ with patient input low, and patient preference, for the most part, disregarded,” says John E. Wennberg, director of the Center for the Evaluative Clinical Sciences, Dartmouth Medical School. “The problem with this template, apart from its essentially authoritarian nature,” Wennberg adds, “is that the doctor may, indeed, *not* know best.”[8]

As an example, Wennberg points to radical mastectomy: “It took 60 years to establish the fact that, for many patients, a simple mastectomy is just as good as a radical one. Further studies have shown that breast-sparing surgery, when combined with radiation, has about the same impact on life expectancy as simple mastectomy. The treatments, however, can have very different impacts on the quality of life. The choice between the two approaches ought to depend on which treatment the individual patient wants, and how she weighs the risks and

benefits. But in many parts of the country, mastectomy is still often the only option women are given, due to physician preference for the techniques they learned 20 years ago in medical school."[9]

Medical professionals need to acknowledge the many ways in which the paternalistic biases of our medical training have influenced our thinking. Angela Coulter, chief executive of the Picker Institute Europe, has suggested that a necessary first step in influencing the health care system to respond appropriately to our first generation of e-patients will involve coming to terms with the "…the pervasive paternalistic tendencies that are still the defining characteristic of much contemporary medical care."[10]

In her visionary 2002 manifesto, *The Autonomous Patient: Ending Paternalism in Medical Care,* and in her other writings, Coulter challenges clinicians and policy-makers to radically reassess a wide range of current clinical practices, with an eye to identifying and reforming these paternalistic tendencies. She identifies several dysfunctional aspects of paternalistic healthcare:

- **Paternalistic clinicians seriously underestimate the degree to which patients feel intimidated by the setting of the clinical encounter.**
- **Many patients fear that their clinicians will be offended if patients assert their needs or share their agenda.**
- **Many paternalistic clinicians are unaware of the constraints that may make it difficult or impossible for patients to ask questions. As a consequence, patients may leave the physician's office with many questions unanswered.**
- **Many medical professionals believe that patients are fragile and should be protected from the truth. Such clinicians are likely to conclude that patients cannot cope with bad medical news and must be kept ignorant of poor prognoses or other medical uncertainties.**
- **Paternalistic clinicians frequently make their patients feel inept and incompetent, sapping their confidence, diminishing their sense of control—yet simultaneously encouraging them to rely on their healthcare professionals to solve their problems—even when this is unrealistic.**
- **In paternalistic practices, so-called "informed consent" is often little more than a pretense, a bureaucratic obstacle to be overcome as quickly as possible.**
- **Paternalistic clinicians often refuse to accept ideas or suggestions from patients. Patients typically describe such clinicians as authoritarian, arrogant, unsympathetic, and unsupportive.**

The e-Patient-Resistant Clinician

We have used this term to identify those health professionals who refuse to acknowledge their

patients' competence or to accept their influence. We were unwilling to call them arrogant, as a number of e-patients suggested; for in many cases these hard-working professionals are only doing what they were taught. But, while some may be highly admirable practitioners in the context of the traditional physician-patient relationship, their behavior toward their clients is sometimes dismissive, or even abusive.

e-Patient-resistant clinicians may, in their most flagrant manifestations, use their medical authority to discourage patient questions, warn patients about becoming "overly educated," or intimidate patients into accepting their opinions—sometimes in the face of overwhelming contrary evidence.

Moving Beyond Medical Paternalism

Coulter suggests that the pervasive paternalism that has pervaded all modern healthcare systems for most of the last century can no longer be tolerated: "Given our current crisis, we must make it clear, once and for all, that such paternalistic hallmarks as long waiting times, lack of information, uncommunicative staff, and failure to seek patients' views and take account of their preferences can no longer be tolerated.

"We must redefine the patient's role to emphasize autonomy, emancipation, and self-reliance instead of passivity and dependence," Coulter urges. "Patients must be treated as co-producers of their own health and [as] case-managers when they are ill**….** Rather than treating patients as passive dependents who can do nothing for themselves and whose views can be discounted, we must recognize patients' ability to contribute to their care, to critique professional services, and to choose the most appropriate treatments."

Under the 21st century patient-driven model, Coulter proposes that patients be treated as "…responsible adults, capable of gathering and assimilating information and of learning the skills needed to provide much of their own medical care." The clinicians' roles in 21st century healthcare will increasingly be to support their patients' own initiatives, to encourage patients to practice self-managed care, to help patients acquire the information, skills, tools, and support they need, and to serve as advisors along the way. When patients are unable or unwilling to deal with a medical problem on their own, a clinician should be ready to assist them in solving it.

The "e-Patient-Receptive" Clinician

When patients are given the opportunity to collaborate with a non-paternalistic clinician, asking questions in their own way and communicating via e-mail when needed, actual consultation times typically do not increase.[11] Patients are more satisfied and feel that they have spent more time with their doctors—even though, in some cases, they may spend less time interacting face-to-face.

Family practitioner Jennifer McConnell, who was recently chosen by Maine residents as one of the state's best doctors, offers clinicians the following suggestions for relating to patients in a non-paternalistic way:[12]

> **One of my earlier teachers was caring for my daughter and kept asking me, "What else do you want to make sure we talk about today?" He must have done that four or five times. It was so helpful. It made it much more effective for me to make sure I talked about the things that were concerning me, so I wasn't driving home saying, "Oh man, I forgot to have him look at that rash…." I try to end every visit by asking patients: "Are you clear? Does this feel okay to you? Do you have any other questions or concerns?" If they're confused or unclear or uncomfortable with the final plan, they need to be able to speak up. I always try to let patients know that I'm not going to get mad if they don't do what I think they should do…. I've [also] had patients who weren't comfortable with me as their healthcare provider, and they've moved on to other providers. That's okay.**

As University of Minnesota primary nursing pioneer Marie Manthey, author of *The Practice of Primary Nursing*,[13] has suggested, the non-paternalistic clinician can set the proper tone by asking each patient this simple question: "What needs to happen today for you to feel that your [visit] was successful?" Beginning with this question not only invites the patient to set the agenda but also indicates the clinician's willingness to follow the patient's lead.[14]

Clinician Support for the Expert Patient

Some e-patients develop significant expertise in their conditions. Kate Lorig and her colleagues at the Stanford Patient Education Research Center were the first to identify and study the expert patient. They found that, compared with other patients, expert patients did a much better job of managing their diseases—improving their health status, coping more effectively with fatigue, remaining less dependent on professional care, and managing the many other challenges of their chronic condition. The next step involved the development of a system by which professionals could support expert patients.

In Lorig's expert patient model, experienced patients are recruited and trained to help fellow patients with arthritis and other chronic diseases develop the necessary skills to manage their own conditions. Such patients can serve as facilitators, educators, and supporters of self-managed care. They also encourage their fellow patients to help other patients—and teach them. With Lorig's advice and guidance, the United Kingdom's National Health Service (NHS) has developed its own expert patient program and has made it the centerpiece of their approach to chronic disease management for the 21st century.[15] Although patient-to-patient support can be carried out face-to-face and in groups, online communication makes it possible for the expert patient to guide many more.

The NHS Expert Patient Program is currently being piloted in more than 100 primary care services in England. It will be implemented throughout the NHS by 2007. As Liam Donaldson, chief medical officer of the NHS, observes:[16]

> **Those patients who are confident in their ability to manage their condition are the ones who are likely to have the best outcomes. When patients succeed in resolving problems that they have themselves identified it enhances their sense of self-efficacy. Quality of life improves when the patient rather than their disease is in control. [But], a true partnership will be achieved only with a significant change in the attitude of both patients and healthcare professionals and [in] the way in which they interact with one another…. Patients should be encouraged to enroll by the professionals who care for them…. Anecdotal impressions so far suggest a level of commitment and enthusiasm from patients, healthcare professionals, and managers that will carry the management of chronic disease into a new era of optimism and opportunity.**

How e-Patients Can Help Healthcare

e-Patients are rapidly becoming a valuable new healthcare resource—managing much of their own care, providing care for others, helping professionals improve the quality of their services, and participating in a wide variety of mutually beneficial patient-professional collaborations. But as their new abilities are more widely recognized by clinicians, as they are encouraged to take on new responsibilities, and as the medical work they are doing is integrated with the clinical workflow of professional care, we suspect that they will become an even more valuable resource in the years to come.

Autonomous patients will educate themselves about their medical conditions and will manage more of their own medical care. In so doing, they will operate at a higher level: (1) setting and implementing their own healthcare agendas whenever possible; (2) diagnosing and treating more of their own medical conditions; (3) obtaining more tests and treatments on their own; (4)

storing, organizing, and updating their medical information in more comprehensive and useful ways; and (5) preparing themselves for their interactions with medical professionals.

Patients and caregivers will increasingly take on the responsibility for some activities that were previously provided by professionals. And citizens of all ages will be encouraged and supported to develop their basic medical and clinical skills and knowledge so that they will be prepared to take on the appropriate medical responsibilities when they or a loved one faces a new medical challenge.

Patient-Initiated Quality Improvement Project

One of the last projects Tom Ferguson undertook before his death was a collaboration with the University of Arkansas Medical System (UAMS). As a patient himself, Tom was acutely aware of ways in which the system could better serve patients. While he was undergoing treatment, Tom's powers of observation, curiosity, and dedication to making things better led him to collaborate with his caregivers. White Paper advisor, Charles Smith, MD, has summarized the project that Tom inspired:

The Patient Initiated Quality Improvement Project (PIQIP) at the Myeloma Institute for Research and Therapy (MIRT) was begun as a pilot project in January of 2006. Dr. Tom Ferguson, Dr. Elias Anaissie (his personal physician) and Dr. Charles Smith (Medical Director of UAMS) developed and led this project. Tom had a particular passion for the importance of patient feedback.

The eight-month Phase One pilot served as a proof of concept and helped us understand the complex dynamics involved in using patient feedback to improve the quality of care and customer service. During Phase One we collected two different forms of patient feedback from MIRT patients: (1) extensive feedback on the patient's actual experience, and (2) brief written comments collected via *one-page feedback forms.*

Patient Experience Feedback

We began by selecting 12 patient volunteers. We asked them to keep a journal as they went through each day at the Myeloma Clinic, and then to write up an account of their experiences, sending us this report via e-mail. Patient feedback was reviewed (via e-mail) by Drs. Ferguson, Smith, or Anaissie. After a group e-mail discussion, Dr. Smith was in charge of following up on feedback.

The same complaints came up time after time: waiting time in the clinic was too long and it took entirely too long to get certain IV drugs from the pharmacy. UAMS developed subgroups and improvement projects to correct these problems and the result was much improved flow in these areas.

One consequence of the feedback was that certain areas were bombarded with criticism. That was partly because they were in one of the particularly problematic areas. Morale started to droop under repeated criticism. So we instituted a program in which anytime an employee was singled out with a compliment, we presented her with a certificate and a gift card. Before long, presentation of the gift cards had become an "event" with co-workers gathering around, showing great interest, and congratulating the employee on his recognition. It did wonders for improving morale and making the group more receptive to feedback.

One-Page Feedback Forms

A second type of patient feedback was collected through three patient feedback boxes: one in the waiting area of the myeloma clinic, another in the infusion center and a third in the outpatient transplant area. These boxes were strategically placed so that most or all patients would notice them. They were clearly labeled: "Please tell us how we're doing..." and "Your feedback will help us make things better." Each box contained an open bin of blank feedback forms, a closed, locked ballot-box section for completed forms to be deposited, and a cup-shaped container to hold pens or pencils.

Completed feedback forms are picked up once a week by PIQIP program staff members or patient volunteers. They are keyboarded and delivered to Dr. Smith or Dr. Anaissie. After a group e-mail discussion, Dr. Smith is in charge of handling actionable feedback.

This one-page survey instrument was patterned after the patient feedback system developed by Yolanda Keeling and her colleagues at the University of Texas.

UAMS/Myeloma Institute
Patient Initiated Quality Improvement Project

Dear Fellow Patient,

Thanks for your help! Your feedback and suggestions will help our Patient Feedback for Quality Improvement Committee improve the services provided at the Myeloma Clinic. Our committee is made up of half myeloma patients and half UAMS medical professionals. This project was initated by patients.

You may submit your feedback anonymously if you wish.

Dates of your visit to the Myeloma Clinic___________________________________

1. How would you rate the *quality of the medical care* you've received? (please circle one)

(poor) 0 - 1 - 2 - 3 - 4 - 5 - 6 - 7 - 8 - 9 - 10 (perfect)

2. How would you rate the *quality of the customer service* you've received? (please circle one)

(poor) 0 - 1 - 2 - 3 - 4 - 5 - 6 - 7 - 8 - 9 - 10 (perfect)

For the questions below, please give details and names or identifying characteristics of all staffers involved. If you were particularly pleased, who deserves the credit? If you were dissatisfied with any aspect of your care, please suggest a reasonable resolution. If you need more space, please use the back of this page.

3. What did you like best about this visit?

4. What did you like least about this visit?

5. Please suggest one or more ways we could make things better.

All feedback is confidential--none of the staff members who provide your care will know it came from you. And it will not be recorded in your medical record. You may submit this feedback anonymously, but we hope you'll share your name, UAMS Patient Number, phone number, and e-mail address with us. This will help us clarify any issues you raise and decide on the appropriate action we should take. And we can let you know what was done in response to your feedback.

Many thanks for your help,

---Tom Ferguson, 14-year Myeloma Survivor, patient at the Myeloma Institute, co-chairman of the UAMS/Myeloma Institute Patient Feedback for Quality Improvement Committee

---Charles Smith, MD, Medical Director of UAMS Medical Center, co-chairman of the UAMS/Myeloma Institute Patient Feedback for Quality Improvement Committee

Name ___________________________________ UAMS Patient # __________________

Phone ___________________________________ e-Mail _________________________

[ ] Check here if you would like a member of our committee to contact you.

[ ] Check here if you would prefer that we contact you only if necessary.

If you prefer, you can e-mail the information this form requests to: **patientfeedback@uams.edu**

Confidentiality

Confidentiality is an important element in any effective program for collecting good patient feedback. Many patients are understandably hesitant to criticize clinic staffers, fearing reprisals, strained relations, or less than optimal care. We offer patients providing feedback several levels of confidentiality, ranging from complete anonymity to total transparency. Patients can select one of the four levels of confidentiality that best suits their comfort with disclosing their identity.

Following the pilot, we implemented an active feedback program at the Myeloma Institute in January 2007, using the one-page feedback forms. In addition, the process has been adopted by the Emergency Department and the Radiology Department at UAMS. Patient feedback is critical to the care process and UAMS is using that feedback to improve patient care. We continue to evaluate the process in these three areas to determine whether we want to extend this project to the entire facility. Its role appears to complement a program such as Press-Ganey, which allows easier benchmarking with other similar institutions. The Patient-Initiated Quality Improvement Program allows much more intimate patient involvement in the feedback process and, importantly, rapid response and correction of problems.

Conclusions

As Coulter warns, clinicians must accept patients as partners. If they do not, the healthcare system will be vulnerable to a widespread loss of confidence. But if they do, there is the potential for more patients to help themselves to the health care that they need. The state of health care today is experiencing significant problems. The creation of optimal health care may thus depend on our ability to embrace our first generation of e-patients, providing them with the autonomy, authority, and empowerment they desire and deserve and inviting them to join us in a combined effort to improve healthcare for everyone. It will be only by joining forces with these new colleagues that we can hope to solve the pervasive problems that plague the healthcare system: quality, cost, access, and consumer satisfaction. ■

---

[1] Anne Rogers, Karen Hassell, Gerry Nicholaas, Demanding Patients? Analyzing the Use of Primary Care, (Buckingham, UK: Open University Press, 1999), pp. 102-103.

[2] K.L. White, T.F. Williams, B.G. Greenberg, “The Ecology of Medical Care,” New England Journal of Medicine, 265 (1961): 885-892; Larry A. Green, Barbara P. Yawn, David Lamier, Susan M. Dovey, “The

Ecology of Medical Care Revisited," New England Journal of Medicine, 344 (2001): 2021-2025, <http://content.nejm.org/cgi/content/full/344/26/2021> (Accessed on Aug. 6, 2004).

[3] Michael Hardey, "The 'home page' and the challenge to medicine," He@lth Information on the Internet, no.16 (Aug. 2000): 1-2, <http://www.rsmpress.co.uk/hii/toc16.htm> (Accessed on Apr. 27, 2004).

[4] Hardey, 2000; Michael Hardey, "'E-health': The Internet and the transformation of patients into consumers and producers of health knowledge," Information, Communication & Society, 4 (2001): 388-405.

[5] Hardey, 2001.

[6] e-Patient quotes are from: Tom Ferguson, Susannah Fox, Lee Rainie, Unpublished Online Survey, Sep./Oct. 2000. In order to better understand the e-patient experience to date, we posted an e-patient survey at a website hosted by Princeton Survey Research Associates. Respondents were invited to complete the multiple-choice questions and most used the open-ended text boxes to provide more detail. In all, 1,971 individuals' responses were collected and transmitted to reviewers as three spreadsheets. Follow-up interviews with 19 respondents were completed via e-mail.

[7] Holmes is quoted in Richard Smith, "Why Transparency is Fundamental to [Healthcare] Quality," [Slide Talk], http://bmj.bmjjournals.com/talks/transparency.ppt (Accessed on Sept 2, 2004)

[8] John Wennberg, "Putting the Patient in Charge," Presented at the 1996 Howard A. Paul Symposium, The Smithsonian Institution, Washington, D.C., <http://americanhistory.si.edu/csr/comphist/msbody.htm> (Accessed on Aug. 6, 2004).

[9] Wennberg, 1996

[10] Angela Coulter, The Autonomous Patient: Ending Paternalism in Medical Care, (London: The Nuffield Trust, 2002).

[11] Coulter, 2002.

[12] Jennifer McConnell, quoted in "Two of Maine's Best Rated Doctors Tell You How to Choose and Partner With a Doctor for Best Results," Maine Health Management Coalition, 2002. <http://www.mhmc.info/best/interview.php>, (Accessed on Jun. 20, 2004).

[13] Marie Manthey, The Practice of Primary Nursing: Relationship-Based, Resource-Driven Care Delivery, Second Edition, (Minneapolis: Creative Health Care Management, 2002).

[14] Jane Salvage, "Rethinking Professionalism: the first step for patient-focused care?" The Future Health Worker Project, Institute for Public Policy Research, Jan. 2002, <http://www.ippr.org.uk/uploadedFiles/projects/Rethinking%20Professionalism%20.PDF> (Accessed on Mar. 19, 2007).

[15] The public health white paper, "Saving Lives: Our Healthier Nation," set out the commitment; formal proposals were made in a chief medical officer's report; and a commitment to implement was made in the ten-year NHS Plan published in 2000.

[16] Liam Donaldson, Department of Health, London (liam.donaldson@doh.gsi.gov.uk).

AFTERWORD - 2013

A Model for the Future of Healthcare

Charles Smith, Terry Graedon, Joe Graedon, Alan Green, John Grohol, and Daniel Sands, in collaboration with the SPM Founder's group, Cook's Branch, February, 2013. A version was originally published in the Journal of Participatory Medicine that May.

We are in the midst of one of the most rapid and profound shifts in the history of medicine. When many of today's physicians were still in training, most patients had little or no access to deep information about their health conditions. The Web wasn't available, and medical libraries were off limits. Over the span of a few years, patients have come to have access to more medical information *on their smartphones* than late-twentieth-century Surgeons General had available to them from all their health information resources. The balance of power is shifting, and the amount of health data is exploding. Change is afoot.

In 2007, the first edition of this seminal Robert Wood Johnson White Paper articulated this change and introduced a participatory model for US healthcare. Many discussions had taken place to propose what would be necessary to fix our health system. Tom Ferguson laid out many of the concepts that are needed to "fix our broken healthcare system."

Since the first edition, six years have passed and much has changed, including the current focus on the Patient Protection and Affordable Care Act, with its emphasis on Patient Centered Medical Homes and Accountable Care Organizations (ACO's) as a means to improve quality and lower the cost of care. At the 2013 Cook's Branch meeting, the Founders of the Society of Participatory Medicine decided to add this chapter.

This is truly the decade when patients will take control of their health, given their access to monitoring and communication tools, cutting edge and personalized information, and the power of networking via social media. Acknowledging that 90% of healthcare takes place outside of the physician's office, there is now the probability of a fundamental shift in the dynamic between physicians and patients. The founders of SPM struggled to formulate a mission statement that would define the new relationship, ultimately agreeing on this statement:

> **Participatory Medicine is a movement in which networked patients shift from being mere passengers to responsible drivers of their health, and in which providers encourage and value them as full partners.**

Eric Topol, in *The Creative Destruction of Medicine*, noted, "As will be seen, our go-to source for health and medical information is moving away from our doctor—it is increasingly by crowdsourcing and friend-sourcing our entrusted social network."[1]

We believe that reforming healthcare, first and foremost, must involve activating and engaging patients to become participatory partners. Clay Christiansen and colleagues, in a Wall Street Journal editorial,[2] argued that without more fundamental change in the culture of patients and providers, ACO's are destined to fail. We agree and, for starters, we must tell the public and clinicians how to accomplish this in terms that are understandable, compelling, and logical. As part of our 2013 e Patient Founder's annual meeting, we conducted a brainstorming session to generate ideas about how to effectively activate patients and providers. We proposed three core principles on which the care system should be based:

1. **Patients and providers are encouraged to form partnerships in which each has distinct roles and responsibilities.**
2. **Patients are encouraged to use online resources to network with other patients and learn about their medical conditions, and physicians should help guide and promote discussion of this information.**
3. **To succeed, there must be open, online patient-provider communication and information exchange, including sharing the content of the patient's medical record.**

Adopting New Roles

One practical way to shift physicians' attitudes and practices toward the participatory medicine model for U.S. healthcare is to expand the principles that define the patient centered medical home for primary care (PCMH). The model was originally developed for primary care providers, but represents applicable governing principles for all providers. In fact, the National Committee for Quality Assurance (NCQA), which developed the program, is currently developing a similar program to recognize specialty practices. PCMH points to new roles for both patients and providers. It requires physicians to become more involved in, and supportive of, patient education. It involves a multidisciplinary team approach to care, with each member of the team practicing "at the top of their license." In many practices, these teams will also include mental health professionals, nutritionists, pharmacists, and other professional providers. And it will profoundly alter the provider business model, with widespread adoption and expansion of IT capabilities, more accessible and more frequent modes of communication, and reconfigured incentives and rewards for all stakeholders.

This is a "big idea" with profound implications for patient and provider interaction in the future. No longer will the ambulatory care system be based primarily on an "office visit" model.

Physicians will be reimbursed for “monitoring” their overall practice population and will receive incentive payments for agreed-upon, desired outcomes. Since payment to providers will not be based solely on office visit charges, there will be greater incentives to communicate in other ways, including phone, e-mail, text messages and social media.

The concept of the "medical home" was introduced by the American Academy of Pediatrics in 1967.[3] The National Committee for Quality Assurance (NCQA) created a certification standard for PCMH that was modified in 2011. This list comprises the six basic standards:

1. Enhance access and continuity
2. Population management
3. Plan and manage care
4. Provide self-care support using community resources
5. Track and coordinate care
6. Measure and improve performance

New Patient Roles

For participatory medicine to gain a major foothold, patients will receive guidance as well as tools, information, and data that allow them to be active participants in their care and work effectively with their providers to determine the care they receive. In addition to engaging in online research about their health and their medical conditions, patients will network with online communities to discover which treatments, providers, and facilities work best to address their condition. Through the encouragement of their providers and the network effect of communicating with other engaged patients, they will become more cognizant of their responsibilities toward maintaining their health and will more completely understand their medical conditions. This will become more feasible as home monitoring devices, patient health data visualizations, and associated medical costs become routinely available. In the context of the PCMH, patients will be able to participate in group visits to gain a greater understanding of their illnesses and to provide stimulating interaction with the provider team and patients with similar conditions.

As providers increasingly adopt electronic communication options, clinical care will shift to a “care anywhere” model. Office visits will become much less important and less frequent. Most routine follow up, medication refills, review of blood pressure, adjustment of medications, and other former office-based interactions will become routinely conducted in an online, rather than an office environment. Office visits will only be necessary when a hands-on examination,

interaction or procedure is needed. This new paradigm of care will be much less costly, more patient-centered, more convenient, and will lower barriers to care. These electronic methods of communication may include e-mail, Web-based secure messaging, videoconferencing, mobile phone conversations, text messaging, and instant messaging. It will be commonplace for physicians to give patients a business card at each new patient visit which may include the physician's e-mail address and/or mobile phone number, along with an invitation for the patient to follow up using one of these channels. In addition, office nurses and other staff will often follow up with patients after encounters, either electronically or by phone, to assess progress and learn whether there are any questions or complications. Patients and providers will increasingly adopt the notion that follow up and repeated communication is the norm and that it serves as the best means of achieving optimum outcomes, avoiding diagnostic errors, and obtaining high quality healthcare at the lowest price.

Although we have emphasized that the "new model" will rely less on the office visit, we recognize that some face-to-face encounters will still be necessary to establish and maintain a provider-patient relationship. Indeed, it will be very unlikely that such a partnership can be successful without the personal, human interaction that occurs during an office visit—and, these will remain periodically necessary and beneficial. We also recognize that there will always be a subset of patients who, because of personal preference or special needs, will be best cared for by coming to the office.

Mutual Advisory Roles

In the new model of care physicians will no longer "do" things to patients; they will do things "with" patients as partners. The provider and medical office must be seen as only one of many influences in the patient's health and medical care. This means that the physician is not ordering the patient, but advising them. At the same time the patient will continually advise the provider on how she can provide the best partnership possible to achieve the patient's goals. Getting the patient's input, perspective, and guidance will fundamentally change the way we think about healthcare from a "provider/recipient" relationship to a "care partnership" paradigm.

And patient advising will go beyond the individual patient-provider relationship and the care of the individual patient: they will also advise practices and healthcare systems as a whole. Practices and healthcare systems will no longer be developed for the convenience of the providers or administration. Instead, patients will be invited to participate in patient/family

advisory groups and will be empowered to improve care, practice elements, and health systems.

Acquiring and acting on patient feedback about care is a specific and important way that patients will advise providers. Tom Ferguson often talked about the central role of patient feedback in designing and maintaining the ideal care environment. In addition to joining formal healthcare groups and committees, patients will become accustomed to being invited to provide feedback about their encounters. For example: Who is doing a good versus bad job? How can a specific aspect of a visit or procedure be improved? What helpful comments or advice can you provide to the care team?

These mutual advisory roles will not be limited to the clinical setting, but they will also be critical in addressing clinical research trials. An open flow of information from both the patient and provider perspectives will inform the design of clinical trials from the outset, much like the care process that is being designed by the patient advisory groups. This will mean a stronger patient presence on Institutional Review Boards, which are responsible for monitoring and approving clinical research trials.

Collaboration between Patients and Providers

The traditional means of communication between doctor and patient, with the provider “ordering and advising” will give way to an exchange on a “level playing field” with the patient and provider conversing and exchanging ideas. The provider no longer will be seen as “talking to” the patient and the patient “listening to” the provider and following their directions and orders. Providers will invite patients to engage and participate in their care, encouraging them to be proactive about their health within the context of this provider/patient relationship.

This will be seen as important enough to provide incentives such as co-pay refunds or other financial incentives to patients to encourage them to engage. Providers and patients will begin to “co-create” chart notes, allowing patients the opportunity to contribute to the written documentation in the chart. Decision support “alerts” will be constructed in such a way that they are sent to both the provider and the patient simultaneously, rather than just to the provider. Patients and providers will freely share Web resources that they’ve found to be helpful.

Providers will also ask patients what they think about the suggestions they have made, and what advice they have. They will invite the patient to provide feedback, and will offer them easy-to-use tools for this purpose and commit to responding to the feedback. This feedback will include identifying and reporting diagnostic and other medical errors (this involvement will be welcomed by enlightened providers). Providers will be comfortable admitting when they don't know an answer, and offer to search for answers together with the patient.

Physicians and patients will find new, innovative ways to use social media to communicate with patient groups who have similar conditions. This may include, for example, tweeting "N of 1" trials—i.e., reporting a case from which others may quickly gain benefit. Patients will also share research findings they discover through their online research and interactions with other patients and patient groups. Physicist Michael Nielsen posits, in his remarkable book Reinventing Discovery: The New Era of Networked Science, that the development and accessibility of online tools "can be used to amplify our collective intelligence, in much the way that manual tools have been used for millennia to amplify our physical strength."[4] This is the means by which patient wisdom is already beginning to transform the practice of medicine, and when patients are able to easily obtain and share their clinical data, collective intelligence will be amplified in ways we can barely imagine today.

Physicians will commonly prescribe "information therapy" to give patients the material they need to manage their own healthcare. And similarly, patients will provide physicians with insights they've gleaned from their own experience and from their social network. Most physicians will have interactive practice websites with information for patients such as Frequently Asked Questions, patient education videos about common conditions and a portal for secure messaging as a means of requesting an answer to a clinical question, or renewal of a prescription. The physician websites will be interactive to encourage communication between patients and in addition, physicians will make the charges for and quality of services in their practices more transparent, even posting these on their websites so patients can make more informed decisions when seeking care.

Information Technology and Participatory Medicine

Information technology, through the use of well-designed EMRs and other tools, will play a major role in changing the shape of healthcare. Patients and providers alike will have a variety of user-friendly options to report their experiences. It will be common to use electronic

communication or visit planning applications to negotiate the agenda for a visit before the patient comes into the office to see the physician. This will help to insure optimum use of their time together and, in some cases, will obviate the need for a visit at all. During interactions, a care plan will be collaboratively developed and made available to the patient. There will be easy mechanisms for proactive follow up after every encounter between patient and provider. Physicians will send out letters electronically or in paper form, depending on patient preference, generated from the EMR with lab results and instructions for any needed actions. Physicians and patients will use e-mail or secure messaging to discuss changes in status, provide progress reports, or communicate other important information within the "follow-up" spectrum. In many ways the ultimate quality of the care process will be best judged by the quality and consistency of this follow-up. Rather than requiring the patient to wait "two weeks, two months, or two years" to return to the office, there will an ongoing dialogue of "follow-up conversation" made possible and efficient by online or electronic tools. Many of these conversations can be conducted by physician extenders who can then alert physicians if more direct communication or intervention is needed.

Access to health applications and the Web through connected mobile devices will play a central role in the future of healthcare through use of e-mail, messaging, and mobile applications ("apps"). Many health apps are currently available for use on smart phones and tablets—over 13,000 on Apple's App Store alone. Providers and patients will share lists of useful apps for particular tasks with each other. Patients will use apps to help guide weight loss, promote exercise programs, monitor chronic conditions like diabetes, and much more. Patients with cardiac problems will obtain and transmit an electrocardiogram to their providers. Touch sensitive devices will be used to monitor pulse, respiratory rate, and oxygen saturation. Patients with diabetes will measure their glucose on a connected glucometer and transmit them directly from their devices, usually allowing consultation and medication adjustments to be made without the necessity of an office visit.

As participatory medicine becomes more widely recognized and participatory providers more publicly valued, there will be electronic directories of "participatory providers" that will have a substantial influence on patients' decisions to seek out a particular provider for care.

Education for the New Model of Practice

Traditionally, medical education has focused its efforts on content and procedure training, with little time and energy spent on training for the necessary skills to form effective patient

partnerships. How might we change the educational system to fully embrace participatory medicine? Concepts of participatory medicine will be introduced during the first year of medical school and become a much more prominent part of the future curriculum. Behavior will be modeled for students and they will be taught how to set realistic outcome goals with patients. Students will be educated on “teach-back” methods in their discussions with patients. Medical schools and hospitals will collaborate to form effective clinical teams, including teaching students how to be effective members or leaders of these teams. It will be an exercise in futility if these concepts are taught to students without the routine observation and learning from these collaborative clinical teams who are demonstrating the participatory model of care.

Providing Patient and Provider Incentives for Participatory Medicine

Changing the culture of medical practice will not be easy or quick. The current system is steeped in financial and other incentives that, in many ways, continue to serve as barriers to the evolution of participatory medicine. Providers will begin to be recognized through programs such as “Participatory Provider” lists that will be published to recognize those who practice and encourage participatory medicine, and patients will increasingly seek out providers that practice in a participatory style. Employers and payers will provide financial or other incentives such as preferred parking places, debit cards, or recognition for exhibiting healthy behaviors such as obtaining recommended preventive screening tests, consistent participation in fitness programs, smoking cessation, maintaining target blood sugar levels, optimum blood pressure control, and weight loss.

Changing the Business Model

Moving to the world of participatory medicine will require a new business model. The existing fee-for-service system requires office visits, tests, and procedures for providers and hospitals to thrive. The new world of participatory medicine will only be successful if there are effective financial incentives for patients and providers. Physicians can ill afford to replace office visits with electronic mail messages or e-visits if the payment system doesn’t support these approaches. Management fees, such as per member per month (PMPM) payments or bundled payment mechanisms must be in place to replace fee-for-service. Medicare and Medicaid pilot programs are being implemented to provide primary care physicians a PMPM fee for providing PCMH services. In these plans, it will be more cost-effective for physicians to take a phone call or engage in an e-mail conversation than it will be to ask the patient to come to the office.

Some type of hybrid will likely be needed, but office practices can largely be based on these PMPM systems, with traditional health insurance used to pay for catastrophic care.

Comparing the New with the Old

Here we compare a patient under the "old" or current system to this "new" system we have described:

Case Scenario: Mrs. Smith is 59 years old, is 20 pounds overweight, has an average blood pressure of 145/98 mmHg and a fasting glucose of 135 mg/DL. Her Hemoglobin A1c is 7.2%, indicating that she has previously undiagnosed Diabetes Mellitus. She complains of bilateral knee pain, which makes it hard to exercise. Radiographs of the knees confirm the diagnosis of moderately severe osteoarthritis. She also has complained of hot flashes, insomnia and depression.

Ms. Smith's Care under the "old" (current) system

She comes in for a new patient visit, scheduled for 30 minutes. She arrives at the time of the appointment, but registration is delayed because of a line at the front desk and she doesn't actually arrive in the exam room until about 20 minutes past the appointment time. Her physician is feeling pressed for time because he has a packed schedule. So, he takes as good a history as he can manage in the time allotted for the encounter (which already has put him almost 30 minutes behind for all of his other patients). Since he is rushed, he didn't allow enough time to get the details about the fleeting chest pains she has been feeling, but has tended to discount. The doctor rushes through a physical exam, orders some baseline lab tests, prescribes oral diabetic medication, recommends ibuprofen for her arthritis, refers her to a dietician for weight loss and dietary control of her blood sugar, refers her to a diabetes educator, asks her to start checking her blood glucose levels at home once a day, asks her to start checking her blood pressure three times weekly, and schedules a followup appointment in six weeks. In addition, he recommends that she start taking an antidepressant, fluoxetine (Prozac), and suggests that this may also help her insomnia and anxiety. He doesn't recommend hormone replacement therapy for the hot flashes, but discusses this possibility and they agree to revisit this problem in the future.

She leaves the office feeling like she is making progress towards getting her health issues under control. But, two days after starting the blood pressure medication, she is having

stomach problems and has an annoying cough. She tries to contact the doctor's office but, after trying three times, getting a busy signal, finally getting through, but not receiving a call back from the doctor, she decides it must not be anything to worry about and just continues the medications. But, in addition to the continuing cough, she begins to have increased problems with gastrointestinal discomfort and notices that her stools have turned black. Furthermore, she is starting to get dizzy when she stands up. Her husband becomes alarmed, and takes her to the Emergency Department where she is noted to have blood in her stool and a very low hemoglobin level, in addition to a drop in her blood pressure when she stands up. She receives two units of packed red cells and arrangements are made for an urgent Upper GI endoscopy and colonoscopy to be done. She is noted to have diffuse gastritis, with areas of slowly bleeding stomach mucosa, felt to be due to the large dose of ibuprofen she has been taking.

She stops taking the ibuprofen, her knees start hurting again, but her bleeding stops and her blood pressures have come down into the normal range. Unfortunately, she has been feeling so bad that her attempts at weight loss and exercise have completely gone by the wayside. After six tough weeks, she returns to the doctor's office to "go through the cycle again." She has been taking the fluoxetine, but is still having insomnia and her depression is no better.

Ms. Smith's Care under the "New" System

When Ms. Smith calls the office to arrange for a new patient visit, she is enrolled in the office's patient portal, which allows her to submit her medical history and all of her insurance and demographic information online prior to her visit. When she arrives in the office, she checks in using a computerized kiosk and, since she has been pre-registered, the process takes less than two minutes. She is brought back to the exam room a little ahead of her scheduled visit. The doctor (who has already reviewed her online history) reviews her lab results, places her on the medications noted above, and schedules her for a group visit for diabetes care follow-up in two weeks. In addition, she is given an appointment with the nutritionist and with the diabetes educator for later that week. She is also given a Web address for an online diabetes support group. She is also placed on ibuprofen for her knee pain, and is referred to a physical therapist that specializes in hydrotherapy for osteoarthritis. She is also given information about a certified acupuncturist to help treat her arthritis pain. She is referred to a well-respected website that specializes in home remedies and is encouraged to look over the options listed for treatment of arthritis and knee pain and select any that she is comfortable with. She is asked to log her results for later sharing with the care team.

She is encouraged to use the patient portal to provide blood glucose levels, ask questions, and provide feedback about the effectiveness of her medications. She uses a connected glucometer, along with a new mobile application on her smart phone to upload her blood glucose data directly to her medical team. She is also given an opportunity to join the patient advisory group that the practice is forming, and she is invited to the first meeting three days from now. She is given a follow-up appointment with the physician in three months, but is reminded that scheduling is “open” so she can come back sooner, or anytime she has a need. At the end of the visit, she receives a brief online feedback survey inviting her to give the doctor and his staff feedback about the visit and what suggestions she may have to improve her experience. She notes on this form that the office staff gave her incorrect information about her group diabetes visit and so the outdated information on the patient forms was promptly corrected for future patients.

She is referred to the psychologist associated with the practice to help assess and treat her depression. In addition, she is given a Web address for an online support group for depression, and is encouraged to log on to Psych Central (http://www.psychcentral.com) to review their array of resources and patient tools.

A couple of days later, she develops an annoying cough and is starting to have abdominal pain, so she logs onto the patient portal and asks the office team about these symptoms. The nurse who is monitoring the portal messages contacts the physician, who decides to switch her medication from the ACE inhibitor she is taking to a beta blocker. He also suggests that she stop taking the ibuprofen, try substituting Tylenol, and take a proton pump inhibitor, omeprazole (Prilosec), for a two-week trial. She is asked to log on and provide an online update in 48 to 72 hours.

Two days later, her abdominal pain and her cough have resolved. Her blood pressures are in normal range, but her blood glucose levels are still running from 120 to 150 mg/dL. She is urged to continue the current course and check back online after her nutrition visit, her group visit and her diabetes education visit.

Two weeks later, on the same dose of diabetes medication, with the benefit of her classes and counseling, she logs back in and proudly reports that her glucose levels have come back into the normal range.

She returns to the office three months later, feeling great, in good control of her health issues and confident with the care team’s approach.

Conclusion

Healthcare is broken. Expensive medications and procedures are routinely used without adequate reason; providers are inaccessible and practice defensively; much of the nation's healthcare expenditures are spent on the last few weeks of a patient's life, while a pittance is spent on health promotion and prevention.

Reform will not be achieved with ACO's alone but by meaningful activation of patients and providers who practice in a "medical home" environment. Communication between patients and providers will explode in open dialogue, full of follow-up adjustments and readjustments that usually won't require office visits. Electronic medical records, routine, daily use of the internet, and extensive use of smart phones will harness the power of computing to eliminate many of the errors and omissions common in today's healthcare environment. The business model of fee-for-service will give way to a PMPM system that will support office-based physicians who practice in a patient centered medical home mode.

It will be a new day and healthcare CAN be fixed! Are you ready? Will you help?

---

[1] E. Topol. *The Creative Destruction of Medicine: How the Digital Revolution Will Create Better Health Care*, Basic Books, 2011.

[2] C. Christensen, J. Flier, and V. Vijayaraghavan. "The Coming Failure of 'Accountable Care,'" *The Wall Street Journal*, May 15, 2013.

[3] Wikipedia: History of Medical Home.

[4] M. Nielsen. *Reinventing Discovery: The New Era of Networked Science*, Princeton University Press, 2011.

Further Reading:

C. Christensen, J. Grossman, and J. Hwang. *The Innovator's Prescription: A Disruptive Solution for Health Care*, McGraw-Hill, 2008.

About the Author

Pioneering physician, author, and researcher Tom Ferguson studied and wrote about the empowered medical consumer since 1975 and about online health resources for consumers since 1987. In 1993 he organized the world's first conference devoted to computer systems designed for medical consumers. After attending Reed College, earning a Master's Degree in creative writing from San Francisco State University, and a medical degree from Yale University School of Medicine, he launched a prolific career in consumer focused medical writing as founder of Medical Self Care magazine.

From 1980 to 1996 he authored or co-authored over a dozen books and was section editor for health, medicine, and self-care for the *Whole Earth Catalogue*.

Dr. Ferguson virtually led the movement to advocate informed self-care as the starting point for good health, and to promote a new kind of relationship between knowledgeable medical consumers and medical professionals. His goal was to encourage medical professionals to treat clients as equal partners in achieving better outcomes and change the entrenched practices of the traditional top-down hierarchy of the doctor-patient relationship. With the advent of broad access to the internet, Dr. Ferguson's long history of advocacy of information-empowered medical consumers positioned him to be a leading proponent of online health information resources.

Following his own philosophy, he survived 15 years with multiple myeloma, far exceeding typical expectations. He relentlessly pursued strategies for both self-care and the newest research and experimental practices for controlling this aggressive cancer. During that time, between relapses and debilitating treatments, he led a migration of medical consumer information to the internet, lectured widely on the emerging field of "health informatics", and earned a global reputation as a true innovator and pioneer in the field. In 1999 he was one of four to be recognized as an "Online Health Hero", an award given by the Intel Corporation's Health Initiative Project.

In recent years, he has served as a Senior Research Fellow for Online Health at the Pew Internet and American Life Project in Washington D.C. and a consultant to the Robert Woods Johnson Foundation. He is also an Adjunct Associate Professor of Health Informatics at the University of Texas Health Science Center in Houston, a Senior Associate at Boston's Center for Clinical Computing, a medical computing think-tank associated with Harvard Medical School and Beth Israel Deaconess Hospital, and most recently joined the University of Arkansas Medical Sciences Center as an adjunct faculty where he initiated a patient centered quality improvement program at the Myeloma Institute for Research and Therapy.

Tom Ferguson, M.D. died April 14, 2006 at the UAMS medical center hospital in Little Rock, Arkansas, where he was undergoing treatment for multiple myeloma.

From the obituary published in the Austin-American Statesman, April 19, 2006 and posted online at: http://doctom.com/.

About The e-Patient Scholars Working Group

For more than five years Tom Ferguson gathered together a group of friends, colleagues and thought leaders in the field of online health. This collaboration was helpful for Tom as he formulated his thoughts about e-patients and this White Paper. It was enormously exciting for the rest of us who got to exchange ideas with people outside our usual contacts. Tom fed us, entertained us, stimulated us and helped us imagine a new paradigm for health care.

Tom's death devastated us. He was the glue that held us together. We undertook the completion of this White Paper as an expression of our love for Tom and respect for his vision. We hope we have captured the critical elements that Tom had identified.

Susannah Fox, Director of Research for the Pew Internet & American Life Project and principal author of the Pew Internet & American Life Project's survey reports on e-patients and online health.

Gilles Frydman, Founder & President of the Association of Cancer Online Resources www.acor.org, the largest online support group for cancer patients.

Joe Graedon, M.S., pharmacologist & **Teresa Graedon, Ph.D.,** medical anthropologist. Together they write consumer drug books and a syndicated consumer health newspaper column and host a public radio show, all called "The People's Pharmacy." (www.peoplespharmacy.com)

Alan Greene, M.D., Stanford pediatrician & **Cheryl Greene,** website producer. In 1995 they launched their pediatric website, www.drgreene.com, the first physician website on the Internet. Dr. Greene has been recognized by Intel's Internet Health Initiative as one of four pioneering Online Health Heroes "who are developing innovative and compelling new ways to use the Internet to advance public health."

John Grohol, Psy.D., Pioneer in online mental health and founder of PsychCentral.com. He co-founded Mental Health Net in 1995, one of the largest mental health portals at that time. Dr. Grohol is the author of *The Insider's Guide to Mental Health Resources Online* (Guilford, 2003).

Dan Hoch, M.D., Massachusetts General Hospital. Leading physician pioneering online support group for his neurology patients.

John Lester, Founder, www.braintalk.org. John is now working with Linden Labs in Second Life as Community Manager, helping to build better tools for communication and education. More information at: http://secondlife.com/education. Also visit: http://brain.hastypastry.net/OSN_2005_Second_Life/

Daniel Z. Sands, M.D., MPH, Senior medical informatics director in Cisco's healthcare group. Also practices at Division of General Medicine at Beth Israel Hospital and teaches at Harvard Medical School. The leading expert on doctor-patient e-mail.

Charles Smith, M.D., Medical Director, University of Arkansas for the Medical Sciences (UAMS).

Connie Smith, cancer patient at the UAMS Cancer Center.

Additional Contributors

Elias Anaissie, M.D., Professor of Medicine at the University of Arkansas for the Medical Sciences.

Anna Bryan, assistant and organizer par excellence

Lewis Engle

Jan Guthrie, MLS, leading freelance librarian for e-patients (www.thehealthresource.com)

Jon Lebkowsky, CEO of Polycot Consulting and cofounder of EFF-Austin, originally a chapter of the Electronic Frontier Foundation and then an independent organization

Edward Madara, Director, American Self-Help Clearinghouse (www.selfhelpgroups.org)

Karen Parles, Webmaster, www.lungcanceronline.org

Lee Rainie, Founding Director of the Pew Internet & American Life Project.

Richard Rockefeller, M.D, Health Commons Institute, Falmouth, ME
